# Syntheses of mono-acylated luteolin derivatives, evaluation of their antiproliferative and radical scavenging activities and implications on their oral bioavailability

**DOI:** 10.1038/s41598-021-92135-w

**Published:** 2021-06-15

**Authors:** Stephen Lo, Euphemia Leung, Bruno Fedrizzi, David Barker

**Affiliations:** 1 School of Chemical Sciences, University of Auckland, 23 Symonds St, Auckland, 1010 New Zealand; 2 Auckland Cancer Society Research Centre, University of Auckland, Auckland, 1023 New Zealand; 3 MacDiarmid Institute for Advanced Materials and Nanotechnology, Wellington, 6012 New Zealand

**Keywords:** Medicinal chemistry, Organic chemistry

## Abstract

Luteolin is a flavonoid found in a wide range of plant materials, including commonly eaten fruits and vegetables. It displays a wide range of biological activities but is known to have poor bioavailability. In this study, ten different mono-acyl (nine 5-*O*-acyl and one 7-*O*-acyl) derivatives of luteolin were synthesised for the purpose of improving bioactivity and bioavailability, and therefore enhance their therapeutic potential. The antiproliferative activity of these derivatives was assessed against the HCT116 colon cancer and MDA-MB-231 breast cancer cell lines using a ^3^[H] thymidine incorporation assay. The radical scavenging activity of these derivatives against 2,2′-azino-bis(3-ethylbenzothiazoline-6-sulfonic acid) (ABTS) radical cation and 2,2-diphenyl-1-picrylhydrazyl (DPPH) radical using Trolox as a standard, was also assessed. Some of these derivatives were found to have improved antiproliferative activity with comparable radical scavenging activity compared to luteolin. Increased lipophilicity has been shown to increase the bioavailability of flavonoids implying these analogues will also have increased bioavailability.

## Introduction

**Figure 1:**
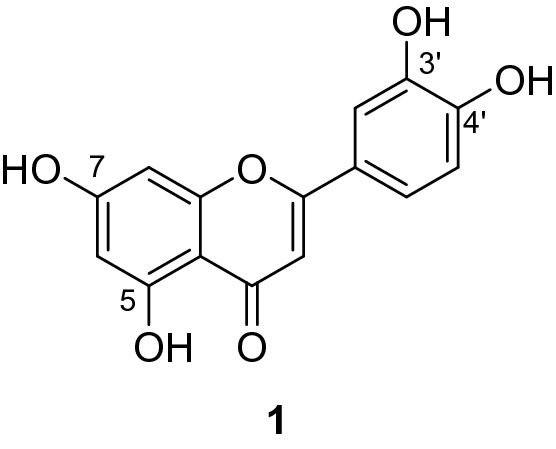
Structure of luteolin, a tetrahydroxyflavone.

Luteolin (**1**) is a natural flavonoid that is present in many plant products, including fruits and vegetables common to the human diet^1,2^. Structurally, the compound is classified under the flavone subclass and consists of four hydroxy groups positioned at the 5-, 7-, 3′- and 4′- positions (Fig. 1). Luteolin displays a wide range of bioactivities which strongly suggests that the compound has potential for further development into agents with health-promoting effects. These include antioxidant^3,4^, anti-inflammatory^5,6^, antibacterial^7,8^, antiviral^9^, neuroprotective^10,11^ and cardio protective^12^ effects. Furthermore, studies have reported luteolin’s ability to inhibit proliferation against many cancer cell lines, and therefore have potential utility in the treatment of cancer^13–20^.

Despite these promising bioactivities, a problem that is ubiquitous to all flavonoid compounds which severely limits their therapeutic ability is their poor oral bioavailability or ability to reach site of action after oral administration^21–23^. The hydroxy groups on flavonoids are susceptible to metabolic activity that result in metabolites that are easily eliminated from the body before the desired therapeutic activity is exerted^23^.

Previous studies on derivatisation of flavonoid compounds, by making them more lipophilic, has proven to be a useful strategy to improve both their bioactivity^24,25^ and properties related to the bioavailability^26–29^. Proposedly, similar derivatisations on luteolin could result in such improvements as well. However, considerations were made to ensure these compounds possessed better overall therapeutic potential (balance of bioactivity and bioavailability). It has been found that the desired antioxidant capacity of luteolin was significantly better compared to the other flavone compounds, chrysin (without 3′- and 4′-OH groups) and apigenin (without 3′-OH), which suggests that the catechol group plays an important role towards this activity^30^. It was decided that the derivatisation of 5-*O* or 7-*O* positions would result in luteolin derivatives with better overall properties and therefore therapeutic potential. This report describes the syntheses of a series ten mono-acyl (nine 5-*O*-acyl and one 7-*O*-acyl) derivatives of luteolin. Subsequently, these derivatives were evaluated for their antiproliferative and radical scavenging activities to determine the effects that substitution of the hydroxy groups with the more lipophilic moieties had on its therapeutic ability.

## Results and discussion

### Syntheses of mono-acyl luteolin derivatives

**Scheme 1:**
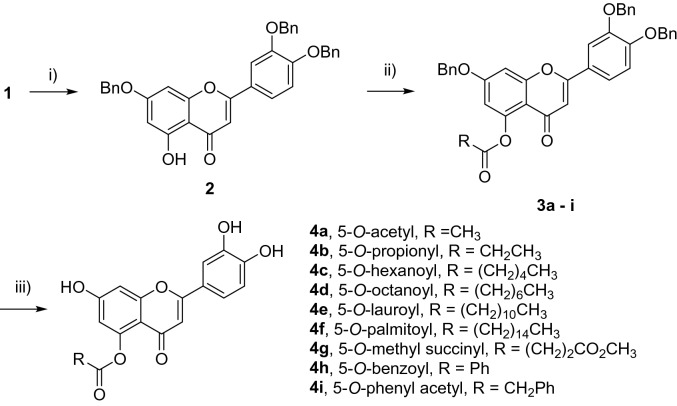
Syntheses of 5-*O*-acyl luteolin compounds; (i) BnBr, K_2_CO_3_, DMF, 100 °C, 24 h, 76%; (ii) Et_3_N, acyl chlorides, CH_2_Cl_2_, r.t., 24 h, **3a** 82%, **3b** 68%, **3c** 70%, **3d** 55%, **3e** 68%, **3f** 82%, 3 h 80%, **3i** 65%; (iii) Pd(OH)_2_/C or Pd/C, H_2_, THF, r.t., 24 h, **4a** 86%, **4b** 97%, **4c** 91%, **4d** 85%, **4e** quantitative, **4f** 76%, **4g** 32% over two steps, **4h** 78%**, 4i** 54%.

The first step towards the syntheses of the 5-*O*-acyl luteolin compounds was the benzylation of the hydroxy groups at the non-hydrogen bonded 7-, 3′- and 4′- positions of compound **1**. By modifying a reported procedure, the tri-*O*-benzyl luteolin (**2**) was afforded in 76% yield which is a significant improvement compared to the original procedure (Scheme 1)^24^. On proton (^1^H) nuclear magnetic resonance (NMR) spectrum of this compound, the signal at δ = 12.78, which had previously been identified as the 5-OH signal^31^, was the only hydroxy proton signal observed. This confirmed that the 5-OH site was the only position available for acylation. The addition of a range of acyl chlorides to compound **2**, in the presence of triethylamine afforded the 5-*O*-acyl tri-*O*-benzyl luteolin derivatives (**3a**–**i**) in 55–82% yields (Table 1). These tetra-substituted luteolin products were immediately identified on thin layer chromatography (TLC) by their blue fluorescence under both short (254 nm) and long (365 nm) wavelengths. From their ^1^H NMR spectra, the 5-OH proton peaks were no longer present whilst the alkyl or aliphatic protons were observed in the region between δ = 0.88–2.77. Subsequent palladium catalysed hydrogenation of these compounds, catalysed by either Pd(OH)_2_/C or Pd/C, resulted in the removal of the benzyl groups from all three positions and the 5-*O*-acyl luteolin derivatives (**4a–i**) were afforded in yields of between 54 and 100%. The only exception to this procedure was for derivative **4g**, as intermediate **3g** was inseparable from unreacted **2** and so this crude mixture was hydrogenated to give the 5-*O*-methyl succinyl luteolin derivative **4g** in 32% yield over 2 steps. All 5-*O*-acyl luteolin derivatives were immediately identified on TLC by their light blue fluorescence when observed under 365 nm. Significant differences in the chemical properties of the derivatives compared to quercetin were observed. The melting point of luteolin was reported at 328–330 °C^32^. The addition of the acyl groups on the 5-*O* position of luteolin had greatly reduced the melting points to between 149 and 198 °C. The smaller Rf values of derivatives **4a** and **4b**, with short acyl carbon chain lengths, as well as **4g**, with the polar methyl succinyl group (Rf values = 0.29, 0.43 and 0.26, respectively with 1:3 petroleum ether:EtOAc), suggested that these compounds were more polar compared to parent compound **1** (Rf = 0.57, 1:3 petroleum ether:EtOAc). The Rf value of derivative **4c** (Rf = 0.57, 1:3 petroleum ether:EtOAc) suggested that it had similar polarity to **1**; whilst derivatives **4d**–**4f** (Rf = 0.39–0.53, 1:2 petroleum ether), with longer acyl carbon chain lengths, as well as **4h** and **4i** (Rf = 0.39 and 0.55, respectively with 2:1 petroleum ether:EtOAc) consisting of the aromatic groups, displayed Rf values which suggested that these compounds were less polar compared to **1**. Whilst compound **1** was insoluble in tetrahydrofuran solvent, it was found that all luteolin derivatives could be solubilised in this solvent and indicated that the 5-*O*-acylations had resulted in more organic soluble compounds.

**Table 1:** The IC_50_ values of luteolin and 5-*O*-acyl luteolin derivatives against the two cancer cell lines.

Compound	IC_50_ (µM)	
	HCT116	MDA-MB-231
**1**	** > 10**	** > 10**
**4b**	7.02 (± 1.53)	7.20 (± 0.46)
**4c**	> 10	> 10
**4d**	8.14 (± 1.35)	7.11 (± 1.11)
**4e**	9.10 (± 0.35)	6.89 (± 0.88)
**4f.**	8.84 (± 0.41)	7.22 (± 1.1.13)
**4 g**	6.17 (± 0.92)	4.87 (± 0.23)

**Scheme 2:**
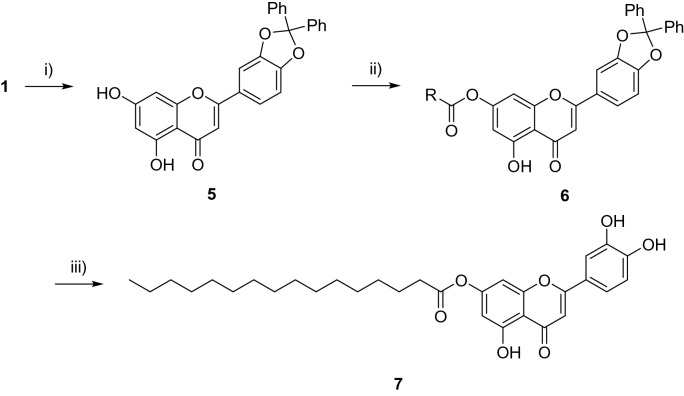
Synthesis of 7-*O*-palmitoyl luteolin; (i) Ph_2_CCl_2_, PhOPh, 60–175 °C, 24 h, 39%; (ii) Et_3_N, palmitoyl chloride, CH_2_Cl_2_, r.t., 24 h, 75%; (iii) Pd/C, H_2_, THF, r.t., 4 d, 34%.

It was also decided to synthesise the 7-*O*-palmitoyl luteolin (**7**) as a model for 7-*O* derivatised luteolin compounds. Due to poor bioactivity and solubility, as described in the next section, this was the only 7-*O*-acyl luteolin synthesised in this study. The synthesis of this compound began by reacting **1** with diphenyldichloromethane, as strategy for protecting the catechol group with the diphenyldioxol moiety, and compound **5** was obtained albeit in a low yield of 39% (Scheme 2). On ^1^H NMR spectrum, the δ values were in agreement with that reported in literature^33^. Furthermore, the 5-OH and 7-OH proton signals were observed at δ = 12.87 and 10.89, respectively, and thus these hydroxy groups remained unaltered. It was proposed that subsequent acylation of compound **5** would favourably and selectively occur at the desired 7-*O* position, rather than at the 5-*O* position. This was rationalised from the computational study that demonstrated the differences in bond dissociation energy between these two hydroxy sites^34^. Furthermore, despite employment of excess benzylating agent in the previous reaction to form compound **2** in this study, little to no tetra-*O-*benzyl luteolin was obtained, which further confirmed the reactivity of 5-OH was much lower compared to 7-OH. It was decided to use a limited 0.9 molar equivalence of palmitoyl chloride in the react with compound **5**, to reduce the possibility of di-acyl luteolin formation. It was found that this had successfully resulted in compound **6** with good 75% yield. The compound was immediately confirmed by the presence of the 5-OH proton signal observed at δ = 12.77, the absence of the 7-OH proton signal as well as the aliphatic proton signals on ^1^H NMR spectrum. Subsequently the removal of the diphenyldioxol protecting group was achieved through the hydrogenation, catalysed by Pd/C over 4 days, to afford the desired derivative **7**. On TLC, this compound displayed the same yellow fluorescence as that described for the 5-*O* acyl derivatives. As expected, derivative **7** was much less polar (Rf = 0.28, 2:1 petroleum ether:EtOAc) and had a lower melting point (202–205 °C) compared to compound **1**.

### Antiproliferative studies against HCT116 and MDA-MB-231 cancer cell lines

Cancer was the leading cause of death in 2020, out of which the highest figures were related to breast cancer (1.80 million deaths) and colorectal cancer (935,000 deaths)^35^. The magnitude of this health issue warrants continued efforts towards developing novel or enhancing therapeutic solutions for its treatment. Luteolin is known to have activity against the colon HCT116 and breast MDA-MB-231 cancer cell lines whilst having little effect on the HEK293 normal kidney cell line^13,18,19^. Therefore, these three cell lines were chosen for evaluation on the antiproliferative activity of **1** and the mono-acyl derivatives (**4a–i** and **7**) using a previously reported protocol^36^.

**Figure 2:**
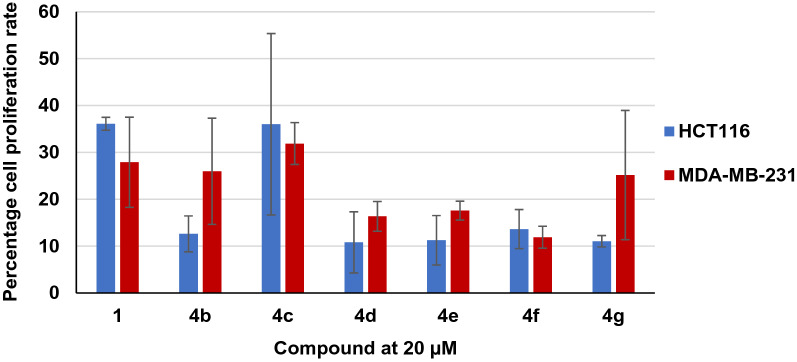
Cell proliferation rate (%) of HCT116 ad MDA-MB-231 cancer cell lines treated with compounds (20 µM), in comparison to 100% proliferation rate when cells were treated with control.

The first assessment was conducted by treating the cells with a single dose of flavonoid **1** and the derivatives at 20 µM. It was found that luteolin **1** reduced the cell proliferation rate of HCT116 and MDA-MB-231 to 36.4 (± 1.4)% and 27.9 (± 9.6)%, respectively, compared to 100% cell growth rate when treated with control (Fig. 2). Unfortunately compounds **4a**, **4 h**, **4i** and **7** displayed cell proliferation rates much higher than that of luteolin and were therefore omitted from further analyses. Compounds **4b**, **4d**, **4e**, **4f.** and **4 g** displayed either better antiproliferative activity against HCT116 cell lines compared to luteolin with proliferation rates at 12.6 (± 3.8)%, 10.8 (± 6.5)%, 11.2 (± 5.3)%, 13.6 (± 4.2)% and 11.0 (± 1.2)%, respectively, and are 2.7- to 3.5-fold increased in activity in comparison to **1**. Compound **4c** appears to have similar proliferation rate against HCT116 compared to luteolin, however the variation of this result was high. Compounds **4d**, **4e** and **4f** displayed better antiproliferative activity against MDA-MB-231 cell lines compared to luteolin with proliferation rates at 16.4 (± 3.2)%, 17.6 (± 2.0)% and 11.9 (± 2.3)%, respectively, and have a 1.6- to 2.3-fold increase in activity compared to **1**. Compounds **4b**, **4c** and **4g** displayed relatively similar proliferation rate to luteolin, although the high variability of **4g** against MDA-MB-231 was noted. On the normal HEK293 cell line it was found that luteolin **1** and all synthetic derivatives (**4a–i** and **7**) slightly reduced cell proliferation (82–99% growth), with the greatest inhibition, 82 (± 0.9)% being seen with compound **4e**, showing that the derivatives had selectivity for cancer cell lines.

The IC_50_ values of the more active derivatives (**4b**–**g**) were then elucidated using various concentrations between 0 and 10 µM (Table 1). The IC_50_ of luteolin (**1**) against both cell lines was above 10 µM (Table 1). Similarly, the IC_50_ of **4c** could not be determined at 10 µM and below. However, the rest of the five derivatives **4b**, **4d**, **4e**, **4f** and **4g** displayed decreased IC_50_ with values below 10 µM and indicated significant improvement in the desired antiproliferative activity compared to the starting flavonoid **1**. It was found that derivative **4g**, consisting of the more polar methyl succinyl moiety, displayed the lowest IC_50_ values against both cancer cell lines at 6.17 (± 0.92) µM and 4.87 (± 0.23) µM, respectively.

### ABTS and DPPH radical scavenging activity studies

Flavonoids, including luteolin, are well known for their antioxidative effects which largely contributes to their therapeutic potential^3,4,30^. Since the catechol 3′- and 4′-OH groups of luteolin (**1**), and other flavonoids have been found to contribute to this effect, the purpose of mono-acyl derivatisations at the 5- and 7-*O* positions in this study was to prevent this effect from being eliminated^30,37^. Therefore, it is important to determine whether this derivatisation strategy had resulted in the desired retention, or even improvement, of its antioxidant effects. The 2,2′-azino-bis(3-ethylbenzothiazoline-6-sulfonic acid) (ABTS) radical cation and 2,2-diphenyl-1-picrylhydrazyl (DPPH) radical, are two stable free radicals often used as *in* vitro models for evaluating the radical scavenging ability, and therefore antioxidant capacity, of the compounds^38,39^. The radical scavenging activities of parent compound **1**, and its derivatives **4a**–**i** and **7**, were thus assessed against these two stable radical species and was conducted using the previously reported protocols^39–41^.

**Figure 3:**
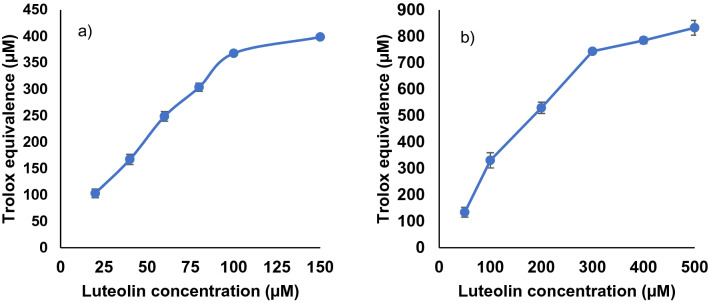
The Trolox equivalence (TE) of luteolin at various concentrations against (**a**) ABTS radical cation; and (**b**) against DPPH radical.

In these assay studies, Trolox was used as a reference antioxidant and the radical scavenging activity of the samples were quantified as the Trolox equivalence (TE). The Trolox standard curves were generated using 6 different concentrations ranging between 50 and 400 µM against the ABTS radical cation and 7 different concentrations ranging between 50 and 1000 µM against DPPH radical. These standard curves were developed in triplicate measurements and had a linearity of above 0.99, with exception to one curve with linearity of above 0.97 (Supplementary Tables S1 and S2). The radical scavenging activity of flavonoid **1** and the derivatives were then measured using 6 different concentrations ranging between 20 and 150 µM against the ABTS radical cation and between 50 and 500 µM against the DPPH radical. The TE of luteolin against both radicals are shown in Fig. 3.

The radical scavenging activity of derivatives **4a**–**i** were then conducted (Tables S3 and S4 in supplementary information). Unfortunately compound **7** was insoluble in the dimethyl sulfoxide solvent and therefore the radical scavenging activity could not be obtained. It was found that all derivatives (excluding compound **7**), at each concentration, displayed radical scavenging activity, which suggested that the derivatisation of the 5-*O* position did not eliminate this activity. These results are expressed in this report as luteolin’s equivalence activity as found on Table 2. In general, the activities of the short to long acyl derivatives (**4a**–**f**) as well the more polar derivative **4g**, were not significantly different compared to that of luteolin (*p* > 0.05), although compounds **4h** and **4i** were generally found to be significantly different (*p* < 0.05) compared to luteolin suggesting that the phenyl moieties resulted in slightly decreased radical scavenging activity.

**Table 2:** Luteolin’s equivalence radical scavenging activity of the 5-*O*-acyl luteolin derivatives against ABTS radical cation and DPPH radical at various concentrations; *results obtained from in duplicate assessments instead of triplicate; ** significantly different (*p* < 0.05).

Compound	Sample concentration (µM)						
	**ABTS**	**20**	**40**	**60**	**80**	**100**	**150**
	**DPPH**	**50**	**100**	**200**	**300**	**400**	**500**
**1**	**ABTS**	1.00	1.00	1.00	1.00	1.00	1.00
	**DPPH**						
**4a**	**ABTS**	0.90	1.02	0.94	0.98	1.00	0.99
	**DPPH**	0.86	0.94	0.97	0.88	0.94	0.95
**4b**	**ABTS**	1.09	1.10	0.97	0.94	0.97	0.98
	**DPPH**	1.18	0.97	1.16	0.97	1.02	0.97
**4c**	**ABTS**	1.19	1.11	0.95	0.93	1.02	0.99
	**DPPH**	0.95	0.86	1.17	1.01	1.04	0.99
**4d**	**ABTS**	1.14	1.00	0.84**	0.87	0.93	0.98
	**DPPH**	1.17	1.02	0.99	0.97	0.97	0.97
**4e**	**ABTS**	1.18	1.05	0.97	0.96	0.96	1.00
	**DPPH**	1.27	0.95	1.05	0.95	1.00	0.96
**4f.**	**ABTS**	0.74	0.67	0.75**	0.67**	0.74**	0.87
	**DPPH**	0.88	1.02	0.98	0.91	0.96	0.97
**4 g**	**ABTS**	0.84	0.80	0.70	0.73**	0.68	0.79
	**DPPH**	1.16	0.76	0.98	0.65	0.82	0.85
**4 h**	ABTS*	0.76	0.83	0.79	0.79**	0.77**	0.85**
	**DPPH**	1.12	0.81	0.83**	0.79**	0.85**	0.87**
**4i**	**ABTS**	0.41**	0.62**	0.91	0.72	0.72**	0.83
	DPPH*	1.01	0.92	0.79**	0.70**	0.73**	0.80**

### Implications of derivatives on bioavailability

Bioavailability is defined as “the rate and extent to which the active ingredient or active moiety is absorbed from a drug product and becomes available at the site of drug action”^42^. This parameter is important as the effectiveness of a therapeutically active compound is also dependent on its ability to reach the site of action, from site of administration, and exert the desired effects. Oral bioavailability is often measured by the amount of active compound in the systemic circulation (plasma drug concentration over time or area under the curve) after oral administration, and is compared to that after the active compound is intravenously administered^43,44^. This relative measurement assumes that the availability of drug at site of action is proportional to that found in the systemic circulation.

Following oral administration, the drug compound(s) must pass through many compartments (such as the stomach, gastrointestinal lumen, the intestinal cells, the hepatic portal vein and the liver) before it reaches the systemic circulation^45^. At various stages, there is extensive exposure to metabolic activity (first pass effect), whereby the structure can be enzymatically altered. For example, conjugation of a drug compound results in its methylation, sulfation and glucuronidation. These metabolite products are no longer therapeutically active and are easily eliminated from the body.

Flavonoids are known to have poor bioavailibility^21–23^. In the case of luteolin, two studies reported that the oral bioavailability in rat models was 4.1% and 26%^46,47^. The low bioavailability was primarily attributed to first pass metabolism as the free hydroxy groups of luteolin are susceptible to conjugation, particularly glucuronidation and sulfation^22,48–50^. Furthermore, studies using model monolayer intestinal (Caco-2) cells, found that luteolin was subject to efflux mechanisms, implying that the compound is prone to being pumped back to the lumen and eliminated^51,52^.

To circumvent this problem, previous studies have suggested that ester derivatives of flavonoids could improve bioavailability, or properties related to it. Lambert et al., found that the area under the curve of total epigallocatechin gallated (EGCG) in mice plasma was 2.4-fold higher when peracetylated EGCG was orally administered compared to non-derivatised EGCG^26^. Furthermore, the elimination half-life of EGCG in plasma had increased by 2.2-fold when treated with peracetylated EGCG compared to EGCG. Biasutto et al. studied the amount of pentaacetylated and 3′-Boc amino acid tetraacetylated quercetin derivatives were able to pass from the apical side to basolateral side of epithelial cells (including Caco-2) and that the total unconjugated quercetin compounds available in the basolateral compartments were generally higher for the more lipophilic derivatives (0.4–22.2%) compared to quercetin (1.0%)^27^. Wen et al. found that the methylated derivatives of flavones displayed five- to eightfold higher permeability in the Caco-2 cells^28,53^. It was also found that these methyl moieties provided substantial protection from hepatic metabolic activity compared to the non-methylated flavones. This increased metabolic stability of these compounds is owed to the methyl groups masking the hydroxy groups and limits their ability to become conjugated.

These studies suggest that the flavonoid derivatives may act to improve bioavailability in two ways. The first is that it can act as prodrugs, where upon metabolism of the derivatised flavonoid, the non-derivatised form is generated. The second is that the moieties offers metabolic protection from metabolism, and thus it becomes less prone to elimination. It is proposed that the mono-acyl luteolin derivatives in this study, could also act in similar fashion to increase the bioavailability of unconjugated bioactive luteolin compounds (both luteolin and mono-acyl derivatives). It remains as part of further studies to quantify the oral bioavailability of these compounds.

## Conclusion

In this study, nine different 5-*O*-acyl and one 7-*O* acyl luteolin derivatives were synthesised and assessed for their antiproliferative activity and radical scavenging activity. It was generally found that the 5-*O* acyl derivatives with aliphatic chains displayed better antiproliferative activity against the cancer cell lines as well as retained similar radical scavenging activities compared to luteolin This suggested that the antiproliferative activity of these derivatives is unrelated to the radical scavenging activity. It was clearly observed that luteolin derivatives with phenyl groups had significantly reduced both the antiproliferative and radical scavenging activity. Derivative **7**, which was synthesised as a representative of 7-*O* acyl luteolin compounds did not possess the desired solubility for the proposed bioactivity studies, and therefore these properties could not be determined. Out of all mono-acyl derivatives in this study, the 5-*O*-methyl succinyl luteolin (**4g**), possessing the lowest IC_50_ values against the cell lines, stood out as the compound with most therapeutic potential. This implies that further exploration on luteolin compounds, derivatised with slightly more polar moieties, could provide better therapeutic potential. Previous studies on flavonoid esters suggest that the mono-acyl luteolin derivatives may act as prodrugs to generate luteolin upon metabolism or protect it from metabolic activity. This implies that the oral bioavailability of unconjugated luteolin compound could be increased through these means. Further studies are required to determine the true bioavailability of these compounds.

## Methods

### Synthetic procedures

The synthetic procedures for synthetic compounds and their characterisation are found in the supplementary information. The ^1^H and ^13^C NMR spectra of novel compounds are also provided.

### Antiproliferative activity procedures

The antiproliferative studies on HCT116 and MDA-MB-231 cancer cell lines and the normal HEK293 cell line were conducted using the [^3^H]-thymidine incorporation assay method as detailed in the report by Leung et al.^36^ Essentially, the method was conducted by seeding 3000 cells in each well of the 96 well plates, with varying concentrations of inhibitors for 3 days. [^3^H]-thymidine is added to the cells and incubated for 6 h. The cells were then counted using Trilux/Betaplate counter showing percentage of the cells with [^3^H]-thymidine incorporated into the DNA helix. The cell lines were first treated with 20 µM of each of the compounds in DMSO and the antiproliferative activity was determined as cell growth percentages relative to the 100% growth in control. The IC_50_ of the derivatives against the cell lines were then determined using concentrations of 10 µM or below.

### Radical scavenging activity procedures

The radical scavenging activity procedures are described below. The radical scavenging activity of the mono-acyl derivatives of luteolin are found in the supplementary material.

#### Preparation of Trolox standard and luteolin derivative solutions

Trolox standards were prepared as previously described^39^, by dissolving in DMSO to give a 2 mM stock solution which was stored in − 80 °C until use. To generate the standard curve against ABTS, the stock solution was diluted with DMSO to obtain concentrations of 40, 60, 80, 100, 200 and 400 µM. For the standard curve against DPPH, the stock solution was diluted with DMSO to obtain concentrations of 50, 100, 200, 400, 600, 800 and 1000 µM. The samples were prepared by dissolving luteolin derivatives in DMSO to make 2 mM stock solution. For ABTS radical scavenging assay, the sample stock solutions were further diluted to concentrations of 20, 40, 60, 80, 100 and 150 µM. For DPPH radical scavenging assay, the sample stock solutions were then further diluted to concentrations of 50, 100, 200, 300, 400 and 500 µM.

#### Preparation of ABTS radical cation solution and DPPH radical solution

The ABTS radical cation solution was prepared according to the reported procedure^39,40^. A 20 mM acetate buffer was first prepared by adding CH_3_COONa in H_2_O and then adjusting the pH of this buffer to 4.5 with AcOH. Neutral ABTS was dissolved in this buffer solution to make a 7 mM ABTS solution. A 2.45 mM persulfate solution was prepared by dissolving K_2_S_2_O_8_ in H_2_O. The ABTS solution and persulfate solution were then added together in a 1:1 ratio. This mixture was then stored in the absence of light at r.t. for 12–16 h. The absorbance of the ABTS radical cation solution was then adjusted with MeOH to 0.700 (± 0.01) at 734 nM. The DPPH radical solution was prepared using the procedure to that described by Brand-Williams et al. by dissolving DPPH in MeOH to make 0.1 mM, which was used for immediately^41^.

#### ABTS and DPPH scavenging method

Reported methods were adapted to measure radical scavenging^39–41^. Briefly, the ABTS radical cation or DPPH radical solution (200 µL) were added to each well in the 96 well plate. The Trolox standard and luteolin samples (10 µL) were then added to the ABTS or DPPH solution. For control, DMSO (10 µL) was added to the well. This was then incubated in the absence of light for 1 h. The resulting absorbance was measured at 734 nm and 517 nm for ABTS and DPPH, respectively, using the PerkinElmer Enspire 2300 Multilabel plate reader. The radical scavenging activity of the standards and samples were measured in triplicate within each experiment. The radical scavenging experiments were conducted three times using freshly prepared radical solutions and samples for each experiment. The percentage radical scavenging activity of Trolox standard against both ABTS and DPPH were calculated according to Eq. (1).

1 $$ \% ~Radical\;~scavenging\;~activity = \frac{{Abs_{{control}}  - Abs_{{sample}} }}{{Abs_{{control}} }}~ \times 100 $$

*Equation 1* Calculating the percentage radical scavenging activity of Trolox standard against ABTS radical cation and DPPH radical solutions.

## Supplementary Information

Supplementary Information.

## Data Availability

General and specific synthetic procedures, characterisation and selected ^1^H and ^13^CNMR spectra of compounds are found in Supplementary Information. Samples of compounds are available from the authors.
