# Supplementary material for: Syntheses of mono-acylated luteolin derivatives, evaluation of their antiproliferative and radical scavenging activities and implications on their oral bioavailability
Source: Sci Rep. 2021 Jun 15;11:12595. doi: 10.1038/s41598-021-92135-w (PMC8206097; doi:10.1038/s41598-021-92135-w)
Supplement: Supplementary file 1 — Supplementary Information. [file 41598_2021_92135_MOESM1_ESM.docx]

**Syntheses of 5-*O*-acyl luteolin derivatives and evaluation of their antiproliferative and radical scavenging activities**

Stephen Lo^a^, Euphemia Leung^b^, Bruno Fedrizzi^a^ and David Barker^a,c*^

*^a^School of Chemical Sciences, University of Auckland, 23 Symonds St, Auckland 1010, New Zealand*

*^b^Auckland Cancer Society Research Centre, University of Auckland, Auckland 1023, New Zealand*

*^c^MacDiarmid Institute for Advanced Materials and Nanotechnology, Wellington 6012, New Zealand*

**Correspondence: d.barker@auckland.ac.nz; Tel.: +64-9-923-9703*

Supplementary Information

Table of Contents

[1. General synthetic procedures 2](#_Toc71381156)

[2. Specific synthetic procedures and characterisation of compounds 3](#_Toc71381157)

[3. Radical scavenging activity data 43](#_Toc71381158)

[4. Supplementary references: 49](#_Toc71381159)

1. General synthetic procedures

General procedure A: Acylation of phenol

To a stirred solution of phenol (1 mmol) in CH_2_Cl_2_ (25 mL) was added Et_3_N (3 mmol) and the mixture was stirred for 5 mins. Acid chloride (0.9 – 3 mmol) was then added and the reaction mixture was stirred for 24 h. The reaction mixture was quenched by addition of saturated aqueous NaHCO_3_. This mixture was extracted with CH_2_Cl_2_ (3 x 20 mL). The combined organic extracts were dried (MgSO_4_) and the solvent removed in vacuo. The crude product was purified by flash chromatography to afford the desired product.

General procedure B: Removal of benzyl or diphenyl dioxol groups

To a stirred solution of benzyl ether (1 mmol) in THF (50 mL) was added 10% Pd/C or 20% Pd(OH)_2_ (0.2 mmol) and the mixture was placed under an atmosphere of H_2_. The reaction mixture was stirred for 24 h or 4 d. This mixture was then filtered through celite, washing with THF. The solvent was removed in vacuo. The crude product was purified by flash chromatography to afford the desired product.

2. Specific synthetic procedures and characterisation of compounds

7-(Benzyloxy)-2-(3ʹ,4-bis(benzyloxy)phenyl)-5-hydroxy-4H-chromen-4-one (2)

To a stirred solution of luteolin (1.00 g, 3.49 mmol) and K_2_CO_3_ (1.93 g, 13.97 mmol) in DMF (70 mL) at r.t. was added benzyl bromide (2.07 mL, 17.47 mmol) and the reaction mixture was stirred at 110 °C for 24 h. The reaction mixture was quenched by addition of H_2_O (100 mL). The resulting mixture was extracted with CH_2_Cl_2_ (3 x 50 mL). The combined organic extracts were further washed with excess H_2_O (3 x 100 mL) to remove DMF. The organic extract was dried (MgSO_4_) and the solvent removed *in vacuo*. The crude product was purified by flash chromatography (19:1 PhMe:Et_2_O) to give the *title compound* **2** (1.46 g, 76%) as a yellow solid.

**R_f_:** 0.87 (3:1 Petroleum ether:EtOAc)

**M.P.:** 175 – 178 °C

**δ_H_** (400 MHz; CDCl_3_): 5.14 (2H, s, 7-*O*CH_2_), 5.24 (2H, s , 3ʹ-*O*CH_2_), 5.25 (2H, s, 4ʹ-*O*CH_2_), 6.44 (1H, d, *J* = 2.4 Hz, 6-H), 6.49 (1H, s, 3-H), 6.52 (1H, d, *J* = 2.4 Hz, 8-H), 7.01 (1H, d, *J* = 8.5 Hz, 5ʹ-H), 7.31 – 7.49 (17H, m, Ar-H, 2ʹ-H and 6ʹ-H), 12.78 (1H, s, 5-OH)

**δ_C_** (100 MHz; CDCl_3_): 70.6 (7-*O*CH_2_), 71.1, 71.7 (3ʹ-*O*CH_2_ and 4ʹ-*O*CH_2_), 93.6 (C-8), 98.9 (C-6), 104.8 (C-3), 105.8 (C-4a), 113.0 (C-2ʹ), 114.2 (C-5ʹ), 120.7 (C-6ʹ), 124.2 (C-1ʹ), 127.3, 127.5, 127.6, 128.2, 128.8, 128.9 (Ar-C), 135.9 (7-*O*CH_2_C(Ar)), 136.5, 136.8 (3ʹ-*O*CH_2_C(Ar) and 4ʹ-*O*CH_2_C(Ar)), 149.0 (C-3ʹ), 152.4 (C-4ʹ), 157.7 (C-8a), 162.4 (C-5), 164.0 (C-2), 164.7 (C-7), 182.5 (C-4)

**IR:** ν_max_/cm^-1^; 667, 691, 705, 733, 746, 766, 801, 808, 834, 852, 866, 909, 842, 972, 1017, 1040, 1080, 1102, 1116, 1139, 1159, 1193, 1218, 1254, 1275, 1304, 1330, 1352, 1366, 1388, 1436, 1454, 1464, 1492, 1516, 1537, 1587, 1607, 1651, 1748, 1877, 2868, 2922, 3037, 3062

**HRMS (ESI^+^):** Found (MNa^+^) 579.1770, C_36_H_28_NaO_6_ requires 579.1778

7-(Benzyloxy)-2-(3ʹ,4ʹ-bis(benzyloxy)phenyl)-4-oxo-4*H*-chromen-5-yl acetate (3a)

The reaction was carried out according to general procedure A with **2** (0.37 g, 0.66 mmol), Et_3_N (0.28 mL, 1.99 mmol) and acetyl chloride (0.12 mL, 1.66 mmol). The crude product was purified by flash chromatography (3:1 Petroleum ether:EtOAc) to give the *title compound* **3a** (0.33 g, 82%) as a white solid.

**R_f_:** 0.28 (3:1 Petroleum ether:EtOAc)

**M.P.:** 190 – 193 °C

**δ_H_** (400 MHz; CDCl_3_): 2.43 (3H, s, 2ʹʹ-H), 5.16 (2H, s, 7-*O*CH_2_), 5.23 (2H, s, 3ʹ-*O*CH_2_), 5.24 (2H, s, 4ʹ-*O*CH_2_), 6.43 (1H, s, 3-H), 6.68 (1H, d, *J* = 2.3 Hz, 6-H), 6.89 (1H, d, *J* = 2.3 Hz, 8-H), 7.00 (1H, d, *J* = 8.4 Hz, 5ʹ-H), 7.31-7.48 (17H, m, Ar-H, 2ʹ-H and 6ʹ-H)

**δ_C_** (100 MHz; CDCl_3_): 21.3 (C-2ʹʹ), 70.9 (7-*O*CH_2_), 71.1, 71.8 (3ʹ-*O*CH_2_ and 4ʹ-*O*CH_2_), 100.1 (C-8), 107.5 (C-3), 108.8 (C-6), 111.4 (C-4a), 113.0 (C-2ʹ), 114.3 (C-5ʹ), 120.5 (C-6ʹ), 124.4 (C-1ʹ), 127.3, 127.5, 127.7, 128.2, 128.7, 128.8, 129.0 (Ar-C), 135.6 (7-*O*CH_2_C(Ar)), 136.6, 136.9 (3ʹ-*O*CH_2_C(Ar) and 4ʹ-*O*CH_2_C(Ar)), 149.0 (C-3ʹ), 150.7 (C-5), 152.1 (C-4ʹ), 158.8 (C-8a), 161.9 (C-2), 162.6 (C-7), 169.9 (C-1ʹʹ), 176.6 (C-4)

**IR:** ν_max_/cm^-1^; 666, 695, 732, 775, 793, 840, 897, 946, 1017, 1086, 1107, 1156, 1197, 1210, 1263, 1277, 1310, 1371, 1432, 1455, 1512, 1602, 1632, 1762, 2929, 3035

**HRMS (ESI^+^):** Found (MNa^+^) 621.1879, C_38_H_30_NaO­_7_ requires 621.1884

**Supplementary Figure 1:** ^1^H and ^13^C NMR of **3a**


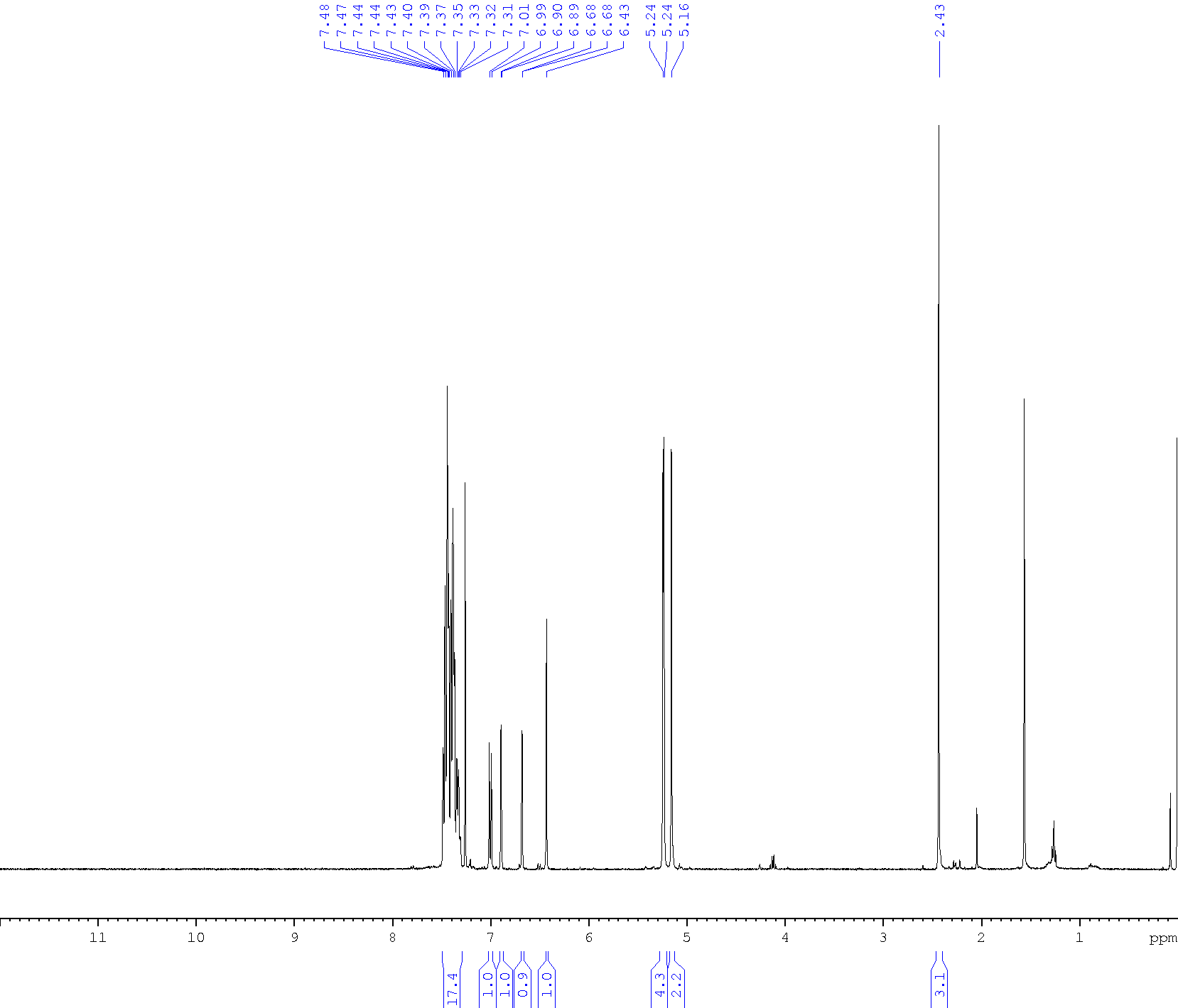


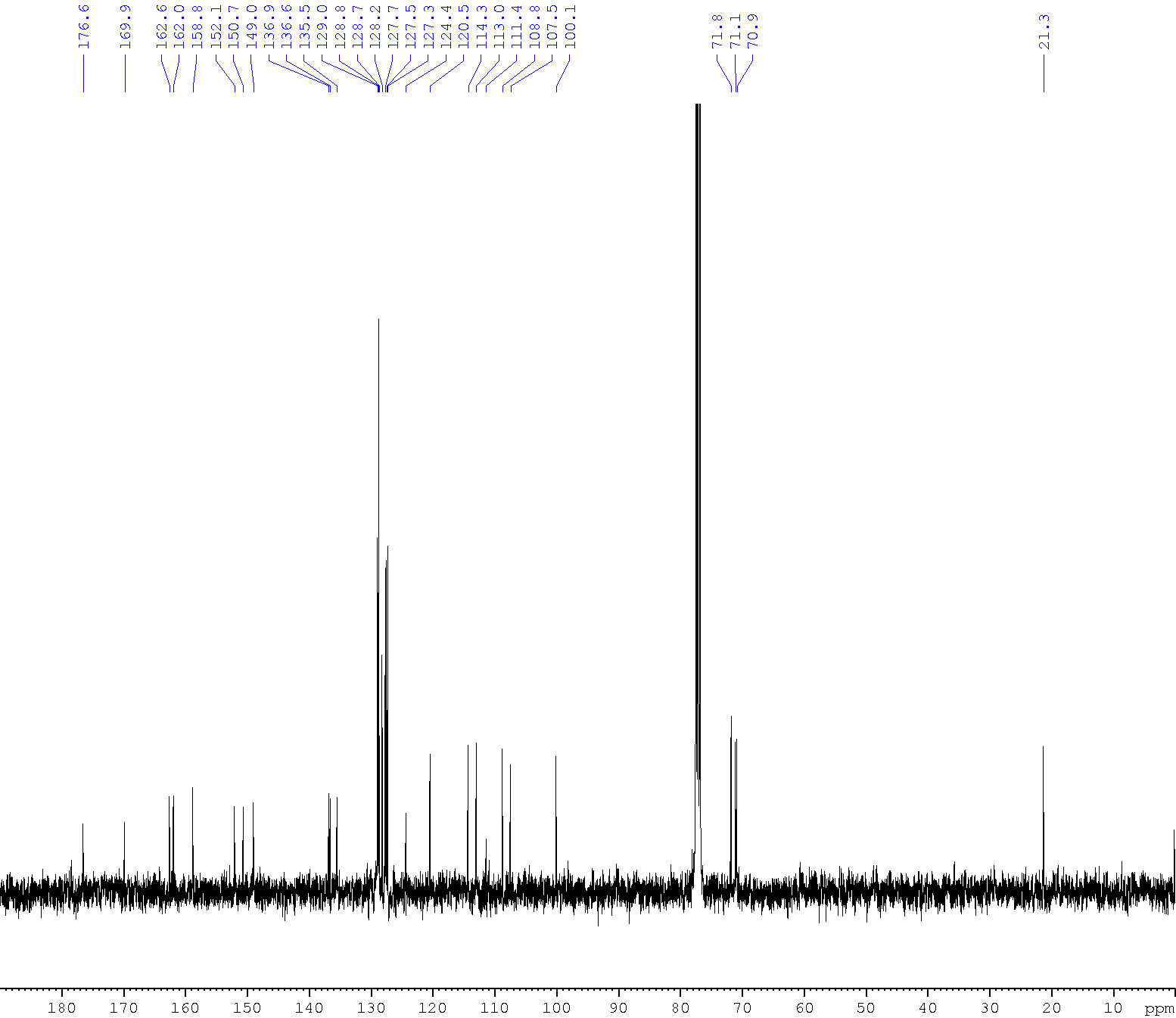


2-(3ʹ,4ʹ-Dihydroxyphenyl)-7-hydroxy-4-oxo-4*H*-chromen-5-yl acetate (4a)

The reaction was carried out according to general procedure B **3a** (0.33 g, 0.54 mmol) and 20% Pd(OH)_2_/C (76 mg, 0.11 mmol). The reaction was stirred for 24 h. The crude product was purified by flash chromatography (1:3 Petroleum ether:EtOAC) to give the *title compound* **4a** (0.15 g, 86%) as a yellow solid.

**R_f_:** 0.29 (1:3 Petroleum ether:EtOAC)

**M.P.:** 167 – 170 °C

**δ_H_** (400 MHz; d_6_-DMSO): 2.28 (3H, s, 2ʹʹ-H), 6.45 (1H, s, 3-H), 6.53 (1H, d, *J* = 2.0 Hz, 6-H), 6.86 (1H, d, *J* = 2.0 MHz, 8-H), 6.88 (1H, d, *J* = 8.9 Hz, 5ʹ-H), 7.34-7.37 (2H, m, 2ʹ-H and 6ʹ-H)

**δ_C_** (100 MHz; d_6_-DMSO): 21.0 (C-2ʹʹ), 100.7 (C-8), 105.3 (C-3), 108.5 (C-6), 109.3 (C-4a), 113.1 (C-2ʹ), 116.0 (C-5ʹ), 118.5 (C-6ʹ), 121.6 (C-1ʹ), 145.7 (C-3ʹ), 149.2 (C-4ʹ), 150.1 (C-5), 158.1 (C-8a), 161.5 (C-2), 162.0 (C-7), 168.8 (C-1ʹʹ), 175.1 (C-4)

**IR:** ν_max_/cm^-1^; 668, 684, 725, 739, 774, 789, 834, 857, 913, 934, 951, 998, 1034, 1083, 1114, 1189, 1231, 1271, 1367, 1449, 1505, 1523, 1567, 1591, 1628, 1726, 2168, 2614, 2945, 3323

**HRMS (ESI^+^):** Found (MNa^+^) 351.0473, C_17_H_12_NaO­_7_ requires 351.0475

**Supplementary Figure 2:** ^1^H and ^13^C NMR of **4a**


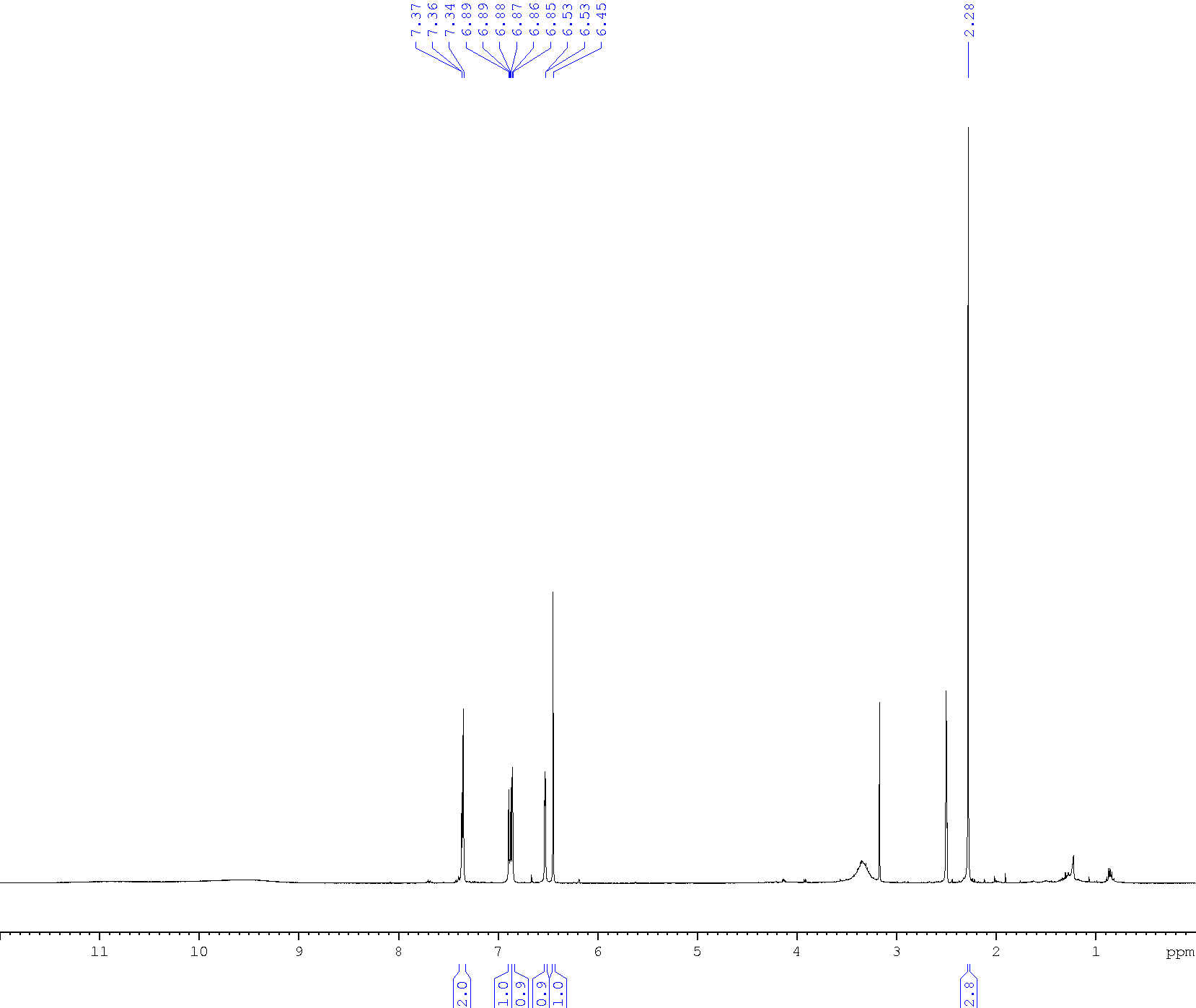


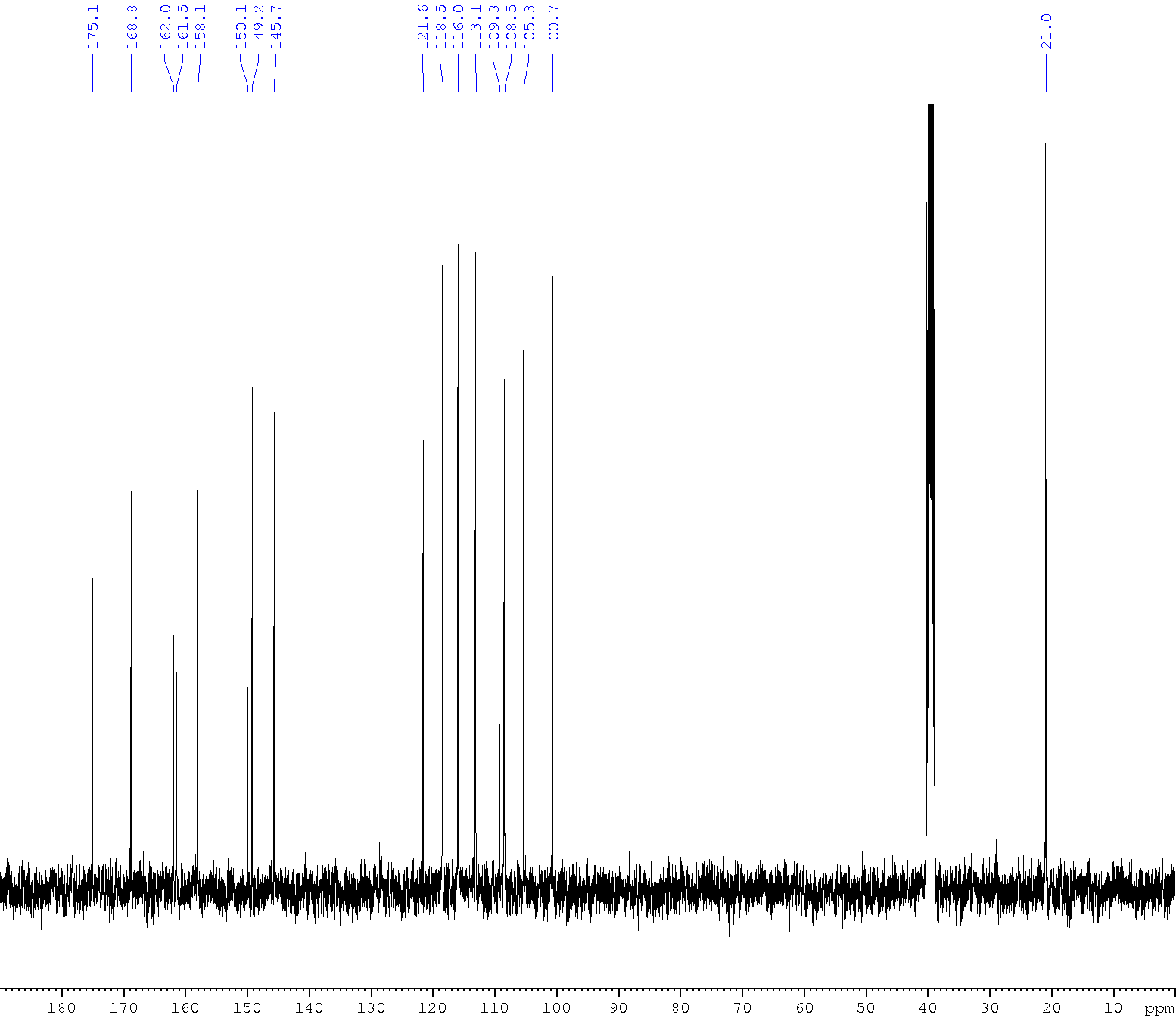


7-(Benzyloxy)-2-(3ʹ,4ʹ-bis(benzyloxy)phenyl)-4-oxo-4*H*-chromen-5-yl propionate (3b)

The reaction was carried out according to general procedure A with **2** (0.22 g, 0.40 mmol), Et_3_N (0.16 mL, 1.19 mmol) and propionyl chloride (0.07 mL, 0.79 mmol). The crude product was purified by flash chromatography (4:1 Petroleum ether:EtOAC) to give the *title compound* **3b** (0.17 g, 68%) as a white solid.

**R_f_:** 0.58 (3:1 Petroleum ether:EtOAC)

**M.P.:** 149 – 151 °C

**δ_H_** (400 MHz; CDCl_3_): 1.31 (3H, t, *J* = 7.6 Hz, 3ʹʹ-H), 2.77 (2H, q, *J* = 7.6 Hz, 2ʹʹ-H), 5.16 (2H, s, 7-*O*CH_2_), 5.23 (2H, s, 3ʹ-*O*CH_2_), 5.24 (2H, s, 4ʹ-*O*CH_2_), 6.43 (1H, s, 3-H), 6.68 (1H, d, *J* = 2.5 Hz, 6-H), 6.89 (1H, d, *J* = 2.5 Hz, 8-H), 7.00 (1H, d, *J* = 8.5 Hz, 5ʹ-H), 7.32-7.48 (17H, m, Ar-H, 2ʹ-H and 6ʹ-H)

**δ_C_** (100 MHz; CDCl_3_): 8.9 (C-3ʹʹ), 27.8 (C-2ʹʹ), 70.9 (7-*O*CH_2_), 71.1, 71.8 (3ʹ-*O*CH_2_ and 4ʹ-*O*CH_2_), 100.1 (C-8), 107.5 (C-3), 108.8 (C-6), 111.6 (C-4a), 113.0 (C-2ʹ), 114.3 (C-5ʹ), 120.5 (C-6ʹ), 124.4 (C-1ʹ), 127.3, 127.5, 127.6, 127.7, 128.2, 128.7, 128.8, 129.0 (Ar-C), 135.6 (7-*O*CH_2_C(Ar)), 136.6, 136.9 (3ʹ-*O*CH_2_C(Ar) and 4ʹ-*O*CH_2_C(Ar)), 149.0 (C-3ʹ), 150.9 (C-5), 152.1 (C-4ʹ), 158.8 (C-8a), 161.9 (C-2), 162.6 (C-7), 173.2 (C-1ʹʹ), 176.6 (C-4)

**IR:** ν_max_/cm^-1^; 561, 579, 592, 616, 639, 695, 731, 761, 771, 795, 836, 859, 893, 911, 941, 1012, 1056, 1091, 1105, 1136, 1155, 1186, 1203, 1263, 1314, 1351, 1372, 1419, 1432, 1455, 1516, 1607, 1631, 1643, 1754, 2213, 2943, 3035

**HRMS (ESI^+^):** Found (MNa^+^) 635.2019, C_39_H_32_NaO_7_ requires 635.2040

**Supplementary Figure 3:** ^1^H and ^13^C NMR of **3b**


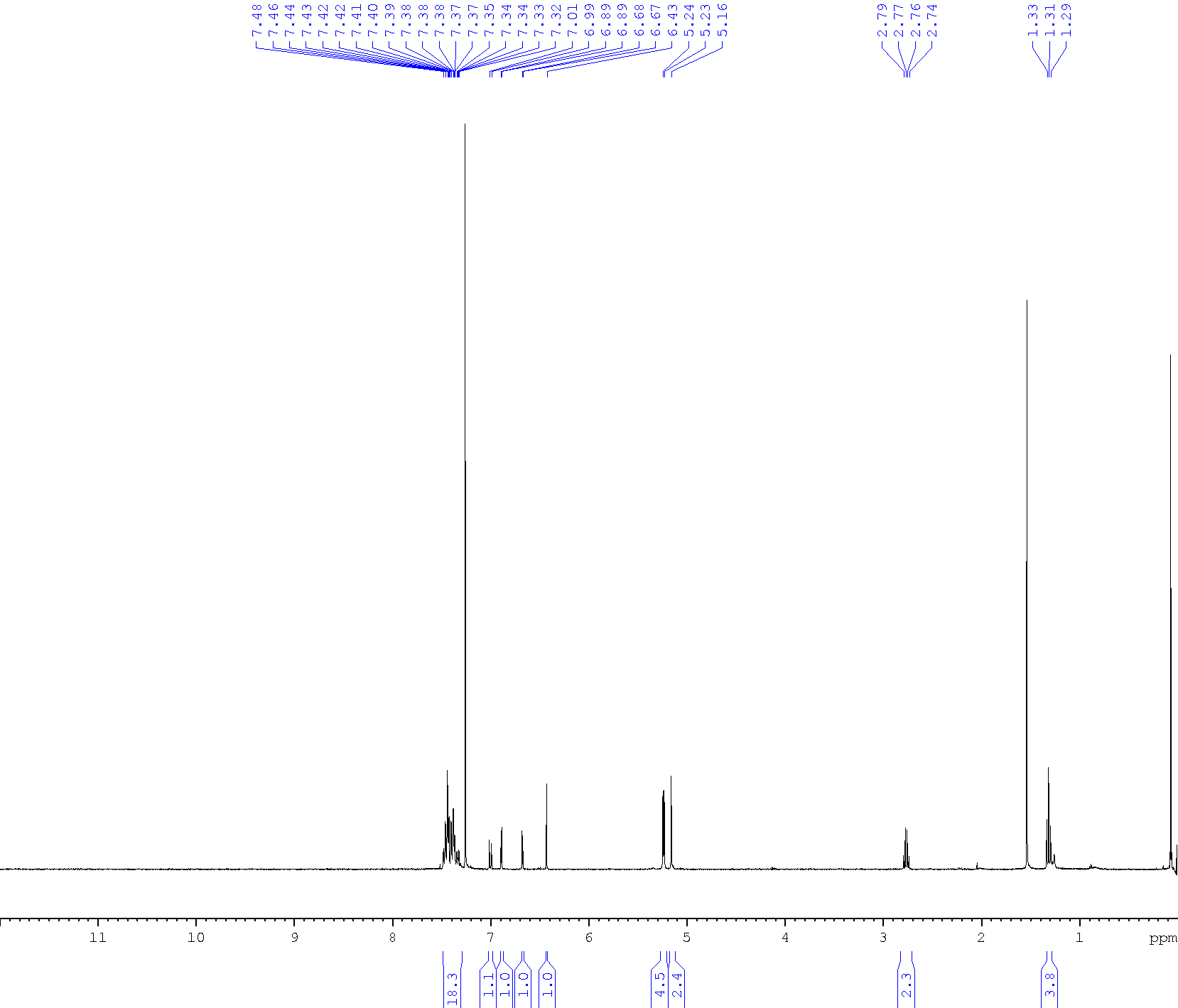


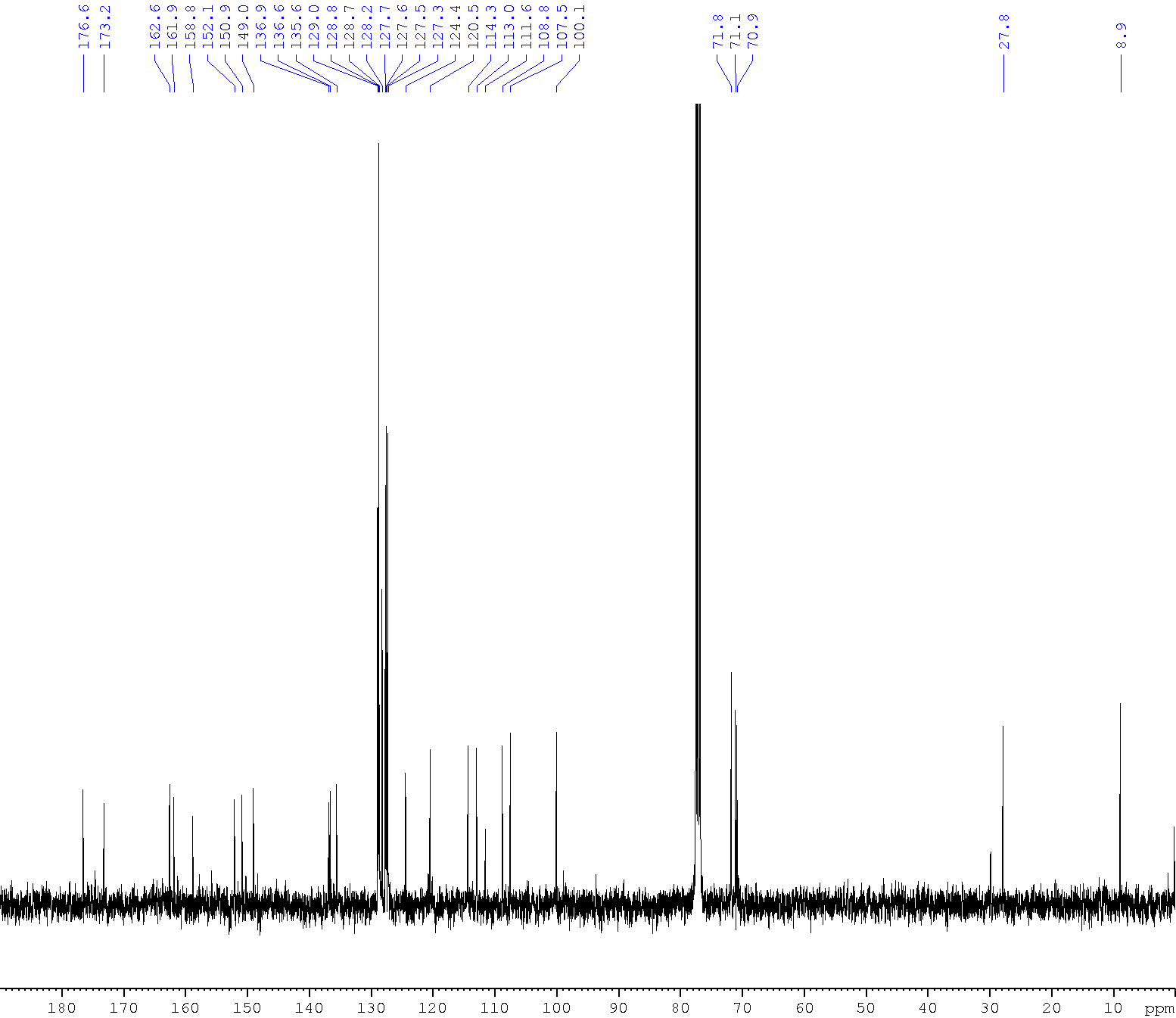


2-(3ʹ,4ʹ-Dihydroxyphenyl)-7-hydroxy-4-oxo-4*H*-chromen-5-yl propionate (4b)

The reaction was carried out according to general procedure B with **3b** (0.17 g, 0.27 mmol) and 20% Pd(OH)_2_/C (38 mg, 0.05 mmol). The reaction was stirred for 24 h. The crude product was purified by flash chromatography (1:2 Petroleum ether:EtOAC) to give the *title compound* **4b** (90 mg, 97%) as a yellow solid.

**R_f_:** 0.43 (1:3 Petroleum ether:EtOAC)

**M.P.:** 195 - 197 °C

**δ_H_** (400 MHz; d_6_-DMSO): 1.15 (3H, t, *J* = 7.6 Hz, 3ʹʹ-H), 2.63 (2H, q, *J* = 7.6 Hz, 2ʹʹ-H), 6.45 (1H, s, 3-H), 6.52 (1H, d, *J* = 2.5 Hz, 6-H), 6.85 (1H, d, *J* = 2.5 Hz, 8-H), 6.88 (1H, d, *J* = 9.0 Hz, 5ʹ-H), 7.34-7.37 (2H, m, 2ʹ-H and 6ʹ-H), 9.36 (1H, s, 4ʹ-OH), 9.80 (1H, s, 3ʹ-OH), 11.03 (1H, s, 7-OH)

**δ_C_** (100 MHz; d_6_-DMSO): 8.6 (C-3ʹʹ), 26.9 (C-2ʹʹ), 100.6 (C-8), 105.3 (C-3), 108.4 (C-6), 109.3 (C-4a), 113.1 (C-2ʹ), 116.0 (C-5ʹ), 118.5 (C-6ʹ), 121.6 (C-1ʹ), 145.7 (C-3ʹ), 149.2 (C-4ʹ), 150.2 (C-5), 158.1 (C-8a), 161.5 (C-2), 162.0 (C-7), 172.1 (C-1ʹʹ), 175.1 (C-4)

**IR:** ν_max_/cm^-1^; 684, 773, 790, 810, 837, 905, 945, 997, 1028, 1087, 1113, 1158, 1264, 1355, 1447, 1498, 1516, 1540, 1601, 1723, 2608, 2926, 3088, 3320

**HRMS (ESI^+^):** Found (MNa^+^) 365.0622, C_18_H_14_NaO_7_ requires 365.0632

The ^1^H NMR δ values are in agreement with literature.^1^

**Supplementary Figure 4:** ^1^H and ^13^C NMR of **4b**


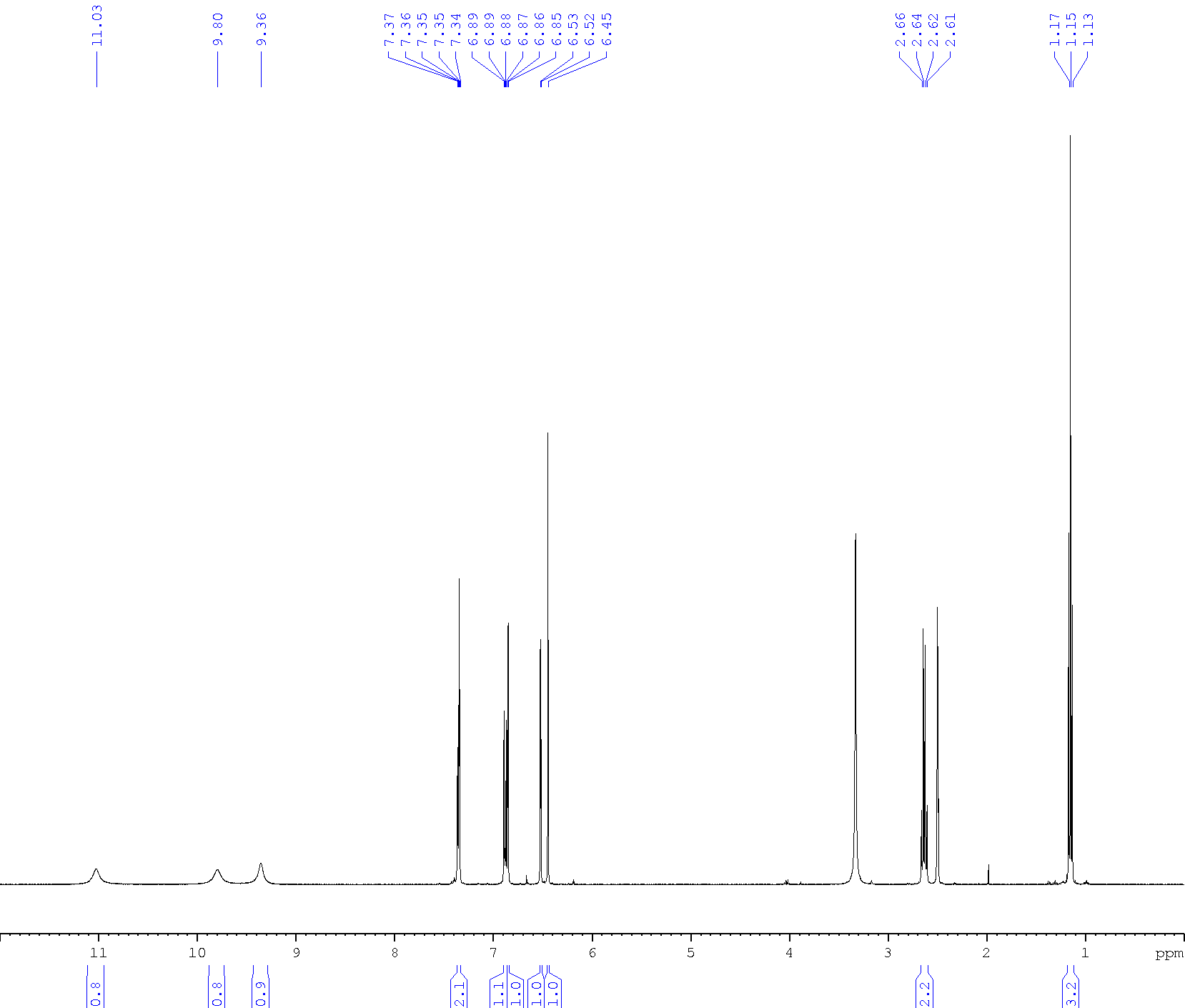


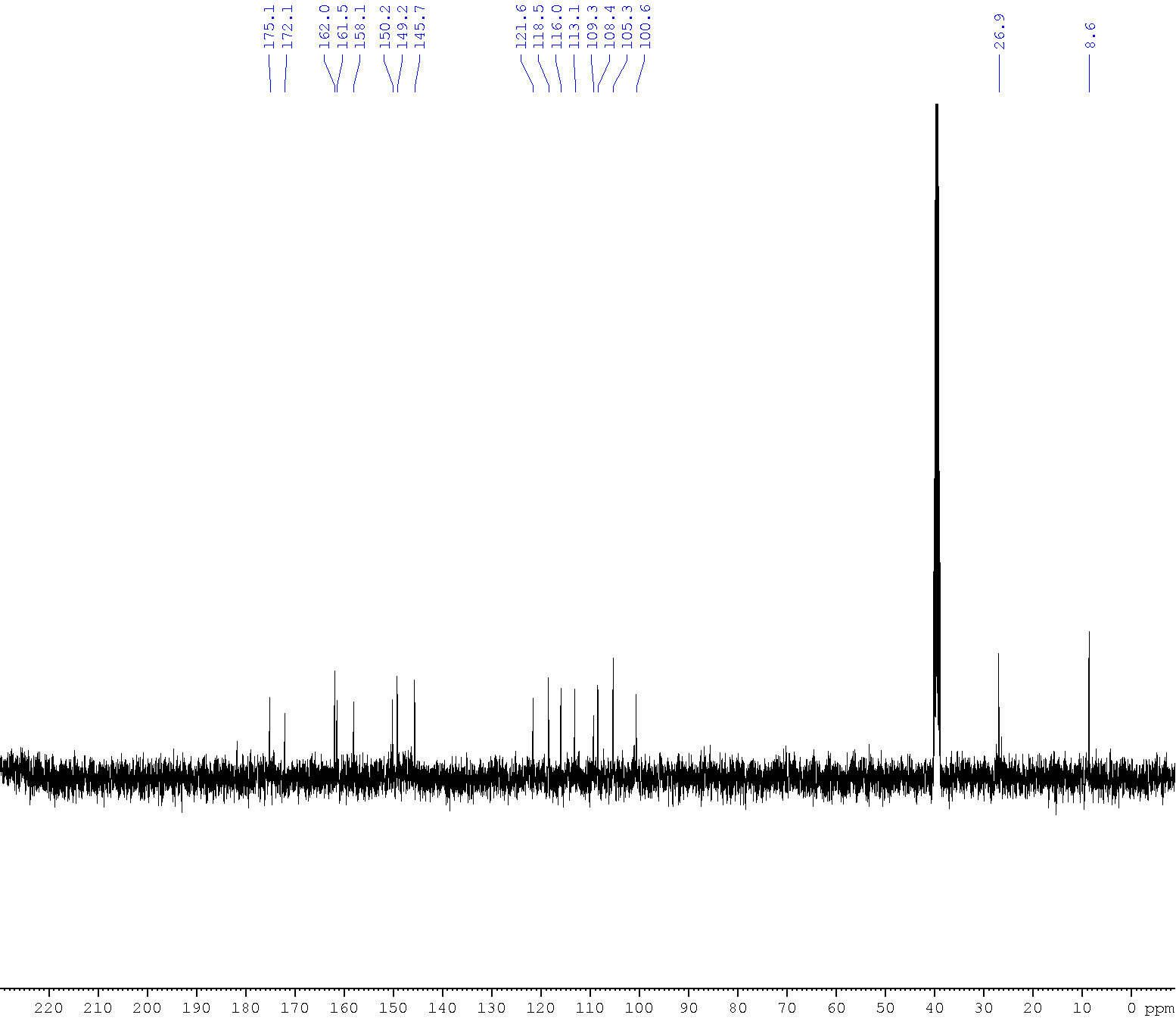


7-(Benzyloxy)-2-(3ʹ,4ʹ-bis(benzyloxy)phenyl)-4-oxo-4*H*-chromen-5-yl hexanoate (3c)

The reaction was carried out according to general procedure A with **2** (0.11 g, 0.19 mmol), Et_3_N (0.08 mL, 0.57 mmol) and hexanoyl chloride (0.05 mL, 0.38 mmol). The crude product was purified by flash chromatography (4:1 Petroleum ether:EtOAC) to give the *title compound* **3c** (86 mg, 70%) as a yellow solid.

**R_f_:** 0.76 (3:1 Petroleum ether:EtOAC)

**M.P.:** 120 – 122 °C

**δ_H_** (400 MHz; CDCl_3_): 0.94 (3H, t, *J* = 7.0 Hz, 6ʹʹ-H), 1.37 – 1.46 (4H, m, 4ʹʹ-H and 5ʺ-H), 1.82 (2H, p, *J* = 7.5 Hz, 3ʹʹ-H), 2.73 (2H, t, *J* = 7.5 Hz, 2ʹʹ-H), 5.15 (2H, s, 7-*O*CH_2_), 5.23 (2H, s, 3ʹ-*O*CH_2_), 5.24 (2H, s, 4ʹ-*O*CH_2_), 6.44 (1H, s, 3-H), 6.67 (1H, d, *J* = 2.6 Hz, 6-H), 6.89 (1H, d, *J* = 2.6 Hz, 8-H), 7.00 (1H, d, *J* = 8.9 Hz, 5ʹ-H), 7.30-7.49 (17H, m, Ar-H, 2ʹ-H and 6ʹ-H)

**δ_C_** (100 MHz; CDCl_3_): 14.1 (C-6ʹʹ), 22.5 (C-5ʹʹ), 24.3 (C-3ʹʹ), 31.5 (C-4ʹʹ), 34.4 (C-2ʹʹ), 70.9 (7-*O*CH_2_), 71.1, 71.8 (3ʹ-*O*CH_2_ and 4ʹ-*O*CH_2_), 100.0 (C-8), 107.5 (C-3), 108.8 (C-6), 111.6 (C-4a), 113.0 (C-2ʹ), 114.3 (C-5ʹ), 120.5 (C-6ʹ), 124.4 (C-1ʹ), 127.3, 127.5, 127.7, 128.2, 128.7, 128.8, 128.9 (Ar-C), 135.6 (7-*O*CH_2_C(Ar)), 136.6, 136.9 (3ʹ-*O*CH_2_C(Ar) and 4ʹ-*O*CH_2_C(Ar)), 149.0 (C-3ʹ), 150.9 (C-5), 152.1 (C-4ʹ), 158.8 (C-8a), 161.9 (C-2), 162.6 (C-7), 172.5 (C-1ʹʹ), 176.6 (C-4)

**IR:** ν_max_/cm^-1^; 664, 676, 694, 730, 796, 800, 830, 848, 859, 896, 908, 948, 1017, 1026, 1037, 1105, 1134, 1144, 1160, 1192, 1213, 1251, 1265, 1275, 1289, 1317, 1349, 1431, 1455, 1499, 1516, 1610, 1632, 1646, 1752, 2867, 2929, 3035

**HRMS (ESI^+^):** Found (MNa^+^) 677.248, C_42_H_38_NaO­_7_ requires 677.2510

**Supplementary Figure 5:** ^1^H and ^13^C NMR of **3c**


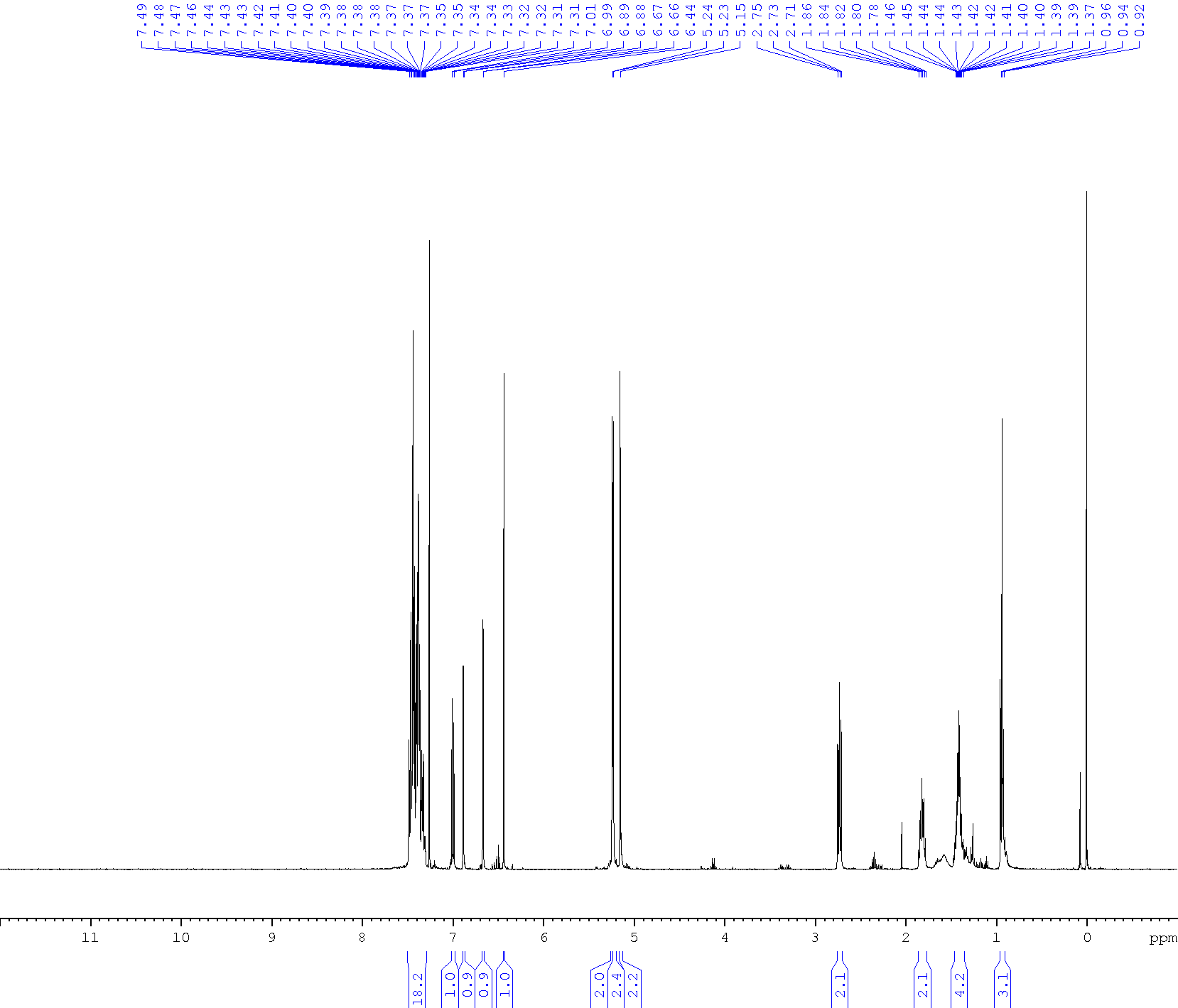


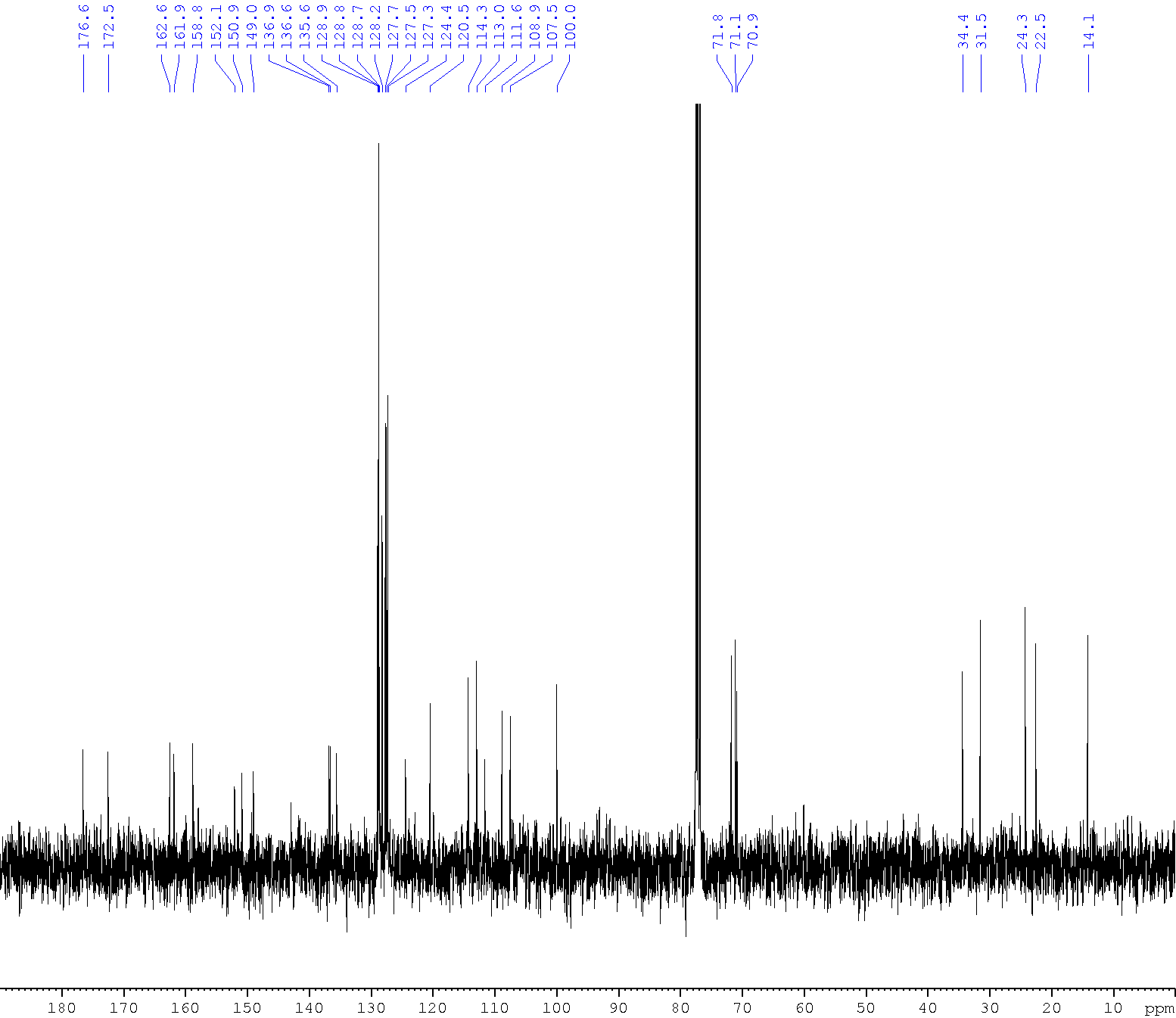


2-(3ʹ,4ʹ-Dihydroxyphenyl)-7-hydroxy-4-oxo-4*H*-chromen-5-yl hexanoate (4c)

The reaction was carried out according to general procedure B with **3c** (0.15 g, 0.23 mmol) and 10% Pd/C (49 mg, 0.05 mmol). The reaction was stirred for 24 h. The crude product was purified by flash chromatography (1:1 Petroleum ether:EtOAC) to give the *title compound* **4c** (80 mg, 91%) as a yellow solid.

**R_f_:** 0.57 (1:3 Petroleum ether:EtOAC)

**M.P.:** 195 – 198 °C

**δ_H_** (400 MHz; d_6_-DMSO): 0.90 (3H, t, *J* = 7.1 Hz, 6ʹʹ-H), 1.29 – 1.40 (4H, m, 4ʹʹ-H and 5ʹʹ-H), 1.66 (2H, p, *J* = 7.4 Hz, 3ʹʹ-H), 2.61 (2H, t, *J* = 7.4 Hz, 2ʹʹ-H), 6.45 (1H, s, 3-H), 6.51 (1H, d, *J* = 2.2 Hz, 6-H), 6.85 (1H, d, *J* = 2.2 Hz, 8-H), 6.88 (1H, d, *J* = 8.8 Hz, 5ʹ-H), 7.34-7.37 (2H, m, 2ʹ-H and 6ʹ-H), 9.35 (1H, s, 4ʹ-OH), 9.80 (1H, s, 3ʹ-OH), 11.02 (1H, s, 7-OH)

**δ_C_** (100 MHz; d_6_-DMSO): 13.8 (C-6ʹʹ), 21.8 (C-5ʹʹ), 23.6 (C-3ʹʹ), 30.7 (C-4ʹʹ), 33.4 (C-2ʹʹ) 100.7 (C-8), 105.3 (C-3), 108.4 (C-6), 109.4 (C-4a), 113.1 (C-2ʹ), 116.0 (C-5ʹ), 118.5 (C-6ʹ), 121.6 (C-1ʹ), 145.7 (C-3ʹ), 149.2 (C-4ʹ), 150.1 (C-5), 158.1 (C-8a), 161.5 (C-2), 161.9 (C-7), 171.3 (C-1ʹʹ), 175.1 (C-4)

**IR:** ν_max_/cm^-1^; 684, 731, 789, 821, 837, 847, 946, 998, 1021, 1081, 1113, 1160, 1181, 1209, 1263, 1296, 1313, 1391, 1443, 1505, 1524, 1589, 1606, 1634, 1720, 2036, 2873, 2931, 3269, 3532

**HRMS (ESI^+^):** Found (MNa^+^) 407.1094, C_21_H_20_NaO­_7_ requires 407.1101

**Supplementary Figure 6:** ^1^H and ^13^C NMR of **4c**


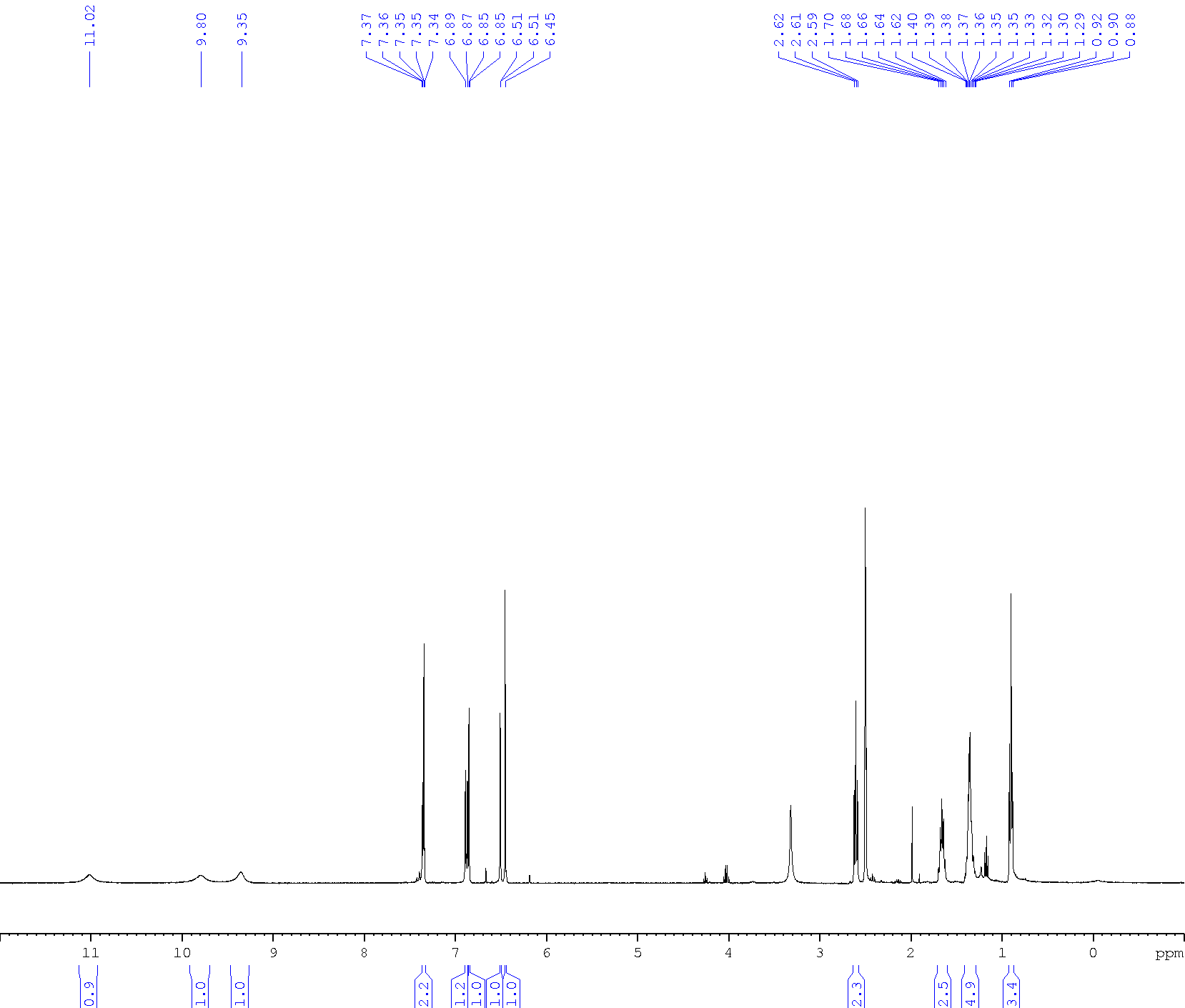


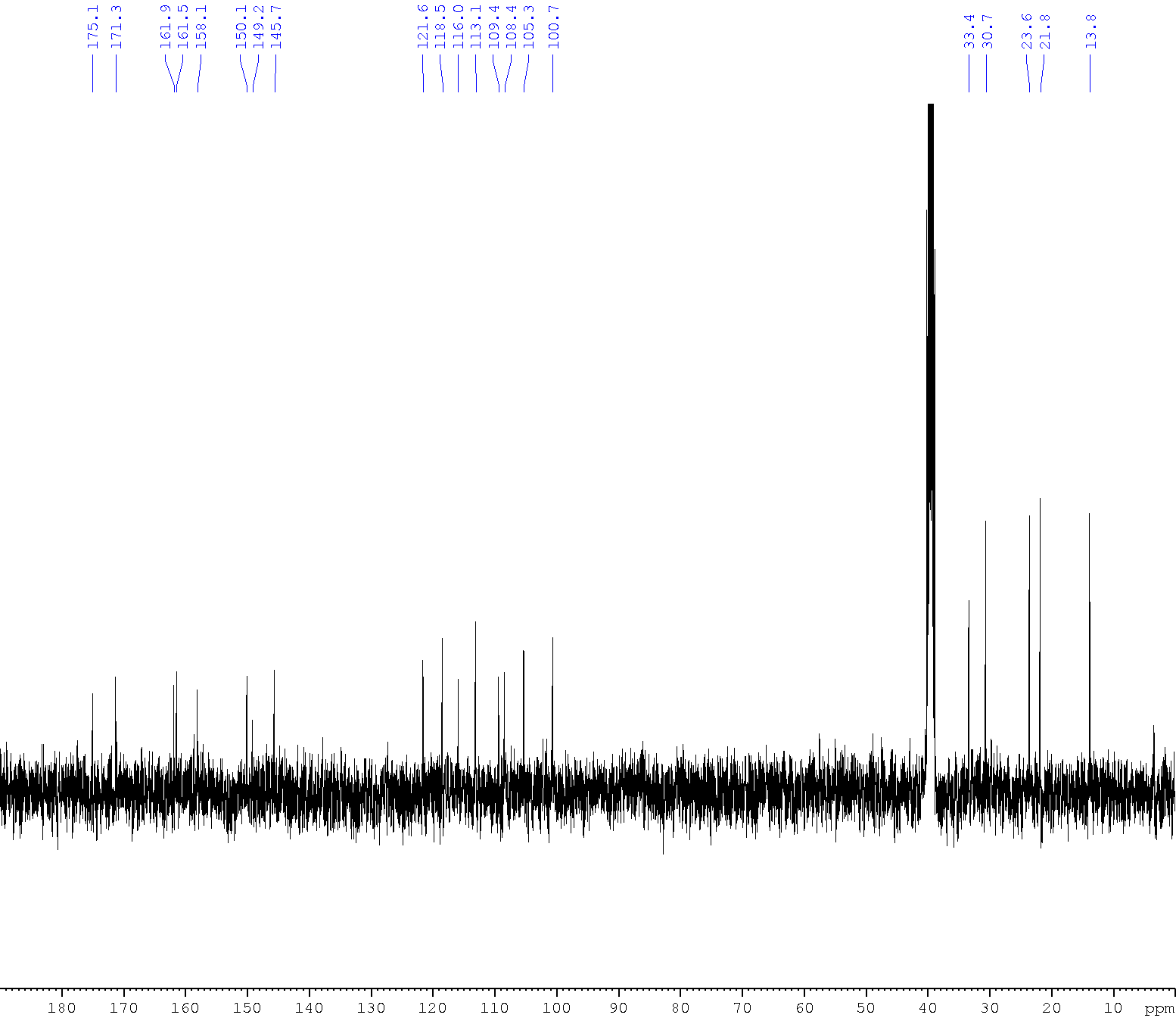


7-(Benzyloxy)-2-(3ʹ,4ʹ-bis(benzyloxy)phenyl)-4-oxo-4*H*-chromen-5-yl octanoate (3d)

The reaction was carried out according to general procedure A with **2** (0.25 g, 0.45 mmol), Et_3_N (0.19 mL, 1.36 mmol) and octanoyl chloride (0.16 mL, 0.91 mmol). The crude product was purified by flash chromatography (4:1 Petroleum ether:EtOAC) to give the *title compound* **3d** (0.17 g, 55%) as a white solid.

**R_f_:** 0.60 (4:1 Petroleum ether:EtOAC)

**M.P.:** 105 – 108 °C

**δ_H_** (400 MHz; CDCl_3_): 0.89 (3H, t, *J* = 7.0 Hz, 8ʹʹ-H), 1.29 – 1.46 (8H, broad m, 4ʹʹ-H, 5ʹʹ-H, 6ʹʹ-H and 7ʹʹ-H), 1.81 (2H, p, *J* = 7.6 Hz, 3ʹʹ-H), 2.73 (2H, t, *J* = 7.6 Hz, 2ʹʹ-H), 5.16 (2H, s, 7-*O*CH_2_), 5.23 (2H, s, 3ʹ-*O*CH_2_), 5.24 (2H, s, 4ʹ-*O*CH_2_), 6.43 (1H, s, 3-H), 6.68 (1H, d, *J* = 2.5 Hz, 6-H), 6.89 (1H, d, *J* = 2.5 Hz, 8-H), 7.00 (1H, d, *J* = 8.5 Hz, 5ʹ-H), 7.30-7.49 (17H, m, Ar-H, 2ʹ-H and 6ʹ-H)

**δ_C_** (100 MHz; CDCl_3_): 14.2 (C-8ʹʹ), 22.8 (C-7ʹʹ), 24.6 (C-3ʹʹ), 29.1, 29.3 (C-4ʹʹ and C-5ʹʹ), 31.8 (C-6ʹʹ), 34.4 (C-2ʹʹ), 70.8 (7-*O*CH_2_), 71.1, 71.7 (3ʹ-*O*CH_2_ and 4ʹ-*O*CH_2_), 100.0 (C-8), 107.4 (C-3), 108.8 (C-6), 111.5 (C-4a), 112.9 (C-2ʹ), 114.3 (C-5ʹ), 120.4 (C-6ʹ), 124.3 (C-1ʹ), 127.3, 127.5, 127.7, 128.2, 128.6, 128.7, 128.8, 128.9 (Ar-C), 135.6 (7-*O*CH_2_C(Ar)), 136.6, 136.9 (3ʹ-*O*CH_2_C(Ar) and 4ʹ-*O*CH_2_C(Ar)), 149.0 (C-3ʹ), 150.8 (C-5), 152.0 (C-4ʹ), 158.8 (C-8a), 161.9 (C-2), 162.5 (C-7), 172.5 (C-1ʹʹ), 176.6 (C-4)

**IR:** ν_max_/cm^-1^; 661, 676, 694, 730, 748, 777, 794, 810, 856, 920, 951, 979, 990, 1006, 1019, 1104, 1137, 1161, 1245, 1260, 1275, 1293, 1318, 1347, 1420, 1432, 1456, 1515, 1598, 1607, 1629, 1646, 1718, 1757, 2853, 2920, 3064

**HRMS (ESI^+^):** Found (MNa^+^) 705.2800, C_44_H_42_NaO­_7_ requires 705.2823

**Supplementary Figure 7:** ^1^H and ^13^C NMR of **3d**


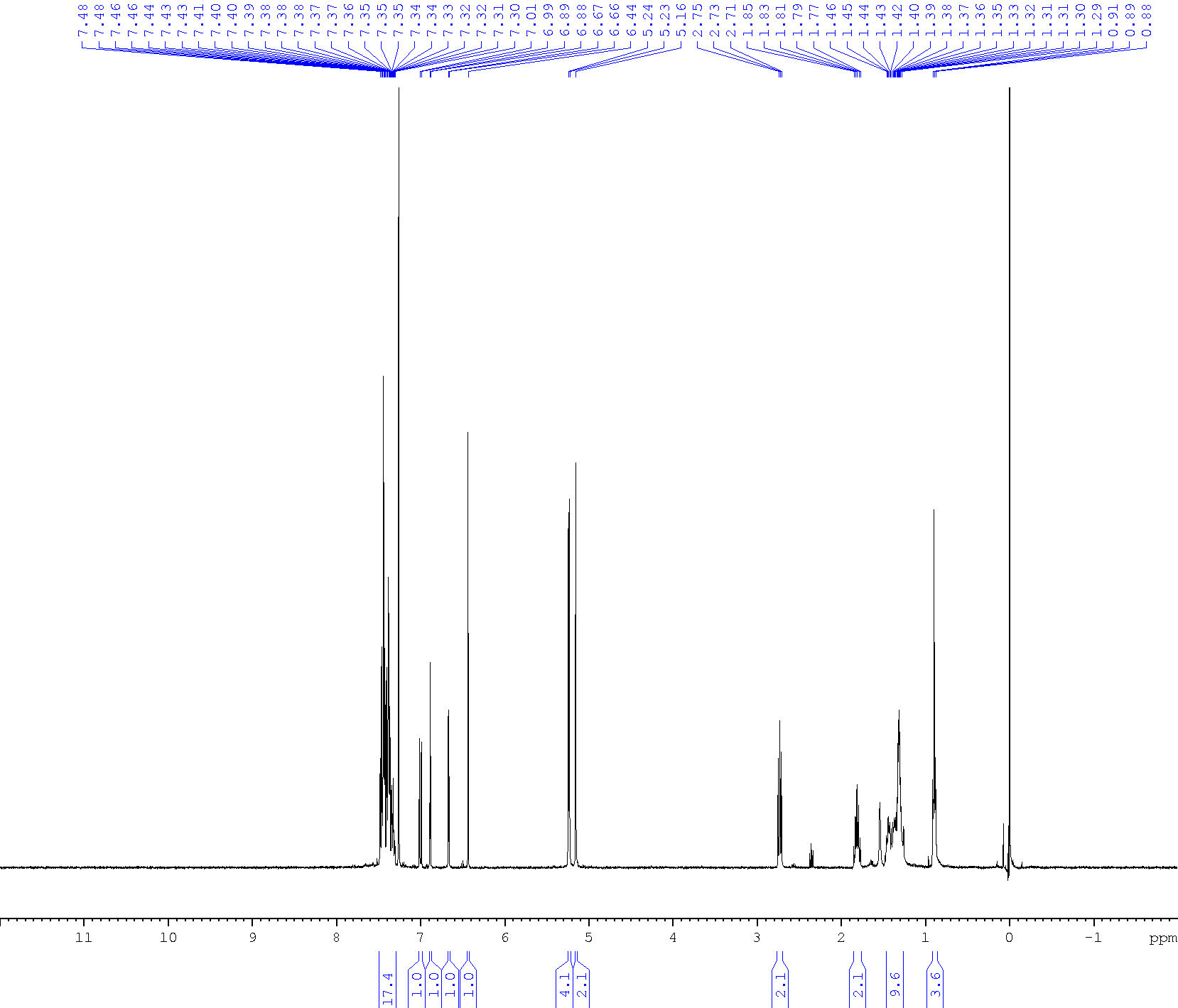


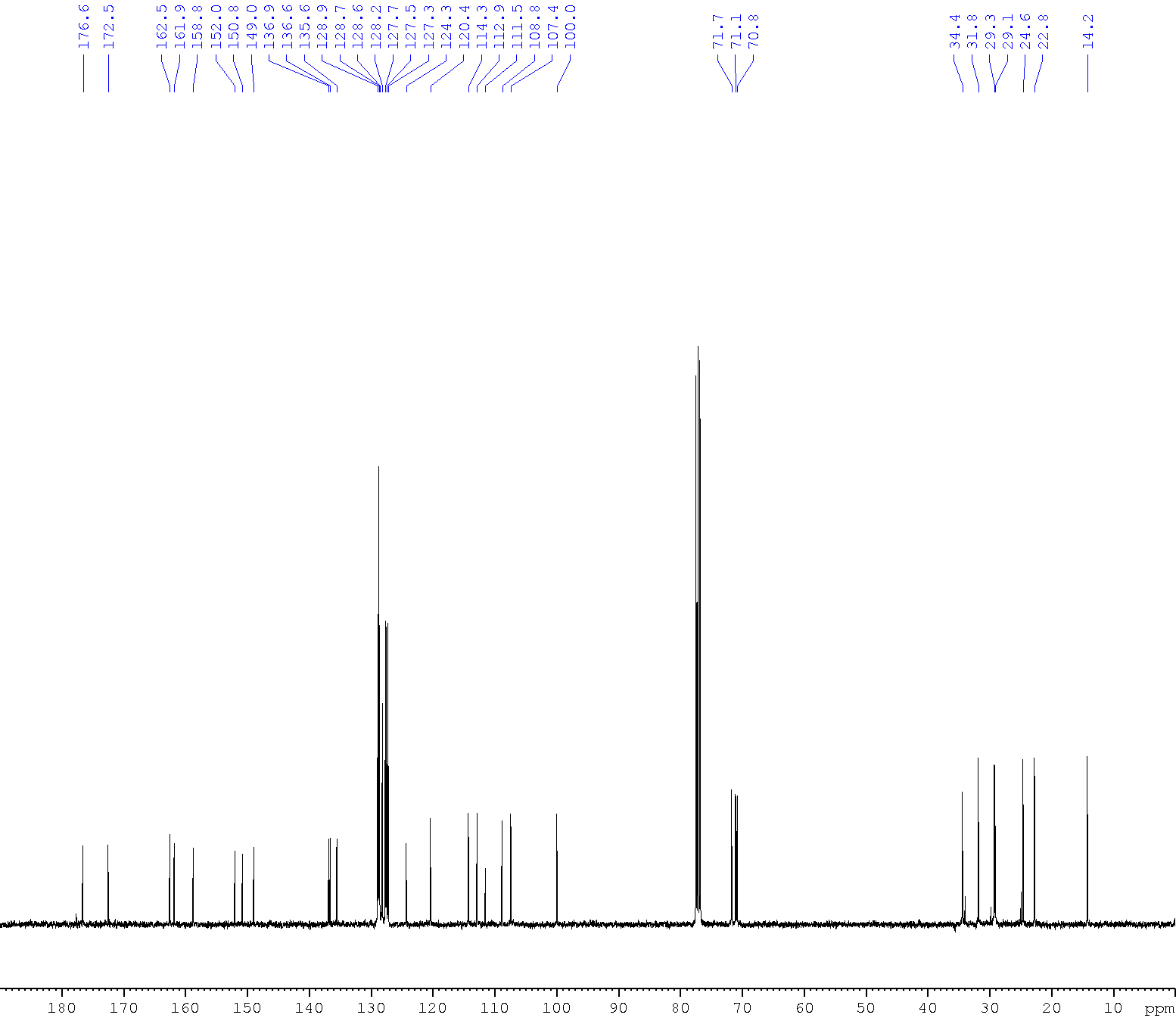


2-(3ʹ,4ʹ-Dihydroxyphenyl)-7-hydroxy-4-oxo-4*H*-chromen-5-yl octanoate (4d)

The reaction was carried out according to general procedure B with **3d** (0.17 g, 0.25 mmol) and 20% Pd(OH)_2_/C (35 mg, 0.05 mmol). The reaction was stirred for 24 h. The product was purified by flash chromatography (1:1 Petroleum ether:EtOAC) to give the *title compound* **4d** (87 mg, 85%) as a white solid.

**R_f_:** 0.39 (1:2 Petroleum ether:EtOAC)

**M.P.:** 177 – 180 °C

**δ_H_** (400 MHz; d_6_-DMSO): 0.89 (3H, t, *J* = 6.9 Hz, 8ʹʹ-H), 1.22 – 1.39 (8H, broad m, 4ʹʹ-H, 5ʹʹ-H, 6ʹʹ-H and 7ʹʹ-H), 1.65 (2H, p, *J*  = 7.4 Hz, 3ʹʹ-H), 2.60 (2H, t, *J*  = 7.4 Hz, 2ʹʹ-H), 6.45 (1H, s, 3-H), 6.50 (1H, d, *J* = 2.4 Hz, 6-H), 6.85 (1H, d, *J* = 2.4 Hz, 8-H), 6.88 (1H, d, *J* = 8.8 Hz, 5ʹ-H), 7.35-7.37 (2H, m, 2ʹ-H and 6ʹ-H)

**δ_C_** (100 MHz; d_6_-DMSO): 13.9 (C-8ʹʹ), 22.1 (C-7ʹʹ), 23.9 (C-3ʹʹ), 28.7, 29.0 (C-4ʹʹ and C-5ʹʹ), 31.3 (C-6ʹʹ), 33.4 (C-2ʹʹ), 100.7 (C-8), 105.3 (C-3), 108.5 (C-6), 109.3 (C-4a), 113.1 (C-2ʹ), 116.0 (C-5ʹ), 118.5 (C-6ʹ), 121.6 (C-1ʹ), 145.7 (C-3ʹ), 149.2 (C-4ʹ), 150.1 (C-5), 158.1 (C-8a), 161.5 (C-2), 162.1 (C-7), 171.3 (C-1ʹʹ), 175.1 (C-4)

**IR:** ν_max_/cm^-1^; 666, 683, 728, 780, 814, 844, 954, 980, 1004, 1111, 1134, 1158, 1216, 1264, 1297, 1326, 1354, 1385, 1436, 1498, 1514, 1600, 1632, 1723, 2444, 2606, 2857, 2929, 3267

**HRMS (ESI^+^):** Found (MNa^+^) 435.1402, C_23_H_24_NaO­_7_ requires 435.1414

The ^1^H NMR values are in agreement with literature.^1^

**Supplementary Figure 8:** ^1^H and ^13^C NMR of **4d**


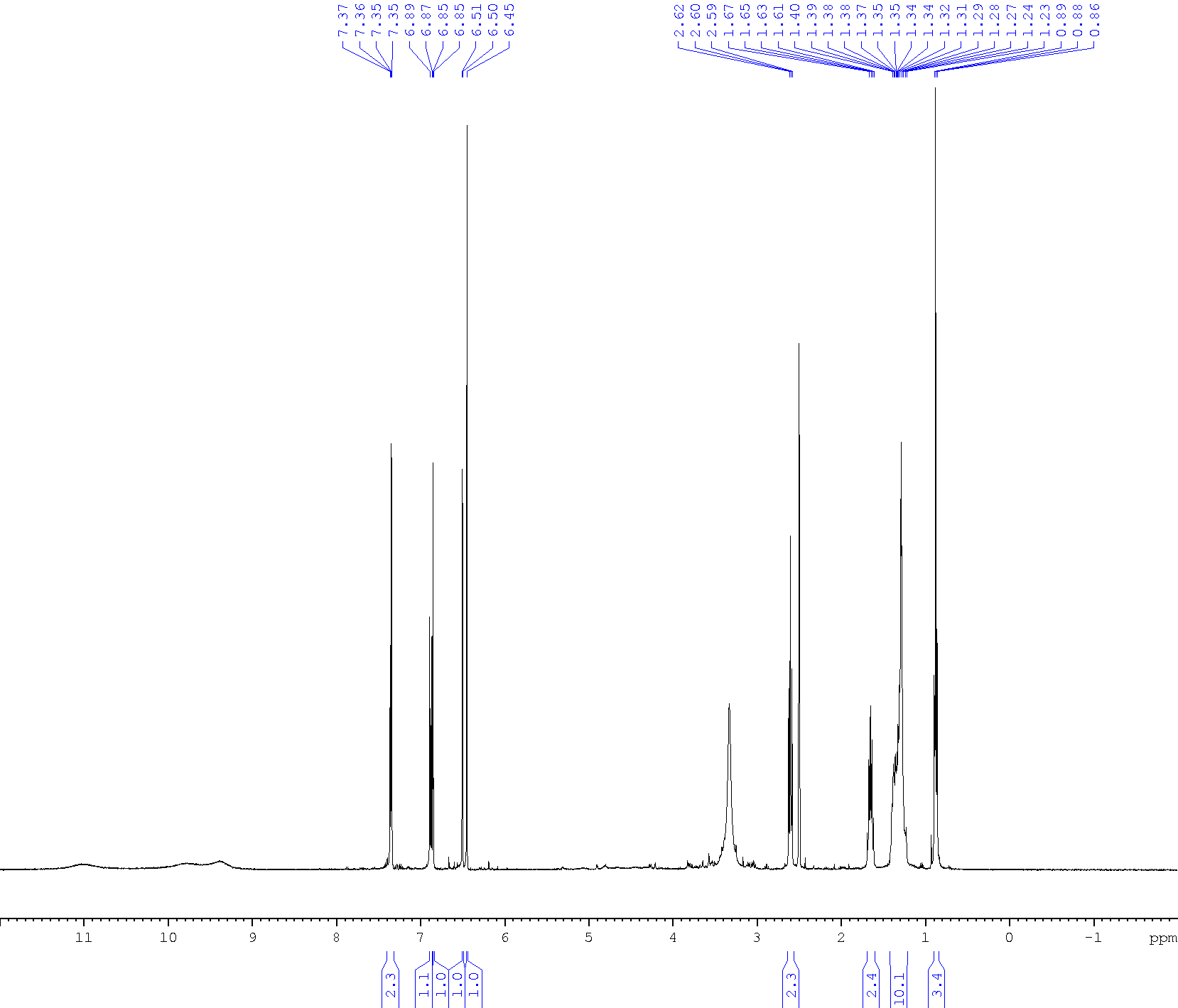


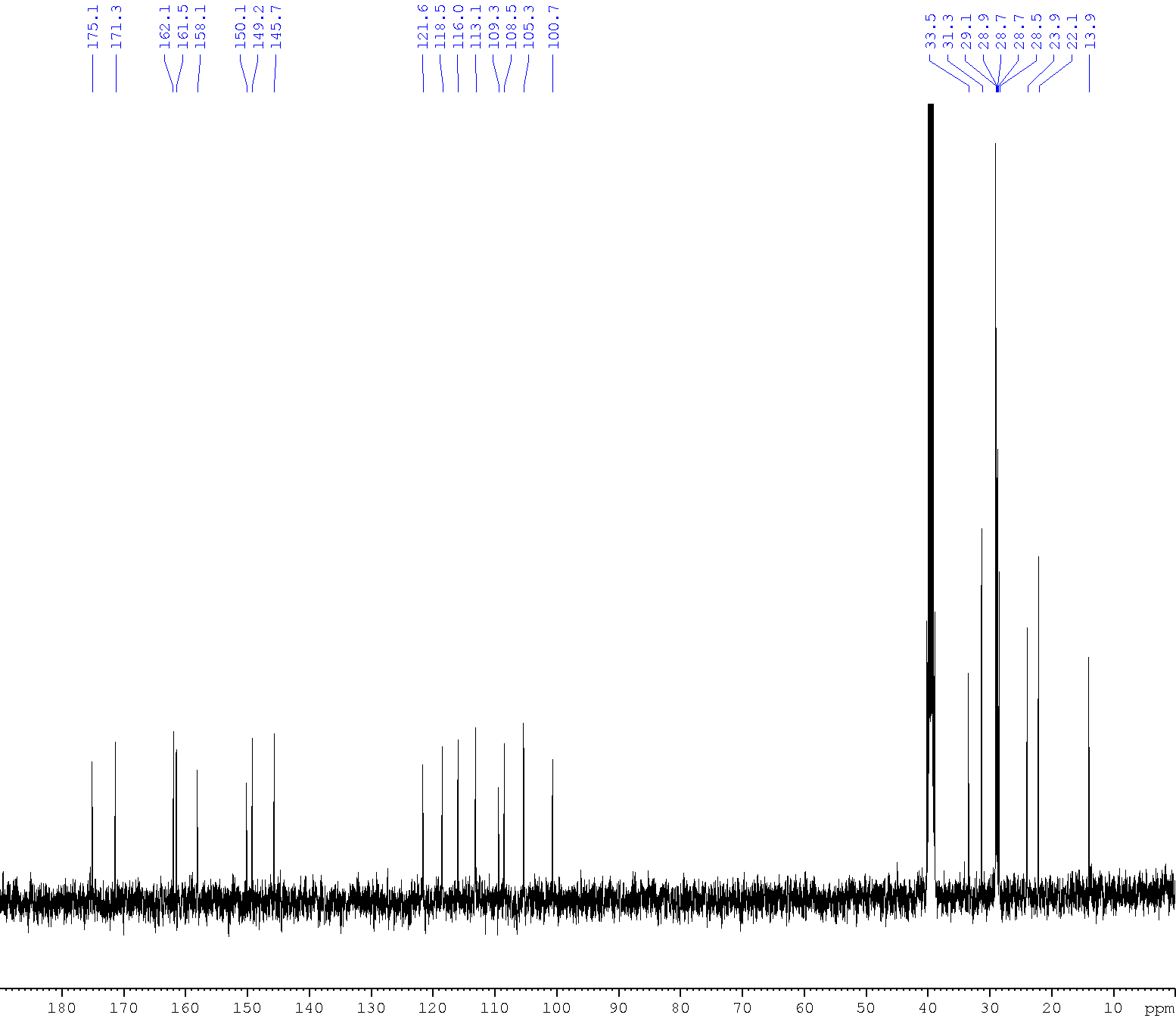


7-(Benzyloxy)-2-(3ʹ,4ʹ-bis(benzyloxy)phenyl)-4-oxo-4*H*-chromen-5-yl dodecanoate (3e)

The reaction was carried out according to general procedure A with **2** (0.1 g, 0.18 mmol), Et_3_N (0.08 mL, 0.54 mmol) and lauroyl chloride (0.09 mL, 0.45 mmol). The crude product was purified by flash chromatography (4:1 Petroleum ether:EtOAC) to give the *title compound* **3e** (90 mg, 68%) as a white solid.

**R_f_:** 0.69 (4:1 Petroleum ether:EtOAC)

**M.P.:** 87 – 90 °C

**δ_H_** (400 MHz; CDCl_3_): 0.88 (3H, t, *J* = 6.8 Hz, 12ʹʹ-H), 1.26 – 1.35 (14H, broad m, 5ʹʹ-H, 6ʹʹ-H, 7ʹʹ-H, 8ʹʹ-H, 9ʹʹ-H, 10ʹʹ-H and 11ʹʹ-H), 1.40 – 1.47 (2H, m, 4ʹʹ-H), 1.81 (2H, p, *J* = 7.6 Hz, 3ʹʹ-H), 2.73 (2H, t, *J* = 6.8 Hz, 2ʹʹ-H), 5.15 (1H, s, 7-*O*CH_2_), 5.23 (2H, s, 3ʹ-*O*CH_2_), 5.24 (2H, s, 4ʹ-*O*CH_2_), 6.44 (1H, s, 3-H), 6.67 (1H, d, *J* = 2.4 Hz, 6-H), 6.89 (1H, d, *J* = 2.4 Hz, 8-H), 7.00 (1H, d, *J* = 8.8 Hz, 5ʹ-H), 7.32-7.48 (17H, m, Ar-H, 2ʹ-H and 6ʹ-H)

**δ_C_** (100 MHz; CDCl_3_): 14.3 (C-12ʹʹ), 22.8 (C-11ʹʹ), 24.6 (C-3ʹʹ), 29.2, 29.4, 29.6, 29.7, 29.7, 29.8 (C-4ʹʹ, C-5ʹʹ, C-6ʹʹ, C-7ʹʹ, C-8ʹʹ and C-9ʹʹ) , 32.0 (C-10ʹʹ), 34.5 (C-2ʹʹ), 70.9 (7-*O*CH_2_), 71.1, 71.8 (3ʹ-*O*CH_2_ and 4ʹ-*O*CH_2_), 100.0 (C-8), 107.5 (C-3), 108.8 (C-6), 111.6 (C-4a), 113.0 (C-2ʹ), 114.3 (C-5ʹ), 120.5 (C-6ʹ), 124.4 (C-1ʹ), 127.3, 127.5, 127.7, 128.2, 128.6, 128.8, 129.0 (Ar-C), 135.6 (7-*O*CH_2_C(Ar)), 136.6, 136.9 (3ʹ-OCH_2_C(Ar) and 4ʹ-*O*CH_2_C(Ar)), 149.0 (C-3ʹ), 150.8 (C-5), 152.0 (C-4ʹ), 158.8 (C-8a), 161.9 (C-2), 162.6 (C-7), 172.5 (C-1ʹʹ), 176.6 (C-4)

**IR:** ν_max_/cm^-1^; 673, 694, 730, 757, 795, 831, 846, 869, 889, 902, 949, 969, 998, 1028, 1156, 1184, 1218, 1276, 1320, 1337, 1353, 1374, 1432, 1454, 1517, 1608, 1632, 1651, 1683, 1715, 1734, 1755, 1980, 2166, 2852, 2922

**HRMS (ESI^+^):** Found (MNa^+^) 761.3471, C_48_H_50_NaO­_7_ requires 761.3449

**Supplementary Figure 9:** ^1^H and ^13^C NMR of **3e**


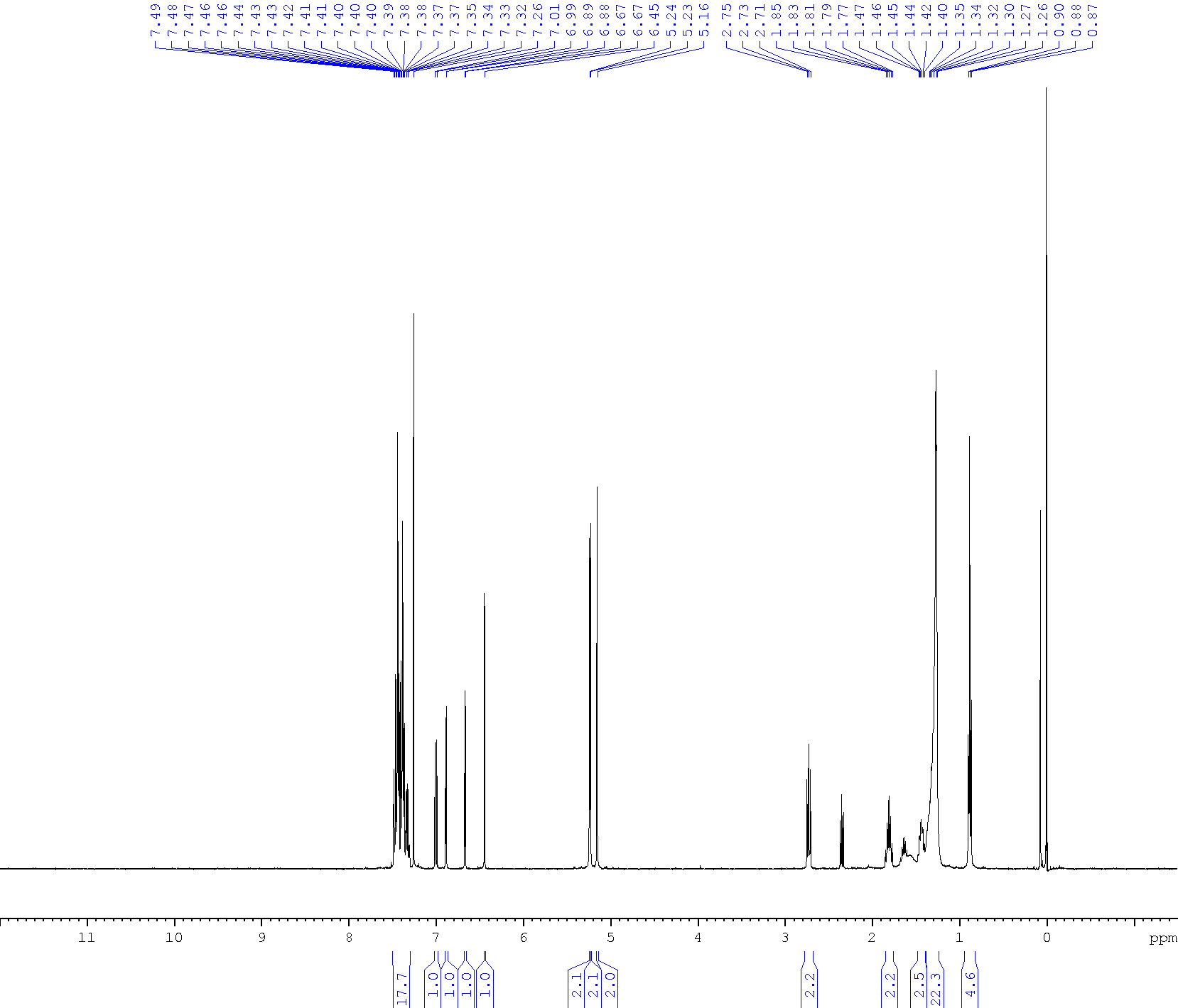


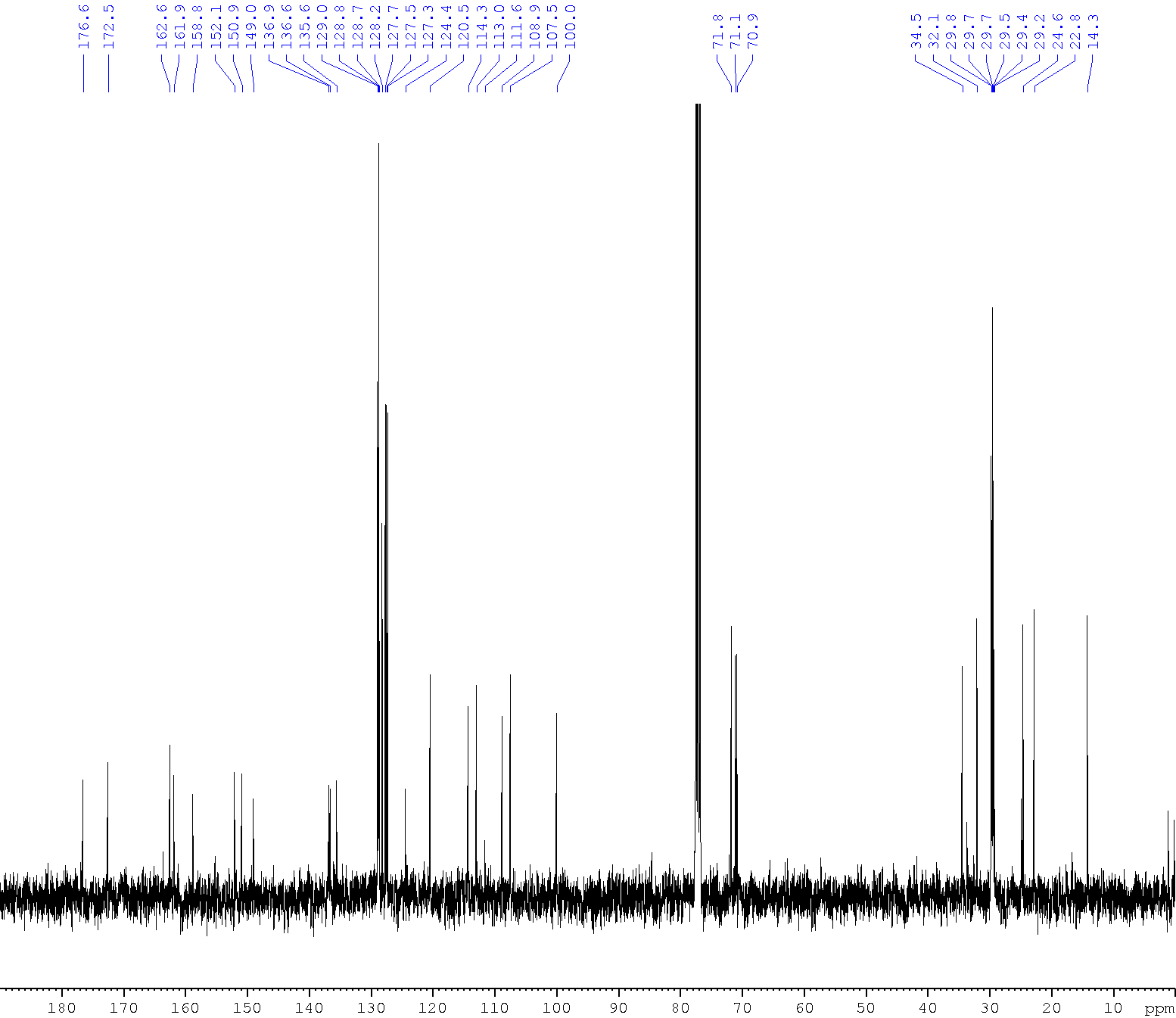


2-(3ʹ,4ʹ-Dihydroxyphenyl)-7-hydroxy-4-oxo-4*H*-chromen-5-yl dodecanoate (4e)

The reaction was carried out according to general procedure B with **3e** (0.1 g, 0.14 mmol) and 20% Pd(OH)_2_/C (19 mg, 0.02 mmol). The reaction was stirred for 24 h. The crude product was purified by flash chromatography (1:1 Petroleum ether:EtOAC) to give the *title compound* **4e** (63 mg, quant.) as a white solid.

**R_f_:** 0.45 (1:2 Petroleum ether:EtOAC)

**M.P.:** 168 – 171 °C

**δ_H_** (400 MHz; d_6_-DMSO): 0.85 (3H, t, *J* = 6.8 Hz, 12ʹʹ -H), 1.25 – 1.31 (14H, broad m, 5ʹʹ-H, 6ʹʹ-H, 7ʹʹ-H, 8ʹʹ-H, 9ʹʹ-H, 10ʹʹ-H and 11ʹʹ-H), 1.33 – 1.39 (2H, m, 4ʹʹ-H), 1.64 (2H, t, *J* = 7.5 Hz, 3ʹʹ-H), 2.60 (2H, t, *J* = 7.5, 2ʺ-H), 6.45 (1H, s, 3-H), 6.50 (1H, d, *J* = 2.5 Hz, 6-H), 6.85 (1H, d, *J* = 2.5 Hz, 8-H), 6.88 (1H, d, *J* = 8.8 Hz, 5ʹ-H), 7.34-7.37 (2, m 2ʹ-H and 6ʹ-H), 9.37 (1H, s, 4ʹ-OH), 9.82 (1H, s, 3ʹ-OH), 11.03 (1H, s, 7-OH)

**δ_C_** (100 MHz; d_6_-DMSO): 13.9 (C-12ʹʹ), 22.1 (C-11ʹʹ) 23.9 (C-3ʹʹ), 28.4, 28.7, 28.7, 28.9, 29.0 (C-4ʹʹ, C-5ʹʹ, C-6ʹʹ, C-7ʹʹ, C-8ʹʹ and C-9ʹʹ), 31.3 (C-10ʹʹ), 33.4 (C-2ʺ), 100.6 (C-8), 105.3 (C-3), 108.5 (C-6), 109.3 (C-4a), 113.1 (C-2ʹ), 115.9 (C-5ʹ), 118.4 (C-6ʹ), 121.6 (C-1ʹ), 145.7 (C-3ʹ), 149.2 (C-4ʹ), 150.1 (C-5), 158.1 (C-8a), 161.4 (C-2), 162.1 (C-7), 171.3 (C-1ʹʹ), 175.0 (C-4)

**IR:** ν_max_/cm^-1^; 682, 730, 789, 815, 845, 947, 997, 1027, 1112, 1157, 1180, 1207, 1263, 1296, 1314, 1388, 1441, 1504, 1524, 1591, 1606, 1634, 1722, 2854, 2923, 3249, 3533

**HRMS (ESI^+^):** Found (MNa^+^) 491.2034, C_27_H_32_NaO­_7_ requires 491.2040

**Supplementary Figure 10:** ^1^H and ^13^C NMR of **4e**


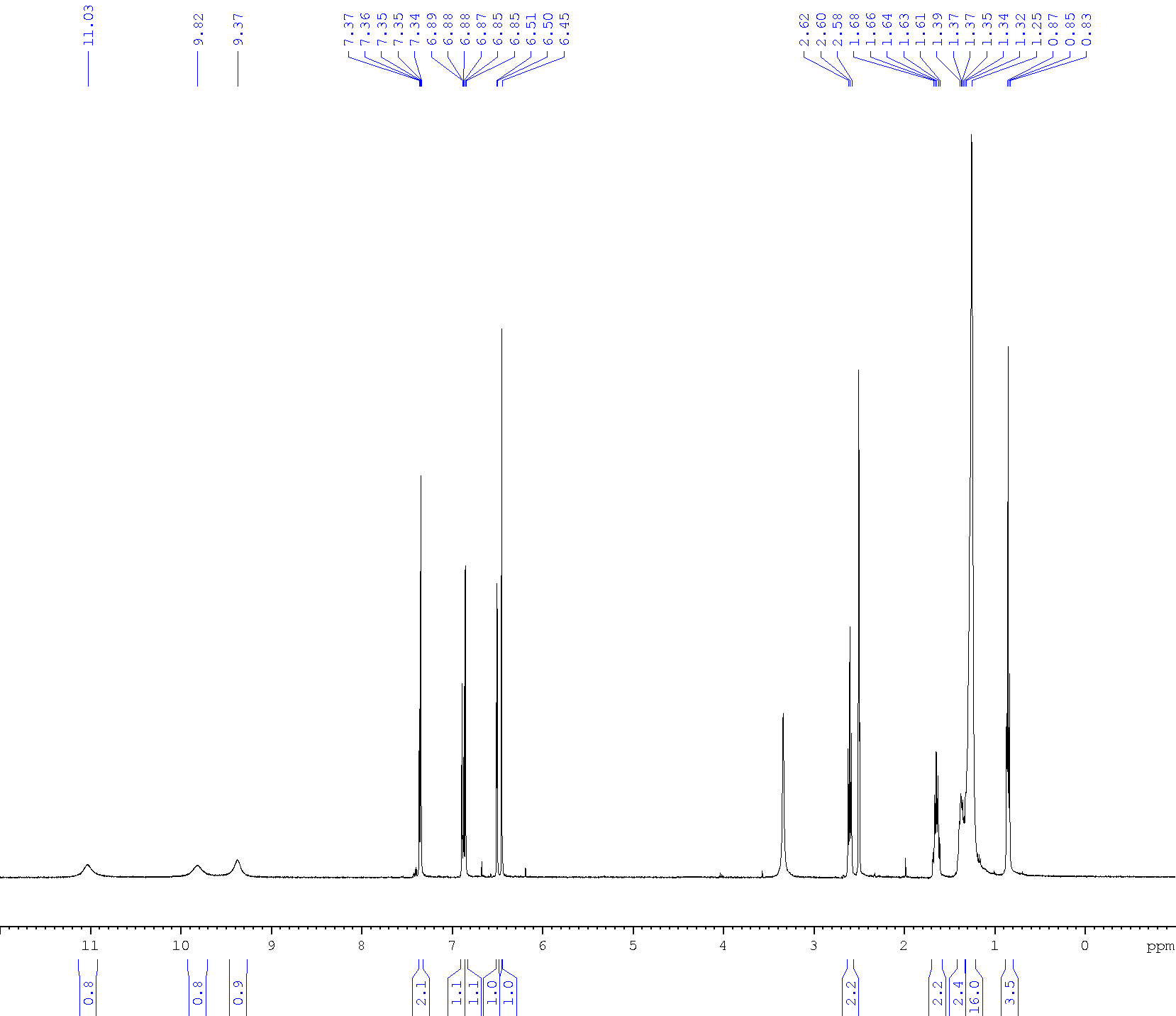


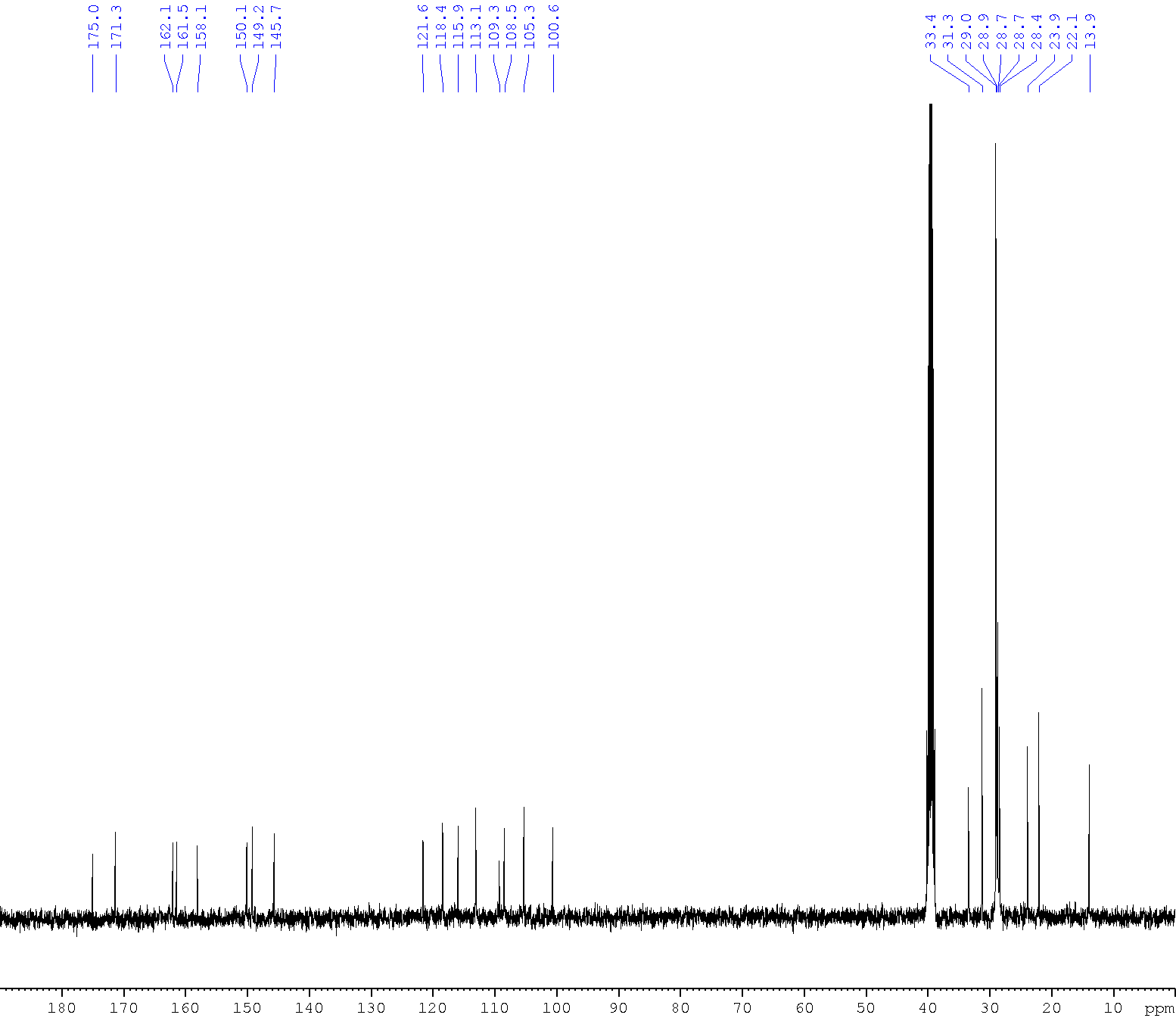


7-(Benzyloxy)-2-(3ʹ,4ʹ-bis(benzyloxy)phenyl)-4-oxo-4*H*-chromen-5-yl palmitate (3f)

The reaction was carried out according to general procedure A with **2** (0.2 g, 0.36 mmol), Et_3_N (0.15 mL, 1.08 mmol) and palmitoyl chloride (0.22 mL, 0.72 mmol). The crude product was purified by flash chromatography (4:1 Petroleum ether:EtOAC) to give the *title compound* **3f** (0.23 g, 82%) as a white solid.

**R_f_:** 0.77 (4:1 Petroleum ether:EtOAC)

**M.P.:** 75 – 78 °C

**δ_H_** (400 MHz; CDCl_3_): 0.89 (3H, t, *J* = 7.0 Hz, 16ʹʹ-H), 1.24 – 1.39 (22H, broad m, 5ʹʹ-H, 6ʹʹ-H, 7ʹʹ-H, 8ʹʹ-H, 9ʹʹ-H, 10ʹʹ-H, 11ʹʹ-H, 12ʹʹ-H, 13ʹʹ-H, 14ʹʹ-H and 15ʹʹ-H), 1.41 – 1.48 (2H, m, 4ʹʹ-H), 1.82 (2H, p, *J* = 7.7 Hz, 3ʹʹ-H), 2.74 (2H, t, *J* = 7.7 Hz, 2ʹʹ-H), 5.14 (2H, s, 7-*O*CH_2_), 5.23 (2H, s, 3ʹ-*O*CH_2_), 5.24 (2H, s, 4ʹ-*O*CH_2_), 6.45 (1H, s, 3-H), 6.67 (1H, d, *J* = 2.4 Hz, 6-H), 6.88 (1H, d, *J* = 2.4 Hz, 8-H), 7.00 (1H, d, *J* = 8.4 Hz, 5ʹ-H), 7.31 – 7.49 (17H, m, Ar-H, 2ʹ-H and 6ʹ-H)

**δ_C_** (100 MHz; CDCl_3_): 14.2 (C-16ʹʹ), 22.8 (C-15ʹʹ), 24.6 (C-3ʹʹ), 29.2, 29.3, 29.4, 29.5, 29.6, 29.7, 29.8 (C-4ʹʹ, C-5ʹʹ, C-6ʹʹ, C-7ʹʹ, C-8ʹʹ, C-9ʹʹ, C-10ʹʹ, C-11ʹʹ, C-12ʹʹ and C-13ʹʹ), 32.0 (C-14ʹʹ), 34.4 (C-2ʹʹ), 70.7 (7-*O*CH_2_), 71.0, 71.6 (3ʹ-*O*CH_2_ and 4ʹ-*O*CH_2_), 99.9 (C-8), 107.3 (C-3), 108.8 (C-6), 111.5 (C-4a), 112.7 (C-2ʹ), 114.2 (C-5ʹ), 120.3 (C-6ʹ), 124.2 (C-1ʹ), 127.2, 127.5, 127.6, 128.5, 128.7, 128.8 (Ar-C), 135.5 (7-*O*CH_2_C(Ar)), 136.6, 136.8 (3ʹ-*O*CH_2_C(Ar) and 4ʹ-*O*CH_2_C(Ar)), 148.9 (C-3ʹ), 150.7 (C-5), 152.0 (C-4ʹ), 158.7 (C-8a), 161.8 (C-2), 162.5 (C-7), 172.5 (C-1ʹʹ), 176.5 (C-4)

**IR:** ν_max_/cm^-1^; 695, 731, 795, 832, 857, 909, 946, 1010, 1104, 1190, 1207, 1264, 1315, 1345, 1373, 1431, 1455, 1498, 1518, 1607, 1631, 1650, 1716, 1755, 2852, 2921, 3035

**HRMS (ESI^+^):** Found (MNa^+^) 817.4075, C_52_H_58_NaO_7_ requires 817.4075

**Supplementary Figure 11:** ^1^H and ^13^C NMR of **3f**


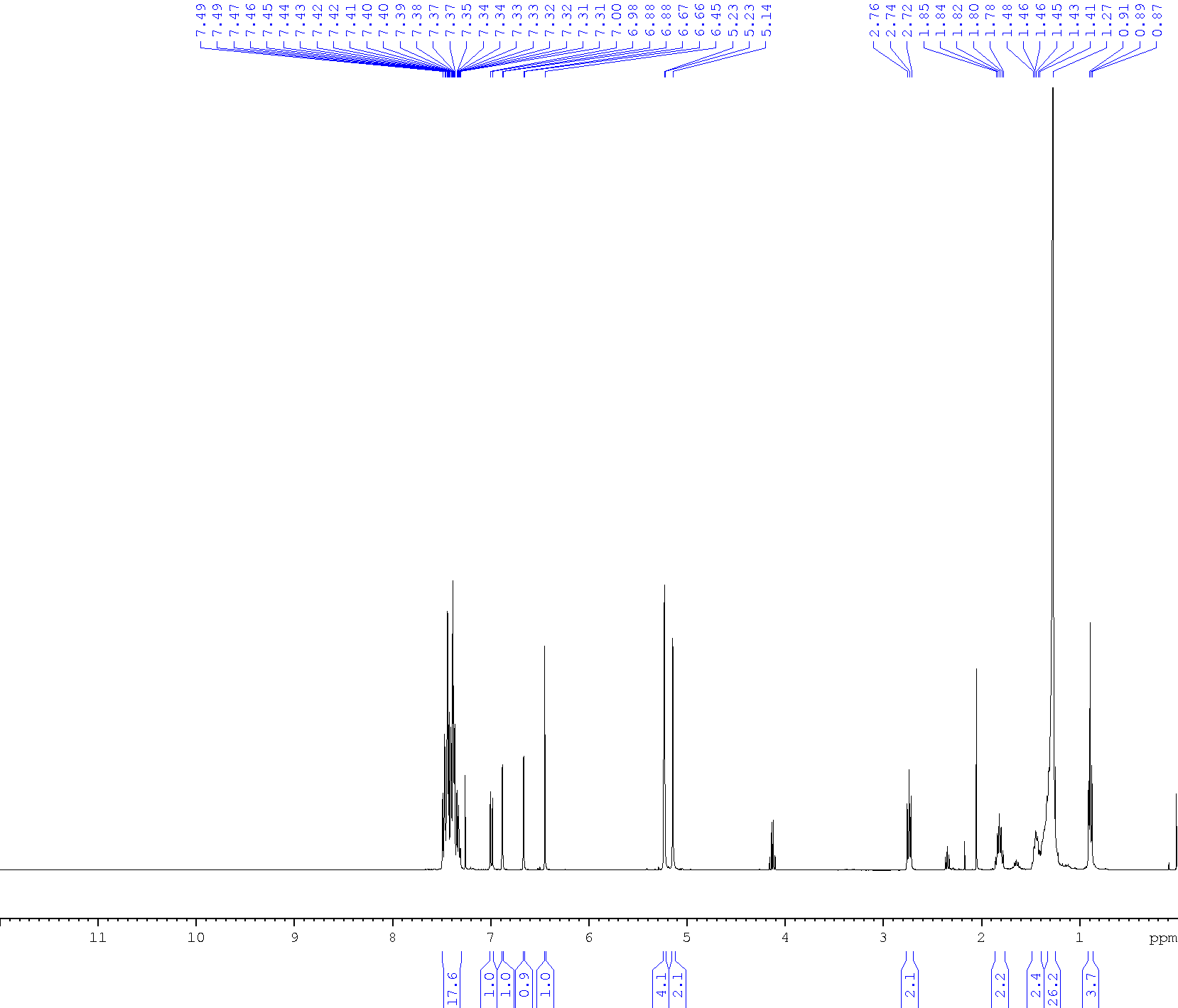


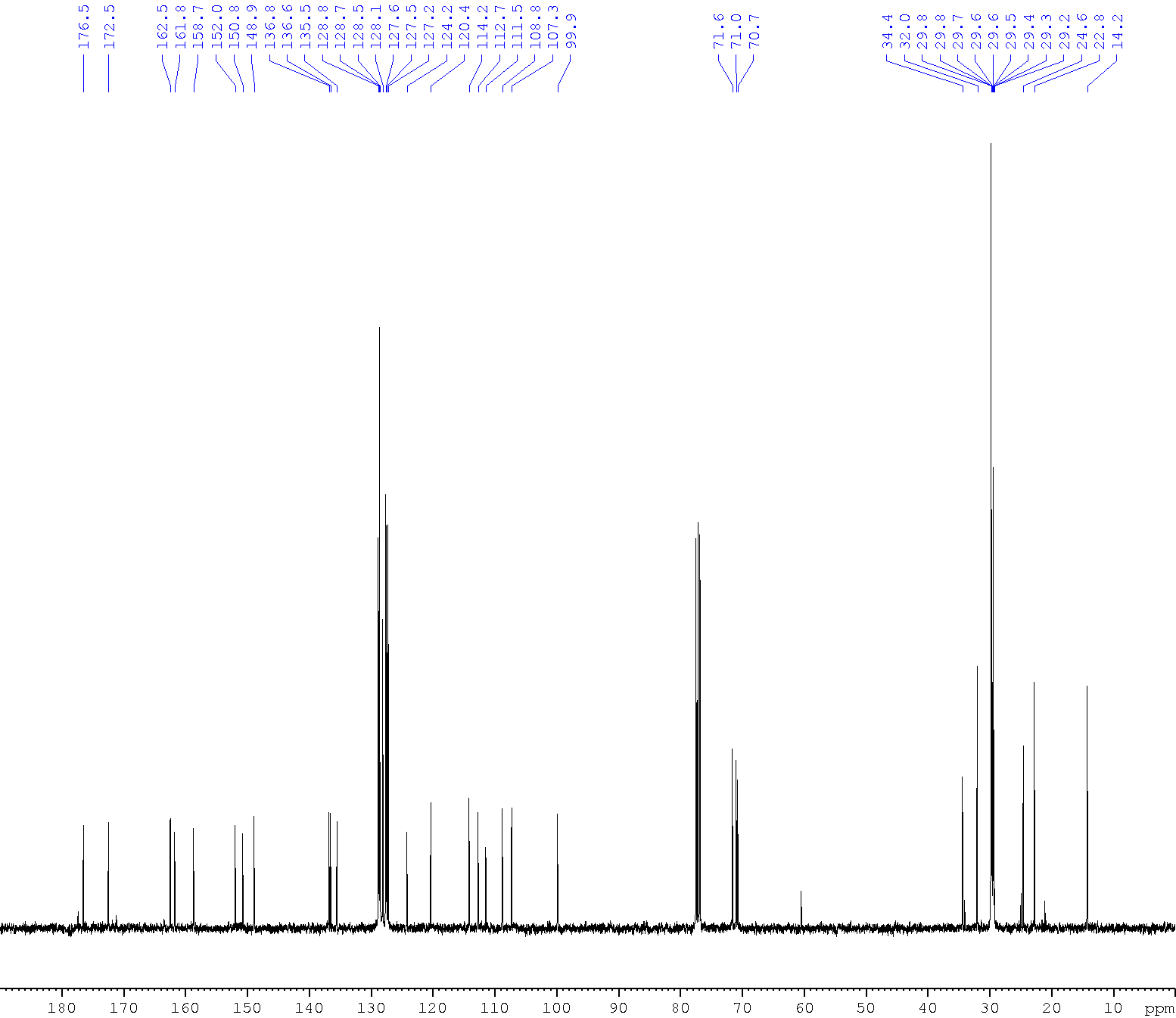


2-(3ʹ,4ʹ-Dihydroxyphenyl)-7-hydroxy-4-oxo-4*H*-chromen-5-yl palmitate (4f)

The reaction was carried out according to general procedure B with **3f** (0.14 g, 0.18 mmol) and 20% Pd(OH)_2_/C (25 mg, 0.04 mmol). The reaction was stirred for 24 h. The crude product was purified by flash chromatography (1:1 Petroleum ether:EtOAC) to give the *title compound* **4f** (72 mg, 76%) as a white solid.

**R_f_:** 0.53 (1:2 Petroleum ether:EtOAC)

**M.P.:** 164 – 167 °C

**δ_H_** (400 MHz; d_6_-DMSO): 0.85 (3H, t, *J* = 6.8 Hz, 16ʹʹ-H), 1.17 – 1.30 (22H, broad m, 5ʹʹ-H, 6ʹʹ-H, 7ʹʹ-H, 8ʹʹ-H, 9ʹʹ-H, 10ʹʹ-H, 11ʹʹ-H, 12ʹʹ-H, 13ʹʹ-H, 14ʹʹ-H and 15ʹʹ-H), 1.32 – 1.39 (2H, m, 4ʹʹ-H), 1.65 (2H, t, *J* = 7.4 Hz, 3ʹʹ-H), 2.60 (2H, t, *J* = 7.4, 2ʹʹ-H), 6.44 (1H, s, 3-H), 6.50 (1H, d, *J* = 2.3 Hz, 6-H), 6.85 (1H, d, *J* = 2.3 Hz, 8-H), 6.88 (1H, d, *J* = 9.0 Hz, 5ʹ-H), 7.33-7.36 (2, m 2ʹ-H and 6ʹ-H)

**δ_C_** (100 MHz; d_6_-DMSO): 13.9 (C-16ʹʹ), 22.1 (C-15ʹʹ), 23.9 (C-3ʹʹ), 28.4, 28.7, 28.9, 29.0 (C-4ʹʹ, C-5ʹʹ, C-6ʹʹ, C-7ʹʹ, C-8ʹʹ, C-9ʹʹ, C-10ʹʹ, C-11ʹʹ, C-12ʹʹ and C-13ʹʹ), 31.3 (C-14ʹʹ), 33.4 (C-2ʹʹ), 100.6 (C-8), 105.3 (C-3), 108.5 (C-6), 109.3 (C-4a), 113.1 (C-2ʹ), 115.9 (C-5ʹ), 118.4 (C-6ʹ), 121.6 (C-1ʹ), 145.7 (C-3ʹ), 149.2 (C-4ʹ), 150.1 (C-5), 158.1 (C-8a), 161.5 (C-2), 162.1 (C-7), 171.3 (C-1ʹʹ), 175.0 (C-4)

**IR:** ν_max_/cm^-1^; 684, 696, 717, 786, 814, 853, 920, 947, 1001, 1031, 1111, 1147, 1163, 1193, 1220, 1241, 1271, 1284, 1321, 1384, 1412, 1441, 1471, 1502, 1527, 1576, 1616, 1628, 1741, 2035, 2162, 2851, 2918, 3339, 3491

**HRMS (ESI^+^):** Found (MNa^+^) 547.2655, C_31_H_40_NaO­_7_ requires 547.2666

**Supplementary Figure 12:** ^1^H and ^13^C NMR of **4f**


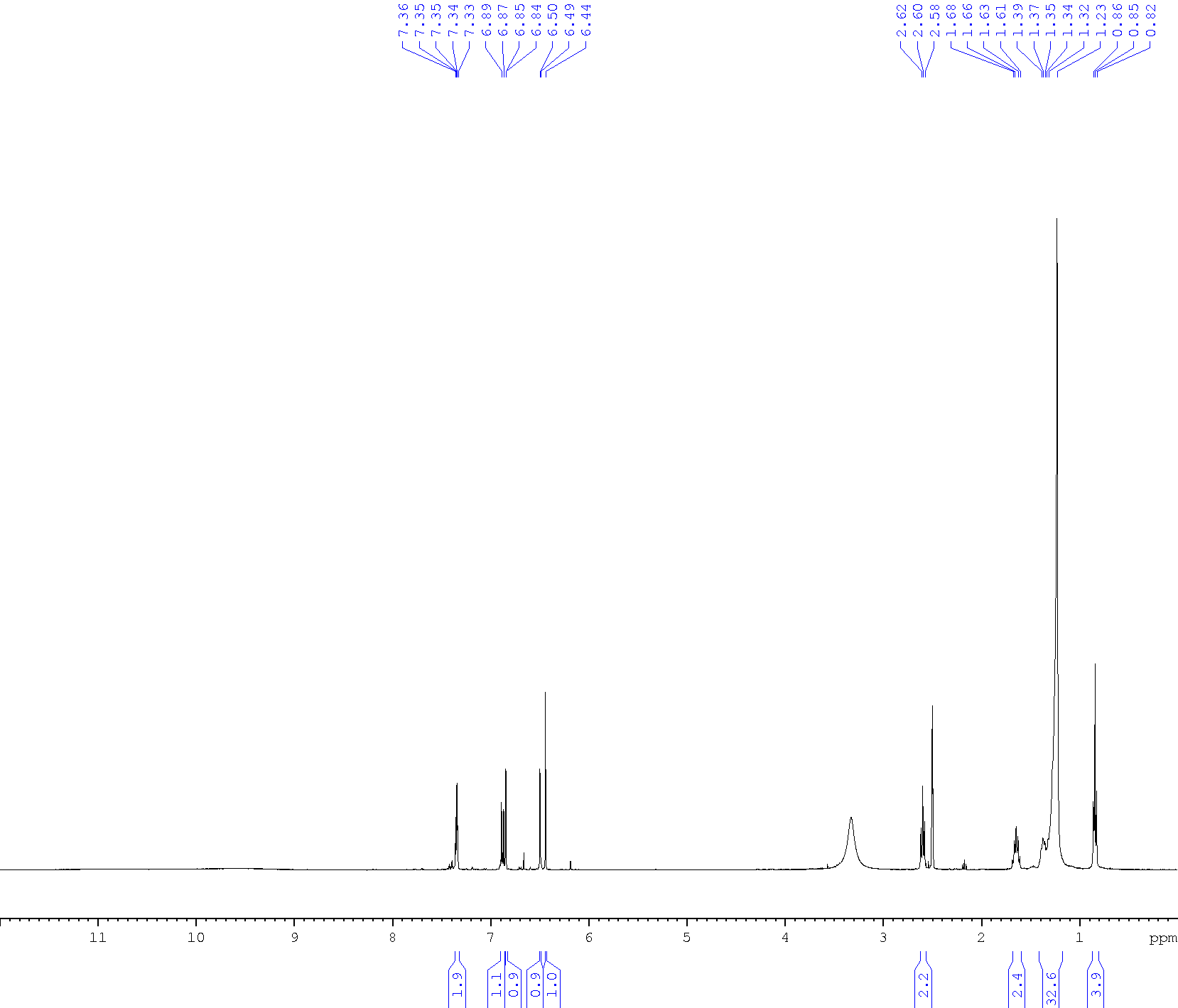


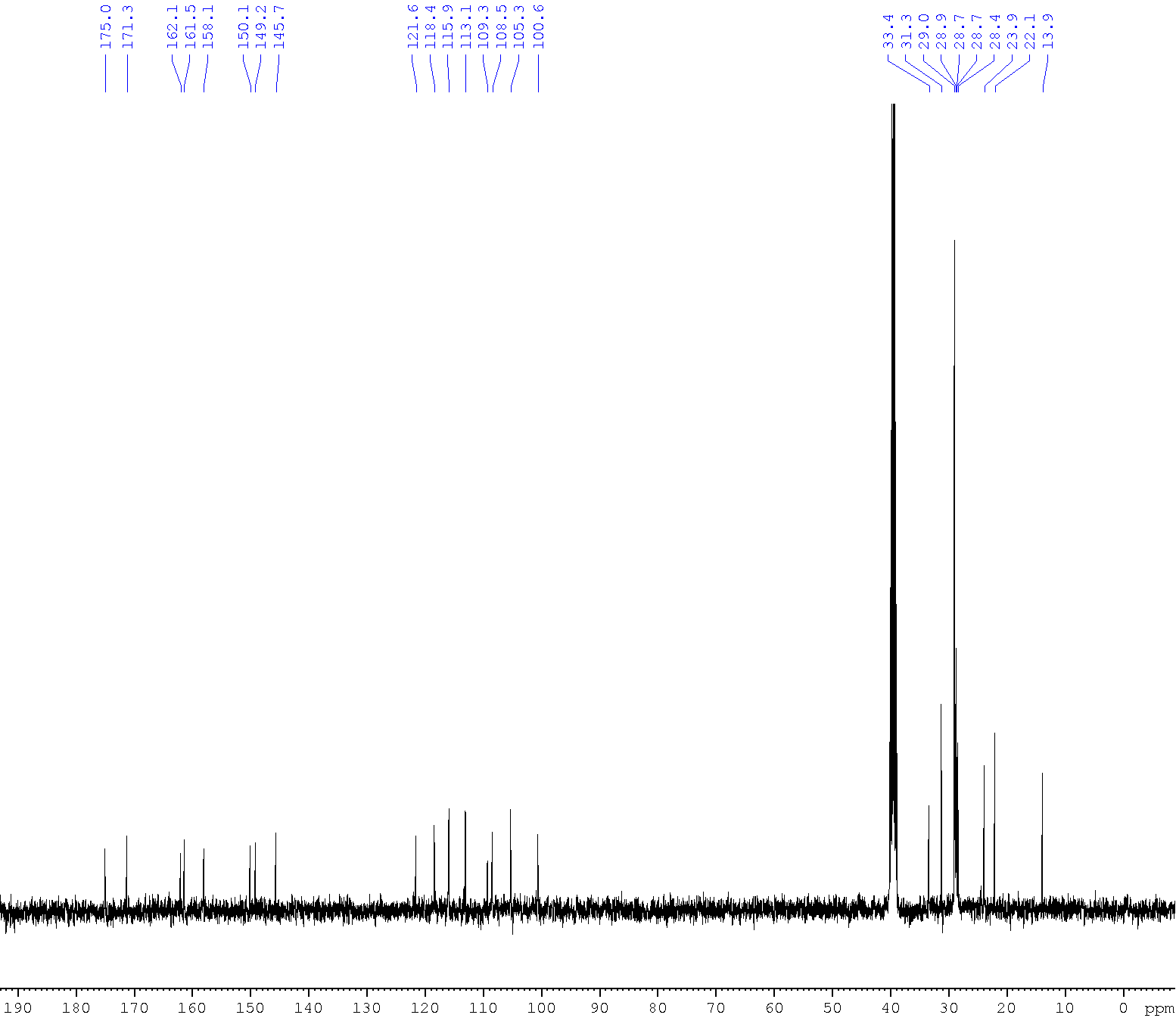


2-(3ʹ,4ʹ-Dihydroxyphenyl)-7-hydroxy-4-oxo-4*H*-chromen-5-yl methyl succinate (4g)

The reaction was carried out firstly according to general procedure A with **2** (0.27 g, 0.49 mmol), Et_3_N (0.21 mL, 1.47 mmol) and methyl succinyl chloride (0.15 mL, 1.23 mmol) to give a crude ester which was taken to the next step without further purification. Then according to general procedure B using the above produced ester (0.36 g, 0.54 mmol) and 20% Pd(OH)_2_/C (76 mg, 0.11 mmol). The reaction was stirred for 24 h. The crude product was purified by flash chromatography (1:3 Petroleum ether:EtOAC) to give the *title compound* **4g** (70 mg, 32% over two steps) as a yellow solid.

**R_f_:** 0.26 (1:3 Petroleum ether:EtOAC)

**M.P.:** 180 – 183 °C

**δ_H_** (400 MHz; d_6_-DMSO): 2.70 (2H, t, *J* = 7.0 Hz, 2ʹʹ-H or 3ʹʹ-H), 2.90 (2H, t, *J* = 7.0 Hz, 2ʹʹ-H or 3ʹʹ-H), 3.63 (3H, s, 6ʹʹ-H), 6.51 (1H, d, *J* = 2.0 Hz, 6-H), 6.85 (1H, d, *J* = 2.0 Hz, 8-H), 6.89 (1H, d, *J* = 8.8 Hz, 5ʹ-H), 7.35 – 7.37 (2H, m, 2ʹ-H and 6ʹ-H)

**δ_C_** (100 MHz; d_6_-DMSO): 28.4, 29.0 (C-2ʹʹ and C-3ʹʹ), 51.5 (C-6ʹʹ), 100.8 (C-8), 105.3 (C-3), 108.4 (C-6′), 109.1 (C-4a), 113.1 (C-2ʹ), 116.0 (C-5ʹ), 118.5 (C-6ʹ), 121.6 (C-1ʹ), 145.7 (C-3ʹ), 149.3 (C-4ʹ), 149.9 (C-5), 158.1 (C-8a), 161.6 (C-2), 162.1 (C-7), 170.4 (C-1ʹʹ), 172.3 (C-4ʹʹ), 175.1 (C-4)

**IR:** ν_max_/cm^-1^; 667, 684, 695, 738, 770, 786, 808, 850, 865, 879, 894, 931, 952, 969, 997, 1020, 1115, 1140, 1164, 1186, 1220, 1238, 1271, 1305, 1320, 1368, 1395, 1414, 1440, 1505, 1519, 1555, 1579, 1599, 1634, 1722, 1746, 1759, 2548, 2854, 2923, 2956, 3413, 3503

**HRMS (ESI^+^):** Found (MNa^+^) 423.0685, C20H16NaO9 requires 423.0687

**Supplementary Figure 13:** ^1^H and ^13^C NMR of **4g**


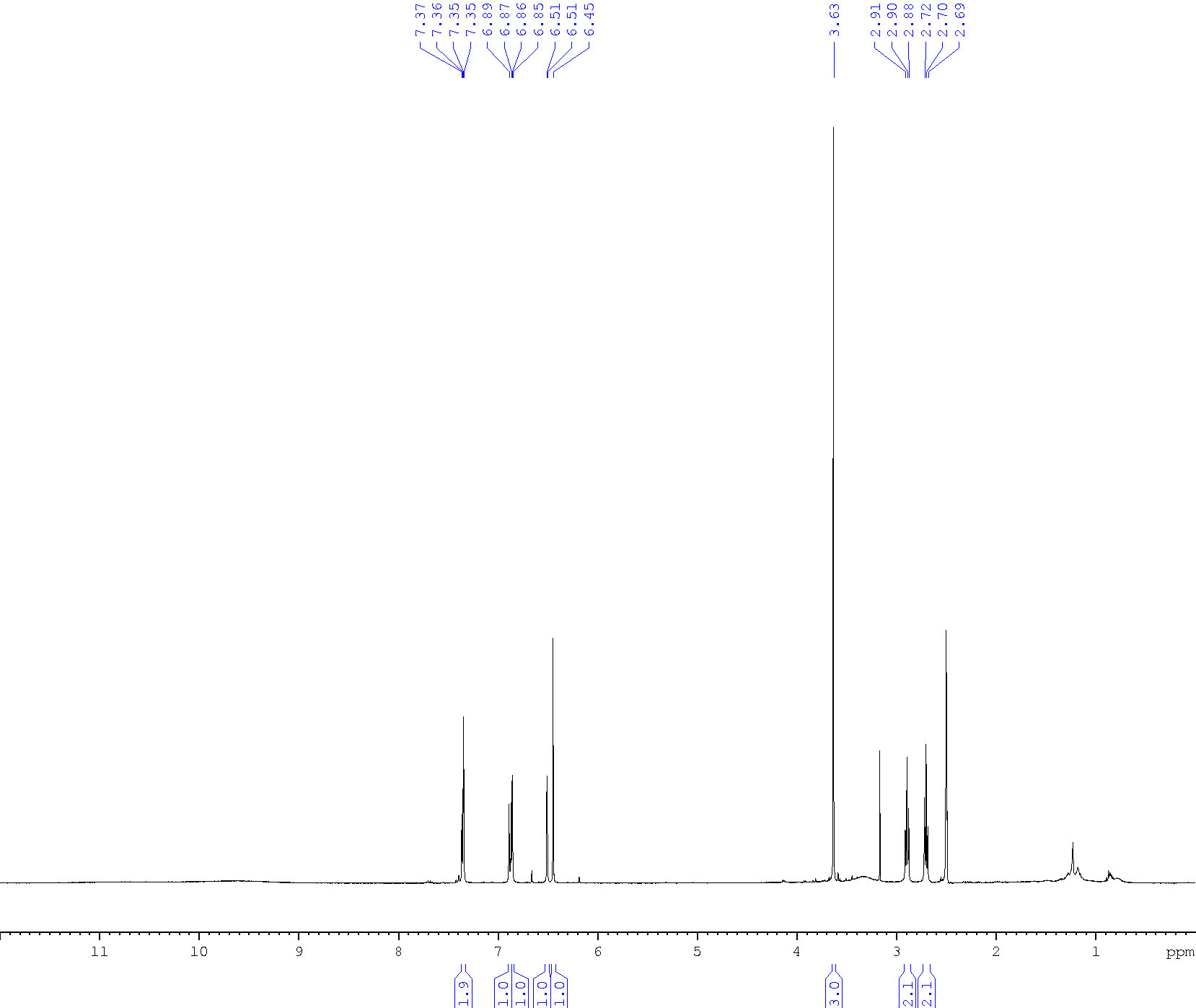


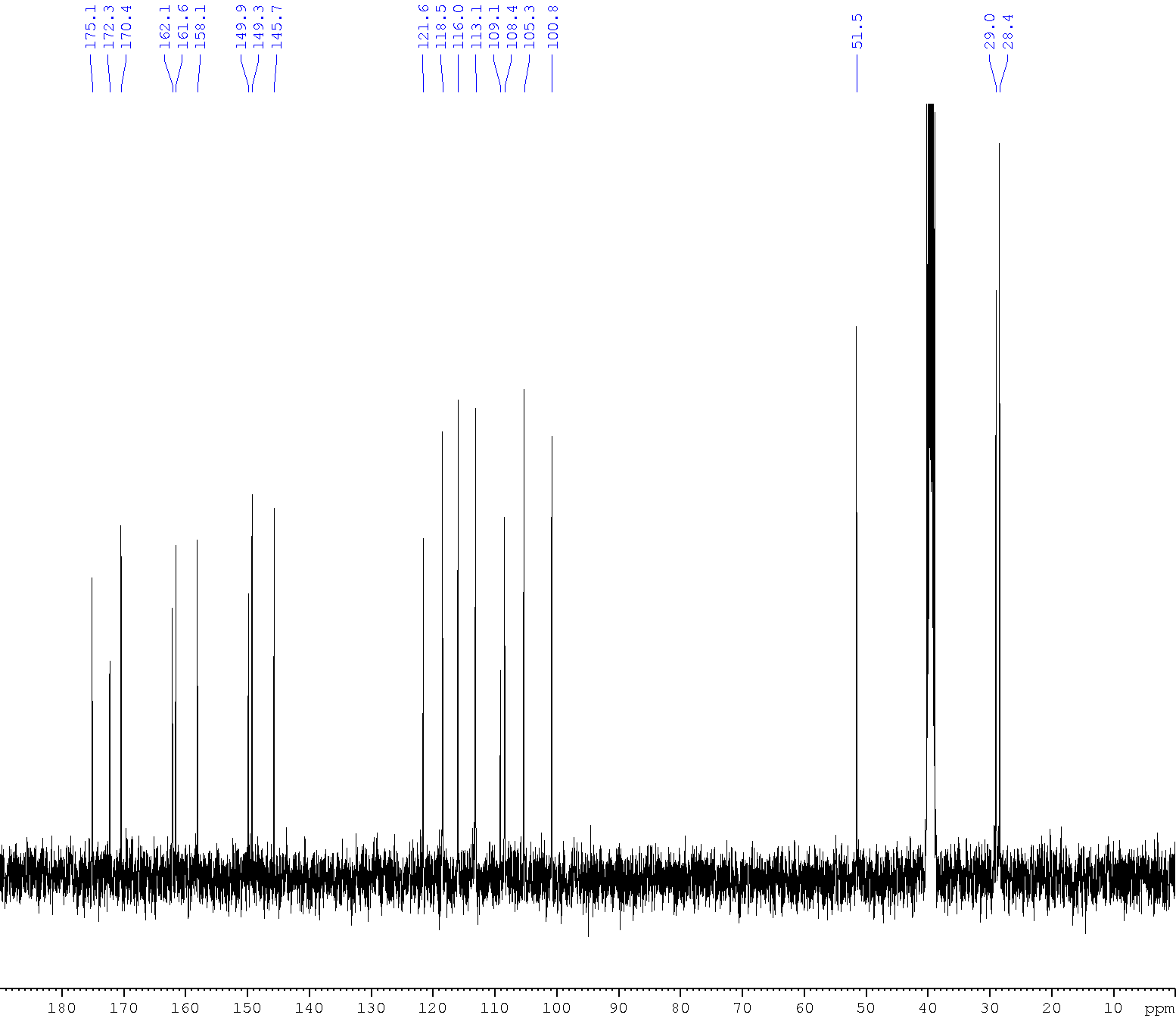


7-(Benzyloxy)-2-(3ʹ,4ʹ-bis(benzyloxy)phenyl)-4-oxo-4*H*-chromen-5-yl benzoate (3h)

The reaction was carried out according to general procedure A with **2** (0.2 g, 0.36 mmol), Et_3_N (0.15 mL, 1.08 mmol) and benzoyl chloride (0.11 mL, 0.72 mmol). The crude product was purified by flash chromatography (3:1 Petroleum ether:EtOAC) to give the *title compound* **3h** (0.19 g, 80%) as a white solid.

**R_f_:** 0.38 (3:1 Petroleum ether:EtOAC)

**M.P.:** 156 – 158 °C

**δ_H_** (400 MHz; CDCl_3_): 5.18 (2H, s, 7-*O*CH_2_), 5.23 (2H, s, 3ʹ-*O*CH_2_), 5.24 (2H, s, 4ʹ-*O*CH_2_), 6.39 (1H, s, 3-H), 6.82 (1H, d, *J* = 2.5 Hz, 6-H), 6.94 (1H¸ d, *J* = 2.5 Hz, 8-H), 7.00 (1H, d, 5ʹ-H), 7.29 – 7.53 (17H, m, Ar-H, 2ʹ-H and 6ʹ-H), 7.51 (2H, t, *J* = 7.5 Hz, 3ʹʹʹ-H), 7.63 (1H, tt, *J* = 1.6, 7.5 Hz, 4ʹʹʹ-H), 8.26 (2H, dd, *J* = 1.6, 7.0 Hz, 2ʹʹʹ-H)

**δ_C_** (100 MHz; CDCl_3_): 70.9 (7-*O*CH_2_), 71.1, 71.8 (3ʹ-*O*CH_2_ and 4ʹ-*O*CH_2_), 100.2 (C-8), 107.5 (C-3), 109.0 (C-6), 111.6 (C-8a), 113.0 (C-2ʹ), 114.3 (C-5ʹ), 120.5 (C-6ʹ), 124.4 (C-1ʹ), 127.3, 127.5, 127.7, 128.6, 128.7, 128.8, 129.0 (Ar-C), 128.2 (C-3ʹʹʹ), 130.0 (C-1ʹʹʹ), 130.6 (C-2ʹʹʹ), 133.5 (C-4ʹʹʹ), 135.6 (7-*O*CH_2_C(Ar)), 136.6, 136.9 (3ʹ-*O*CH_2_C(Ar) and 4ʹ-*O*CH_2_C(Ar)), 149.0 (C-3ʹ), 151.0 (C-5), 152.1 (C-4ʹ), 158.8 (C-4a), 161.9 (C-2), 162.6 (C-7), 165.5 (C-1ʹʹ), 176.4 (C-4)

**IR:** ν_max_/cm^-1^; 672, 695, 732, 765, 797, 827, 845, 858, 885, 909, 936, 1021, 1062, 1101, 1152, 1190, 1205, 1250, 1262, 1287, 1323, 1351, 1374, 1431, 1453, 1497, 1515, 1609, 1630, 1647, 1732, 2922, 3033

**HRMS (ESI^+^):** Found (MNa^+^) 683.2013, C_43_H_32_NaO­_7_ requires 683.2040

**Supplementary Figure 14:** ^1^H and ^13^C NMR of **3h**


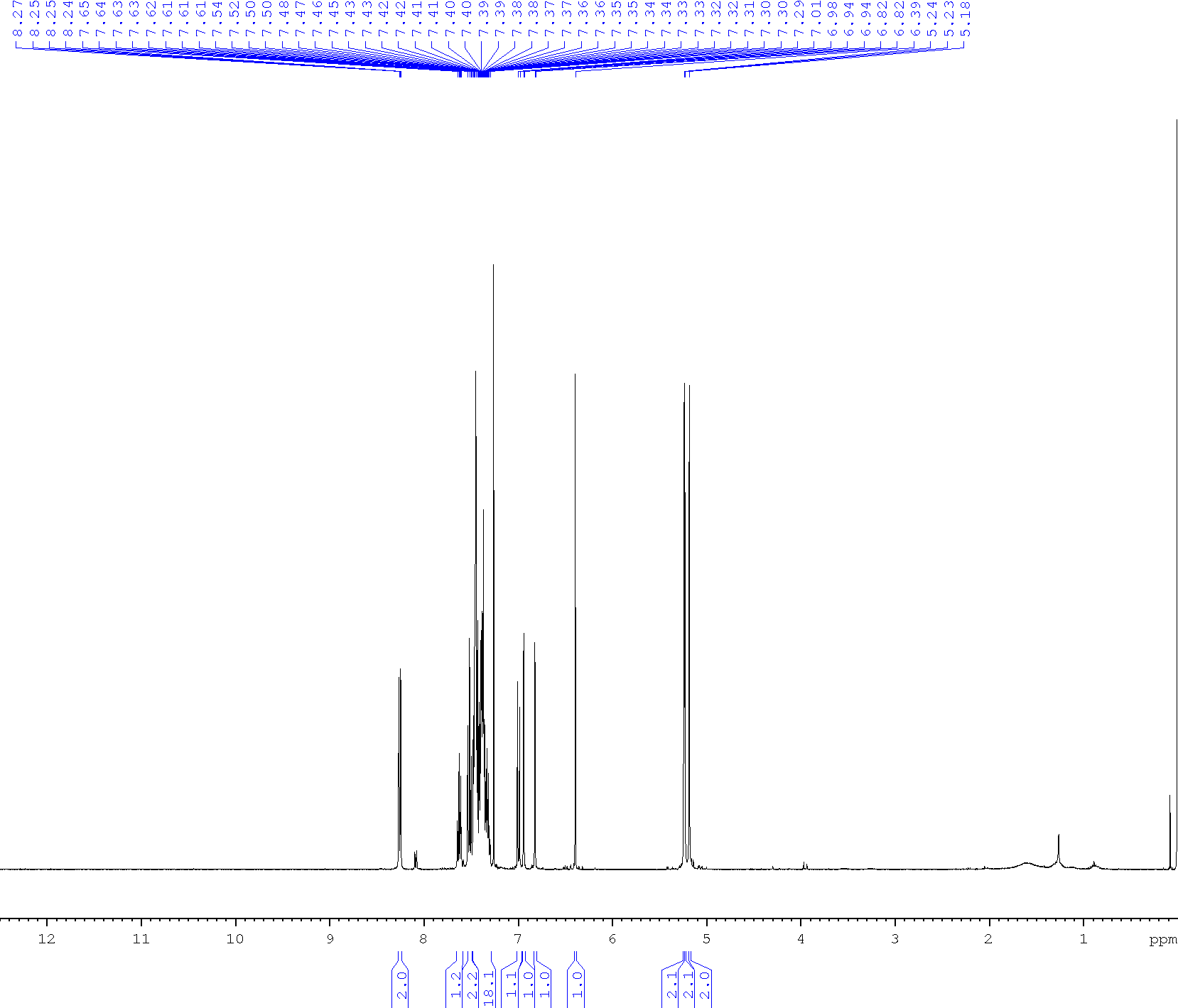


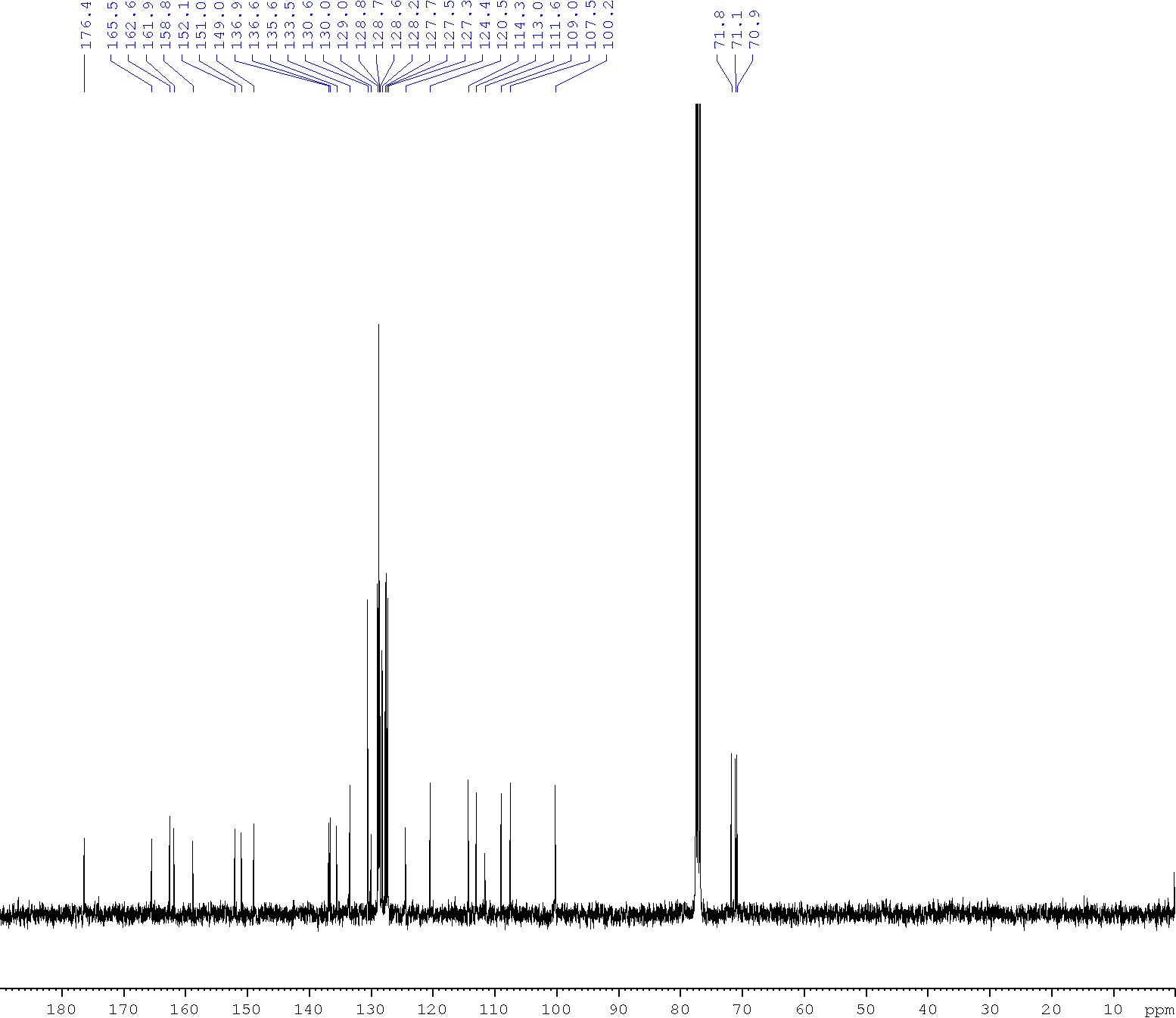


2-(3′,4′-Dihydroxyphenyl)-7-hydroxy-4-oxo-4*H*-chromen-5-yl benzoate (4h)

The reaction was carried out according to general procedure B with **3h** (0.18 g, 0.27 mmol) and 10% Pd/C (57 mg, 0.05 mmol). The reaction was stirred for 24 h. The product was purified by flash chromatography (1:3 Petroleum ether:EtOAC) to give the *title compound* **4h** (81 mg, 78%) as a yellow solid.

**R_f_:** 0.39 (2:1 Petroleum ether:EtOAC)

**M.P.:** 180 – 183 °C

**δ_H_** (400 MHz; d_6_-DMSO): 6.40 (1H, s, 3-H), 6.70 (1H, d, *J* = 2.3 Hz, 6-H), 6.89 (1H, d, *J* = 9.0 Hz, 5ʹ-H), 6.92 (1H, d, *J* = 2.3 Hz, 8-H), 7.35 – 7.38 (2H, m, 2ʹ-H and 6ʹ-H), 7.60 (2H, t, *J* = 7.5 Hz, 3ʹʹʹ-H), 7.76 (1H, tt, *J* = 1.5, 7.5 Hz, 4ʹʹʹ-H), 8.10 (2H, dd, *J* = 1.5, 7.5 Hz, 2ʹʹʹ-H)

**δ_C_** (100 MHz; d_6_-DMSO): 100.9 (C-8), 105.3 (C-3), 108.8 (C-6), 109.4 (C-4a), 113.1 (C-2ʹ), 116.0 (C-5ʹ), 118.5 (C-6ʹ), 121.6 (C-1ʹ), 128.7 (C-3ʹʹʹ), 129.6 (C-1ʹʹʹ), 129.9 (C-2ʹʹʹ), 133.6 (C-4ʹʹʹ), 145.7 (C-3ʹ), 149.2 (C-4ʹ), 150.1 (C-5), 158.1 (C-8a), 161.6 (C-2), 162.0 (C-7), 164.4 (C-1ʹʹ), 175.0 (C-4)

**IR:** ν_max_/cm^-1^; 685, 702, 768, 788, 815, 838, 861, 948, 998, 1027, 1060, 1091, 1159, 1189, 1253, 1361, 1389, 1443, 1500, 1557, 1600, 1709, 2162, 2580, 2928, 3081

**HRMS (ESI^+^):** Found (MNa^+^) 413.0639, C_22_H_14_NaO_7_ requires 413.0632

The ^1^H NMR values are in agreement with literature.^1^

**Supplementary Figure 15:** ^1^H and ^13^C NMR of **4h**


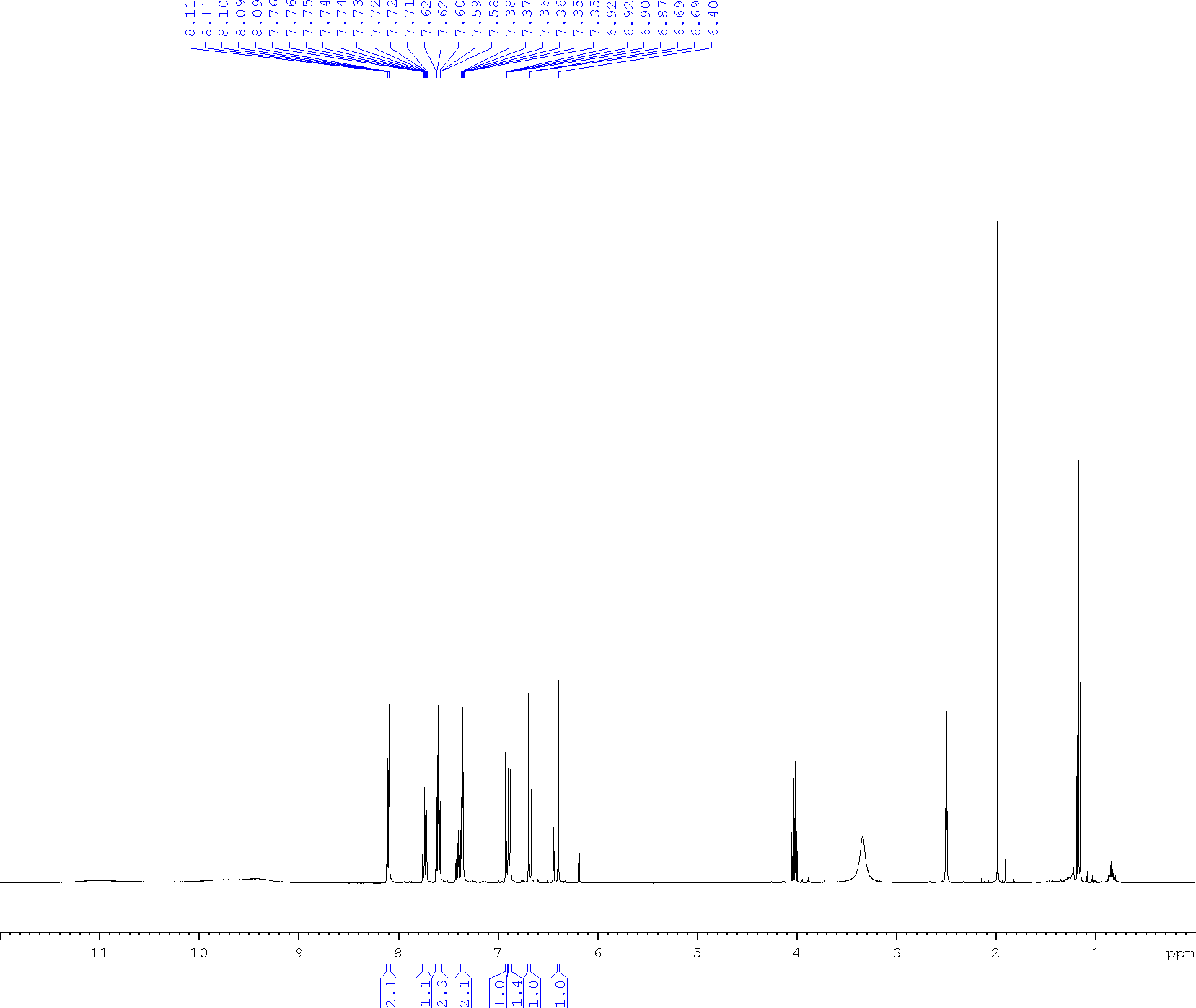


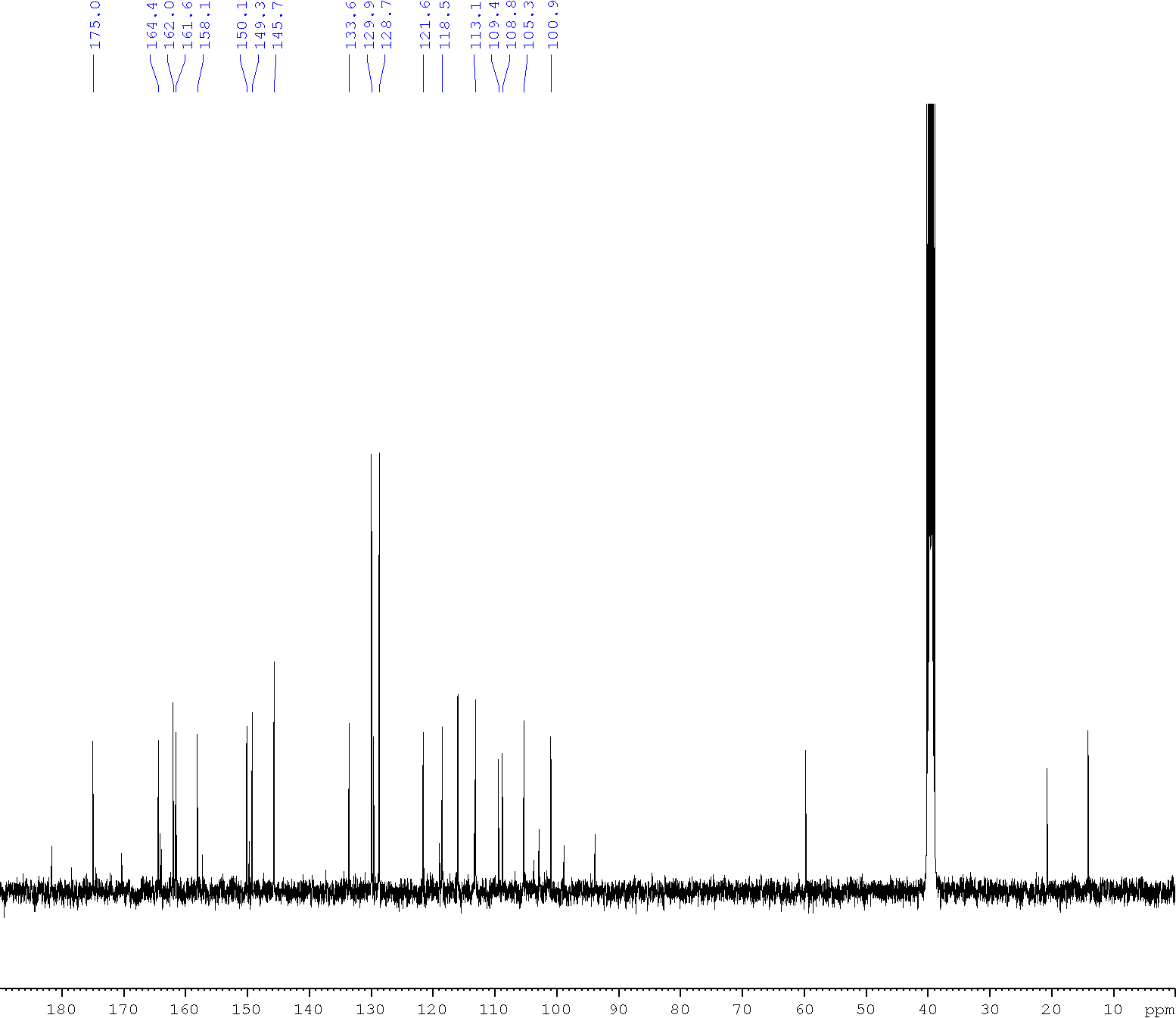


7-(Benzyloxy)-2-(3ʹ,4ʹ-bis(benzyloxy)phenyl)-4-oxo-4*H*-chromen-5-yl 2ʺ-phenylacetate (3i)

The reaction was carried out according to general procedure A with **2** (0.1 g, 0.18 mmol), Et_3_N (0.08 mL, 0.57 mmol) and phenyl acetyl chloride (0.05 mL, 0.36 mmol). The product was purified by flash chromatography (3:1 Petroleum ether:EtOAC) to give the *title compound* **3i** (79 mg, 65%) as a yellow solid.

**R_f_:** 0.61 (3:1 Petroleum ether:EtOAC)

**M.P.:** 126 – 129 °C

**δ_H_** (400 MHz; CDCl_3_): 4.08 (2H, s, 2ʹʹ-H), 5.14 (2H, s, 7-*O*CH_2_), 5.23 (2H, s, 3ʹ-*O*CH_2_), 5.24 (2H, s, 4ʹ-*O*CH_2_), 6.47 (1H, s, 3-H), 6.64 (1H, d, *J* = 2.4 Hz, 6-H), 6.89 (1H, d, *J* = 2.4 Hz, 8-H), 7.01 (1H, d, *J* = 8.5 Hz, 5ʹ-H), 7.29 – 7.49 (22H, m, Ar-H, 2ʹ-H, 6ʹ-H, 2ʹʹʹ-H, 3ʹʹʹ-H and 4ʹʹʹ-H)

**δ_C_** (100 MHz; CDCl_3_): 41.2 (C-2ʹʹ), 70.9 (7-*O*CH_2_), 71.1, 71.2 (3ʹ-*O*CH_2_ and 4ʹ-*O*CH_2_), 100.2 (C-8), 107.5 (C-3), 108.8 (C-6), 111.4 (C-4a), 113.0 (C-2ʹ), 114.3 (C-5ʹ), 120.5 (C-6ʹ), 124.4 (C-1ʹ), 127.2, 127.3, 127.5, 127.7, 128.2, 128.7, 128.8, 128.9, 129.4 130.0 (C-1ʹʹʹ, C-2ʹʹʹ, C-3ʹʹʹ, C-4ʹʹʹ and Ar-C), 133.8 (C-5ʹʹ), 135.5 (7-*O*CH_2_C(Ar)), 136.6, 136.9 (3ʹ-*O*CH_2_C(Ar) and 4ʹ-*O*CH_2_C(Ar)), 149.0 (C-3ʹ), 150.7 (C-5), 152.1 (C-4ʹ), 158.8 (C-8a), 162.0 (C-2), 162.6 (C-7), 170.4 (C-1ʹʹ), 176.5 (C-4)

**IR:** ν_max_/cm^-1^; 621, 645, 694, 727, 788, 841, 908, 942, 1026, 1103, 1156, 1212, 1259, 1323, 1352, 1378, 1431, 1454, 1497, 1510, 1604, 1632, 1763, 1921, 2929, 3031, 3064

**HRMS (ESI^+^):** Found (MNa^+^) 697.2177, C_44_H_34_NaO_7_ requires 697.2197

**Supplementary Figure 16:** ^1^H and ^13^C NMR of **3i**


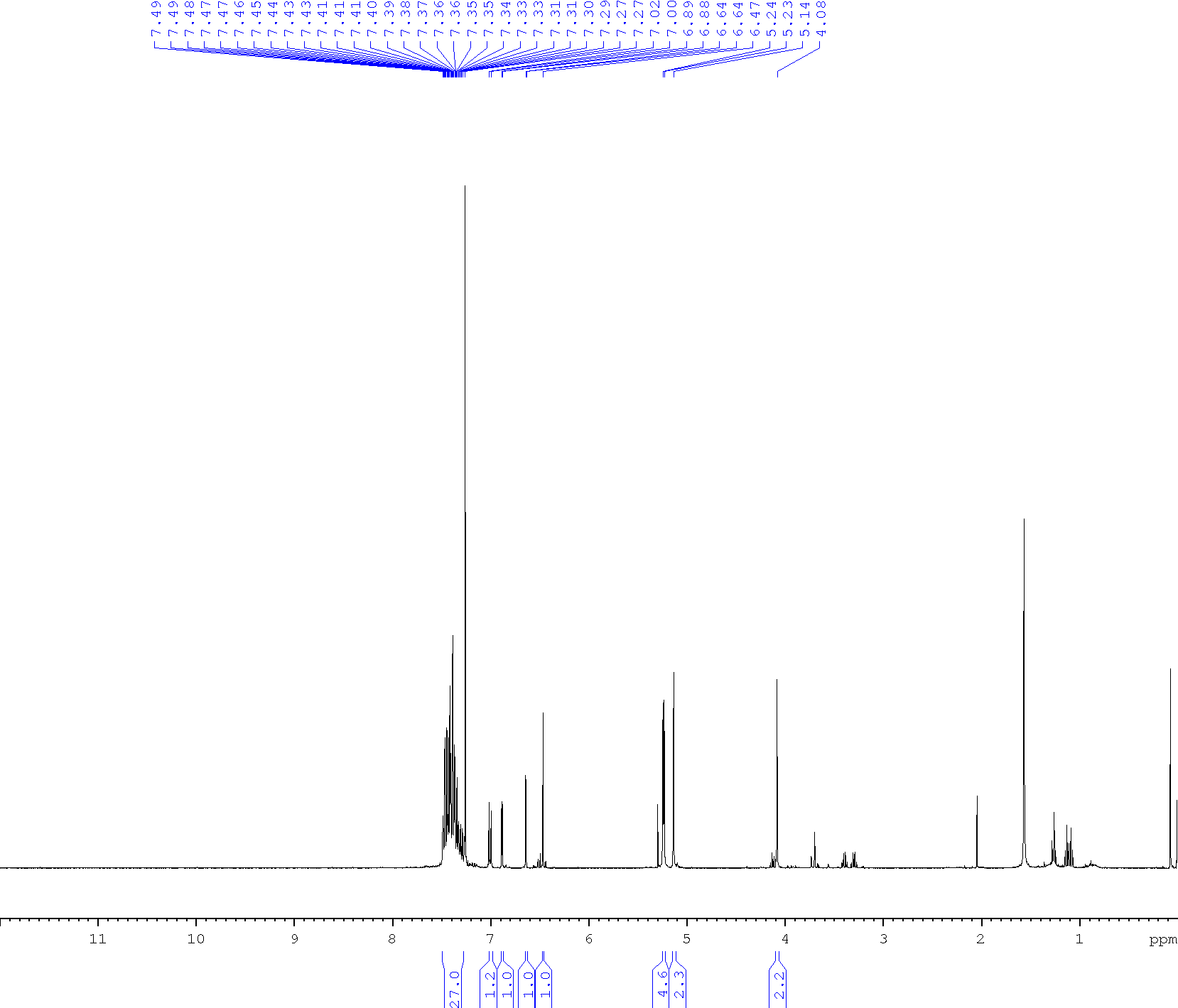


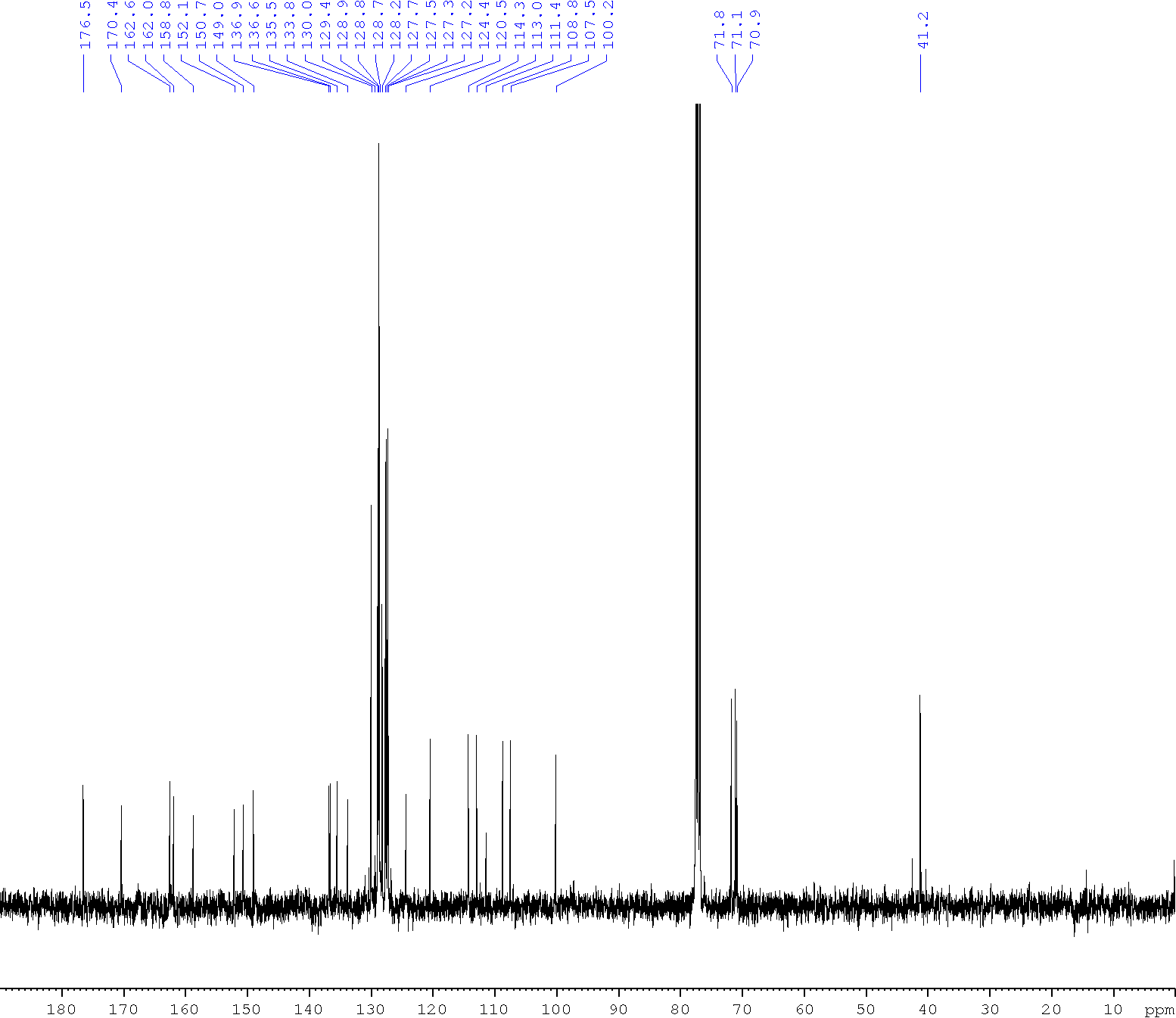


2-(3ʹ,4ʹ-Dihydroxyphenyl)-7-hydroxy-4-oxo-4H-chromen-5-yl 2ʺ-phenylacetate (4i)

The reaction was carried out according to general procedure B with **3i** (96 mg, 0.14 mmol) and 10% Pd/C (30 mg, 0.03 mmol). The reaction was stirred for 24 h. The crude product was purified by flash chromatography (1:3 Petroleum ether:EtOAC) to give the *title compound* **4i** (31 mg, 54%) as a yellow solid.

**R_f_:** 0.55 (2:1 Petroleum ether:EtOAC)

**M.P.:** 195 – 198 °C

**δ_H_** (400 MHz; d_6_-DMSO): 4.02 (2H, s, 2ʺ-H), 6.51 (1H, s, 3-H), 6.53 (1H, d, *J* = 2.1 Hz, 6-H), 6.87 (1H, d, *J* = 2.1 Hz, 8-H), 6.89 (1H, d, *J* = 9.0 Hz, 5ʹ-H), 7.28 (1H, tt, *J* = 1.5, 7.0 Hz, 4ʹʹʹ-H), 7.34-7.42 (6H, m, 2ʹ-H, 6ʹ-H, 2ʹʹʹ-H and 3ʹʹʹ-H), 9.37 (1H, s, 4ʹ-OH), 9.81 (1H, s, 3ʹ-OH), 11.06 (1H, s, 7-OH)

**δ_C_** (100 MHz; d_6_-DMSO): 40.0 (C-2ʹʹ), 100.8 (C-8), 105.3 (C-3), 108.3 (C-6), 109.2 (C-4a), 113.2 (C-2ʹ), 116.0 (C-5ʹ), 118.5 (C-6ʹ), 121.6 (C-1ʹ), 126.9 (C-4ʹʹʹ), 128.3 (C-3ʹʹʹ), 129.9 (C-2ʹʹʹ), 134.0 (C-1ʹʹʹ), 145.7 (C-3ʹ), 149.3 (C-4ʹ), 150.0 (C-5), 158.1 (C-8a), 161.6 (C-2), 162.0 (C-7), 169.8 (C-1ʹʹ), 175.1 (C-4)

**IR:** ν_max_/cm^-1^; 683, 698, 719, 761, 788, 814, 848, 949, 1000, 1023, 1077, 1112, 1153, 1220, 1263, 1304, 1351, 1386, 1446, 1499, 1515, 1564, 1604, 1624, 1718, 2036, 2163, 3298

**HRMS (ESI^+^):** Found (MNa^+^) 427.0795, C_23_H_16_NaO_7_ requires 427.0788

**Supplementary Figure 17:** ^1^H and ^13^C NMR of **4i**


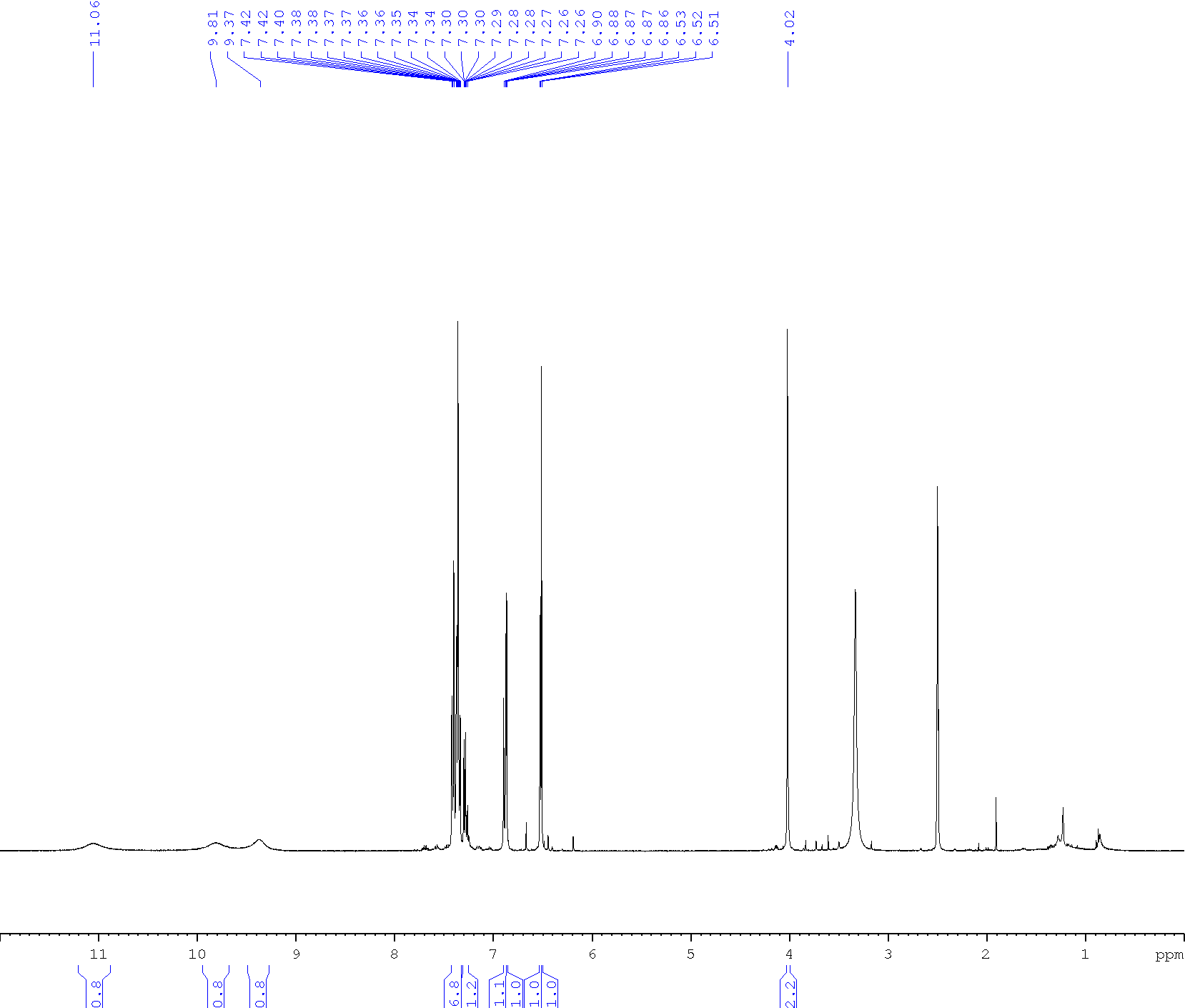


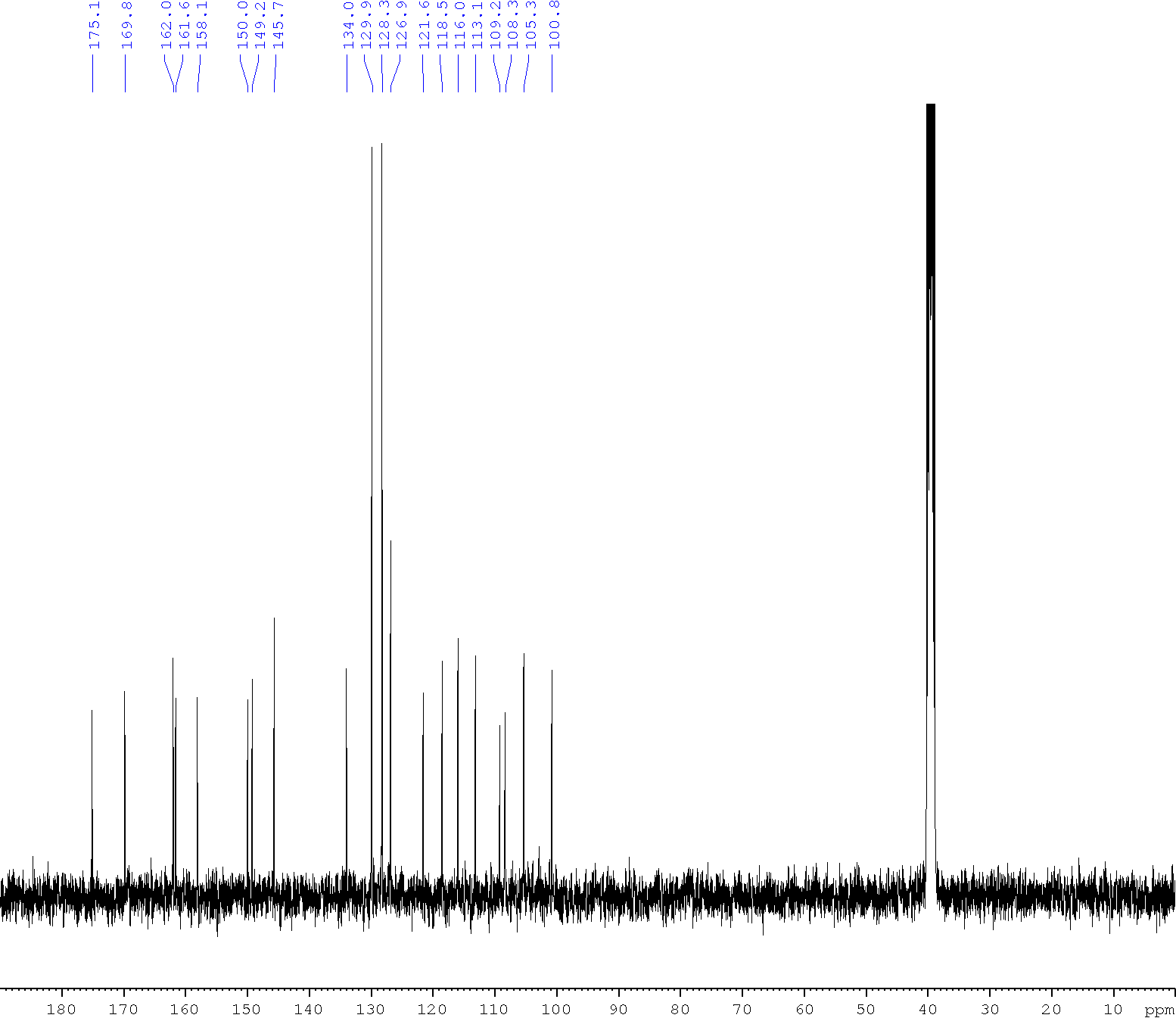


2-(2ʹ,2ʹ-Diphenylbenzo[d][1ʹ,3ʹ]dioxol-5ʹ-yl)-5,7-dihydroxy-4H-chromen-4-one (5)

To a stirred solution of luteolin (1.0 g, 3.49 mmol) in diphenylether (50 mL) at 60 °C was added dichlorodiphenylmethane (1.0 mL, 5.24 mmol). The reaction mixture was then stirred at 175 °C for 24 h. The mixture was cooled to r.t. and petroleum ether (50 mL) was added to precipitate the crude product. The precipitate was filtered and further washed with petroleum ether (50 mL). The crude solid was dissolved in EtOAc and the solvent removed in vacuo. The resulting crude product was purified by flash chromatography (4:1 PE:EtOAC) to give the *title compound* (0.61 g, 39%) as a yellow solid.

**R_f_:** 0.39 (3:1 PE:EtOAC)

**M.P.:** 155 – 158 °C

**δ_H_** (400 MHz; d_6_-DMSO): 6.20 (1H, d, *J* = 2.0 Hz, 6-H), 6.52 (1H, d, *J* = 2.0, 8-H), 6.89 (1H, s, 3-H), 7.23 (1H, d, *J* = 8.3 Hz, 7ʹ-H), 7.42 – 7.49 (6H, m, 2ʹʹ-H and 3ʹʹ-H), 7.52 – 7.57 (4H, m, 2ʹʹ-H), 7.71 (1H, dd, *J* = 8.3, 1.8 Hz, 6ʹ-H), 7.79 (1H, d, *J* = 1.8 Hz, 4ʹ-H), 10.89 (1H, s, 7-OH), 12.87 (1H, s, 5-OH)

**δ_C_** (100 MHz; d_6_-DMSO): 94.1 (C-8), 98.3 (C-6), 103.1 (C-4a), 104.2 (C-3), 106.9 (C-4ʹ), 109.2 (C-7ʹ), 117.5 (C-2ʹ), 122.2 (C-6ʹ), 125.1 (C-5ʹ), 125.8 (C-2ʹʹ), 128.7, 129.6 (C-3ʹʹ and C-4ʹʹ), 139.1 (C-1ʹʹ), 147.3 (C-3ʹa), 149.5 (C-7ʹa), 157.3 (C-8a), 161.4 (C-5), 162.8 (C-2), 164.3 (C-7), 181.8 (C-4)

**IR:** ν_max_/cm^-1^; 680, 695, 760, 777, 811, 836, 912, 948, 1017, 1043, 1093, 1169, 1209, 1251, 1303, 1338, 1372, 1415, 1441, 1485, 1560, 1604, 1649, 1737, 2630, 2925

**HRMS (ESI^+^):** Found (MNa^+^) 473.0984, C_28_H_18_NaO­_6_ requires 473.0996

The ^1^H and ^13^C NMR δ values were in agreement with literature.^2^

2-(2ʹ,2ʹ-Diphenylbenzo[d][1ʹ,3ʹ]dioxol-5ʹ-yl)-5-hydroxy-4-oxo-4H-chromen-7-yl palmitate (6)

The reaction was carried out according to general procedure A with **5** (0.3 g, 0.67 mmol), Et_3_N (0.14 mL, 1.00 mmol) and palmitoyl chloride (0.18 mL, 0.60 mmol). The product was purified by flash chromatography (4:1 PE:EtOAC) to give the *title compound* (0.35 g, 75%) as a yellow solid.

**R_f_:** 0.90 (4:1 PE:EtOAC)

**M.P.:** 88 – 91 °C

**δ_H_** (400 MHz; CDCl_3_): 0.88 (3H, t, *J* = 7.0 Hz, 16ʹʹ-H), 1.24 – 1.39 (22H, broad m, 5ʹʹ-H, 6ʹʹ-H, 7ʹʹ-H, 8ʹʹ-H, 9ʹʹ-H, 10ʹʹ-H, 11ʹʹ-H, 12ʹʹ-H, 13ʹʹ-H, 14ʹʹ-H and 15ʹʹ-H), 1.40 – 1.47 (2H, m, 4ʹʹ-H), 1.76 (2H, p, *J* = 7.5 Hz, 3ʹʹ-H), 2.58 (2H, t, *J* = 7.5 Hz, 2ʹʹ-H), 6.54 (1H, d, *J* = 2.0 Hz, 6-H), 6.58 (1H, s, 3-H), 6.80 (1H, d, *J* = 2.0 Hz, 8-H), 7.00 (1H, d, *J* = 8.0 Hz, 7ʹ-H), 7.39 – 7.44 (7H, m, 4ʹ-H, 3ʹʹʹ-H and 4ʹʹʹ-H), 7.47 (1H, dd, *J* = 8.0, 2.0 Hz, 6ʹ-H), 7.56 – 7.60 (4H, m, 2ʹʹʹ-H), 12.77 (1H, s, 5-OH)

**δ_C_** (100 MHz; CDCl_3_): 14.3 (C-16ʹʹ), 22.8 (C-15ʹʹ), 25.0 (C-3ʹʹ), 29.2, 29.4, 29.5, 29.6, 29.7, 29.8, 29.8 (C-4ʹʹ, C-5ʹʹ, C-6ʹʹ, C-7ʹʹ, C-8ʹʹ, C-9ʹʹ, C-10ʹʹ, C-11ʹʹ, C-12ʹʹ and C-13ʹʹ), 32.1 (C-14ʹʹ), 34.6 (C-2ʹʹ), 101.0 (C-8), 105.1 (C-3), 105.5 (C-6), 106.6 (C-4ʹ), 108.9 (C-4a), 109.2 (C-7ʹ), 118.6 (C-7ʹ), 122.0 (C-6ʹ), 125.1 (C-5ʹ), 126.4 (C-2ʹʹʹ), 128.6, 129.6 (C-3ʹʹʹ and C-4ʹʹʹ), 139.6 (C-1ʹʹʹ), 148.3 (C-3ʹa), 150.8 (C-7ʹa), 156.1 (C-7), 156.8 (C-8a), 162.0 (C-5), 164.6, (C-2), 171.4 (C-1ʹʹ), 182.9 (C-4)

**IR:** ν_max_/cm^-1^; 691, 696, 722, 752, 784, 814, 848, 863, 884, 905, 917, 950, 987, 1019, 1046, 1094, 1128, 1190, 1214, 1244, 1302, 1327, 1370, 1408, 1496, 1619, 1647, 1765, 2851, 2920, 3070

**HRMS (ESI^+^):** Found (MNa^+^) 711.3260, C_44_H_48_NaO­_7_ requires 711.3292

**Supplementary Figure 18:** ^1^H and ^13^C NMR of **6**


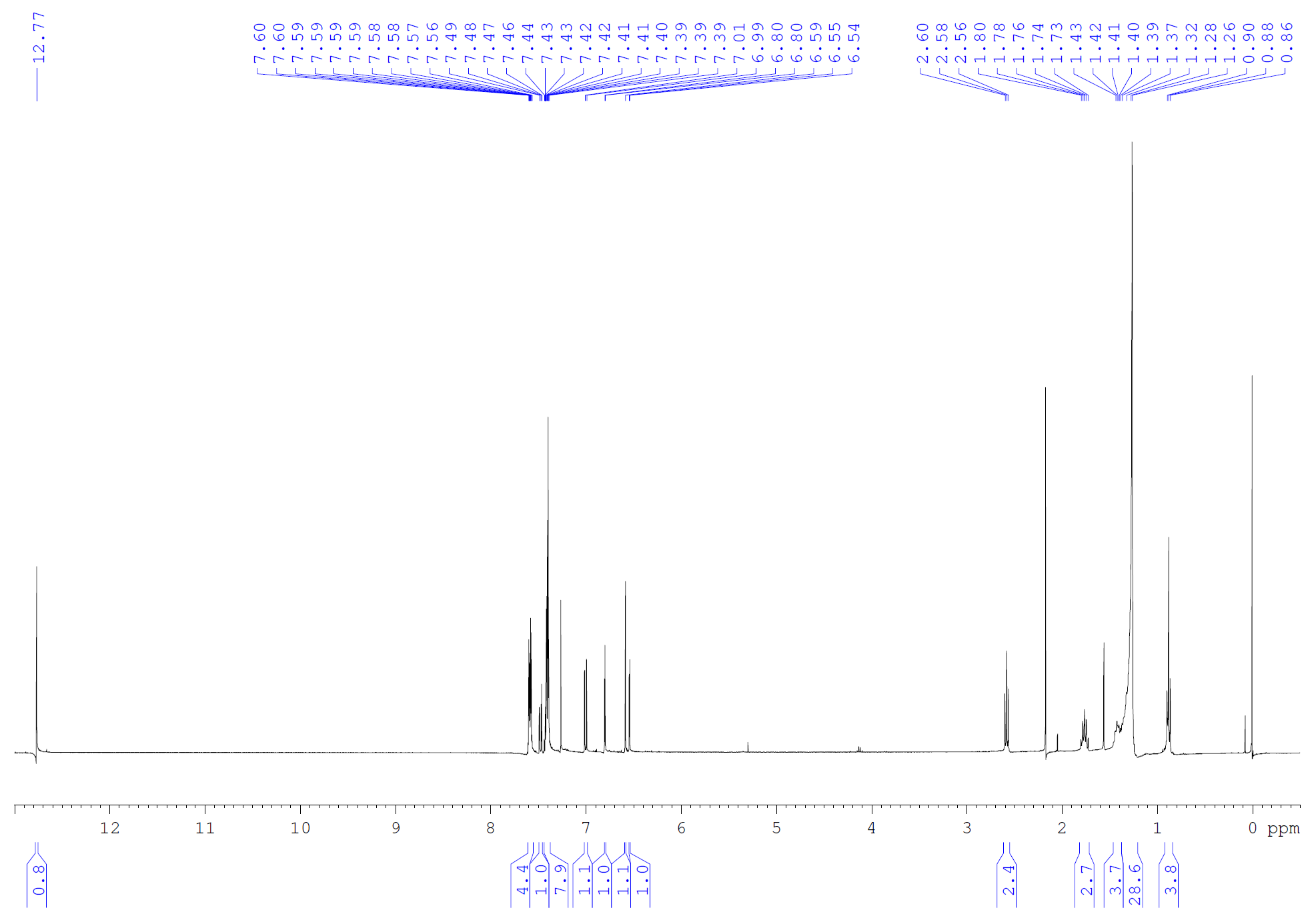


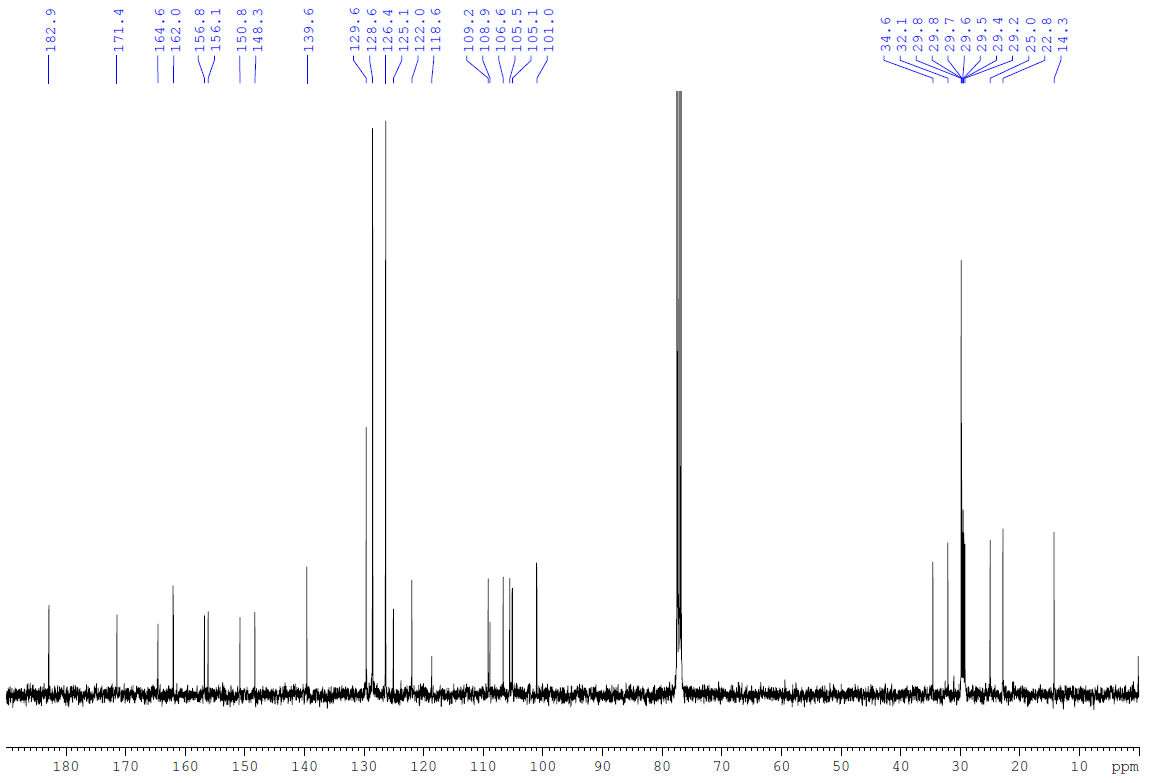


**2-(3ʹ,4ʹ-Dihydroxyphenyl)-5-hydroxy-4-oxo-4H-chromen-7-yl palmitate (7)**

The reaction was carried out according to general procedure B with **6** (0.35 g, 0.50 mmol) and 10% Pd/C (0.14 g, 0.10 mmol). The reaction was stirred for 4 d. The product was purified by flash chromatography (3:1 PE:EtOAC) to give the *title compound* (90 mg, 34%) as a yellow solid.

**R_f_:** 0.28 (2:1 PE:EtOAC)

**M.P.:** 202 – 205 °C

**δ_H_** (400 MHz; d_6_-DMSO): 0.84 (3H, t, *J* = 6.8 Hz, 16ʹʹ-H), 1.17 – 1.32 (22H, broad m, 5ʹʹ-H, 6ʹʹ-H, 7ʹʹ-H, 8ʹʹ-H, 9ʹʹ-H, 10ʹʹ-H, 11ʹʹ-H, 12ʹʹ-H, 13ʹʹ-H, 14ʹʹ-H and 15ʹʹ-H), 1.34 – 1.36 (2H, m, 4ʹʹ-H), 1.65 (2H, p, *J* = 7.4 Hz, 3ʹʹ-H), 2.60 (2H, t, *J* = 7.4 Hz, 2ʹʹ-H), 6.60 (1H, d, *J* = 2.0 Hz, 6-H), 6.83 (1H, s, 3-H), 6.90 (1H, d, *J* = 8.4 Hz, 5ʹ-H), 7.01 (1H, d, *J* = 2.0 Hz, 8-H), 7.44 (1H, d, *J* = 2.4 Hz, 2ʹ-H), 7.47 (1H, dd, *J* = 8.4, 2.4 Hz, 6ʹ-H), 9.39 (1H, s, 4ʹ-OH), 10.03 (1H, s, 3ʹ-OH), 13.03 (1H, s, 5-OH)

**δ_C_** (100 MHz; d_6_-DMSO): 13.9 (C-16ʹʹ), 22.1 (C-15ʹʹ), 24.1 (C-3ʹʹ), 28.3, 28.6, 28.8, 29.0, 29.0 (C-4ʹʹ, C-5ʹʹ, C-6ʹʹ, C-7ʹʹ, C-8ʹʹ, C-9ʹʹ, C-10ʹʹ, C-11ʹʹ, C-12ʹʹ and C-13ʹʹ), 31.2 (C-14ʹʹ), 33.5 (C-2ʹʹ), 101.4 (C-8), 103.4 (C-3), 105.1 (C-6), 108.0 (C-4a), 113.7 (C-2ʹ), 116.0 (C-5ʹ), 119.3 (C-6ʹ), 121.1 (C-1ʹ), 145.8 (C-3ʹ), 150.2 (C-4ʹ), 155.7 (C-7), 156.1 (C-8a), 160.8 (C-5), 165.0 (C-2), 171.1 (C-1ʹʹ), 182.2 (C-4)

**IR:** ν_max_/cm^-1^; 687, 720, 746, 780, 792, 815, 828, 840, 860, 875, 916, 994, 1031, 1094, 1135, 1190, 1219, 1260, 1298, 1344, 1360, 1415, 1462, 1495, 1563, 1600, 1646, 1764, 2850, 2918, 3414

**HRMS (ESI^+^):** Found (MNa^+^) 547.2660, C_31_H_40_NaO­_7_ requires 547.2666

**Supplementary Figure 19:** ^1^H and ^13^C NMR of **7**


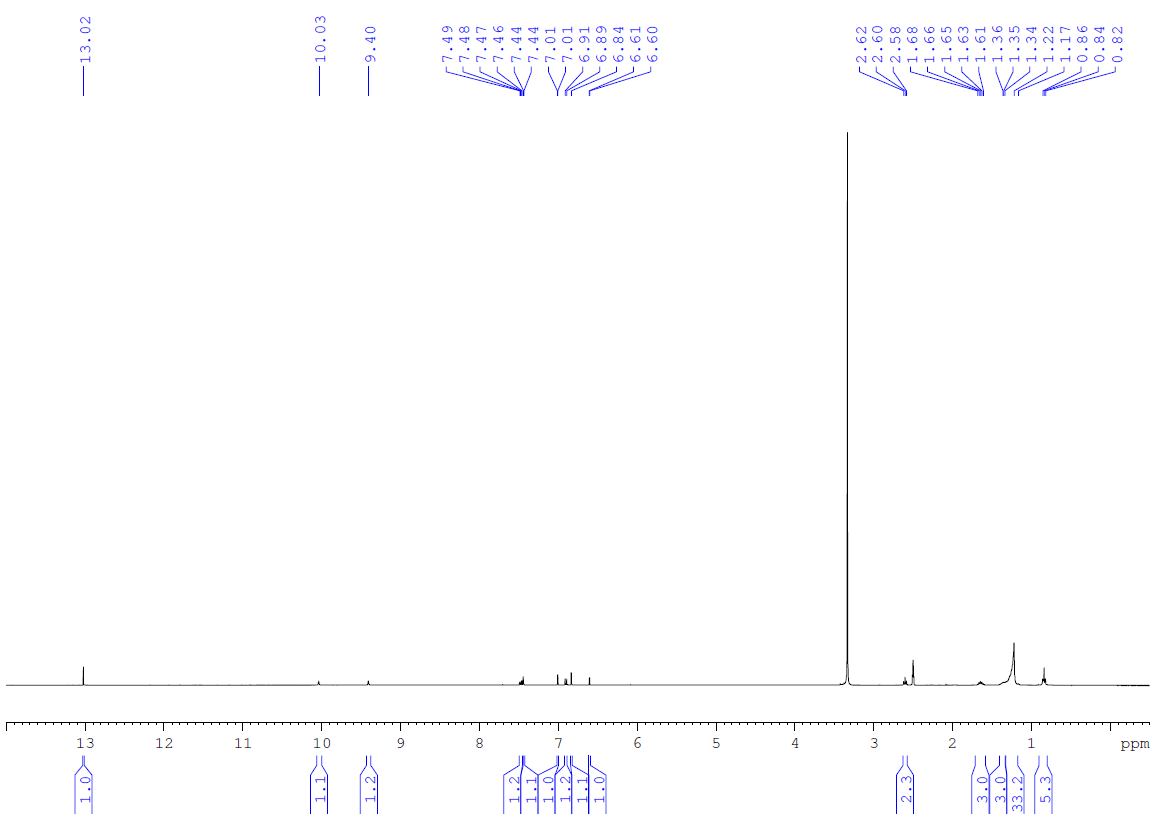


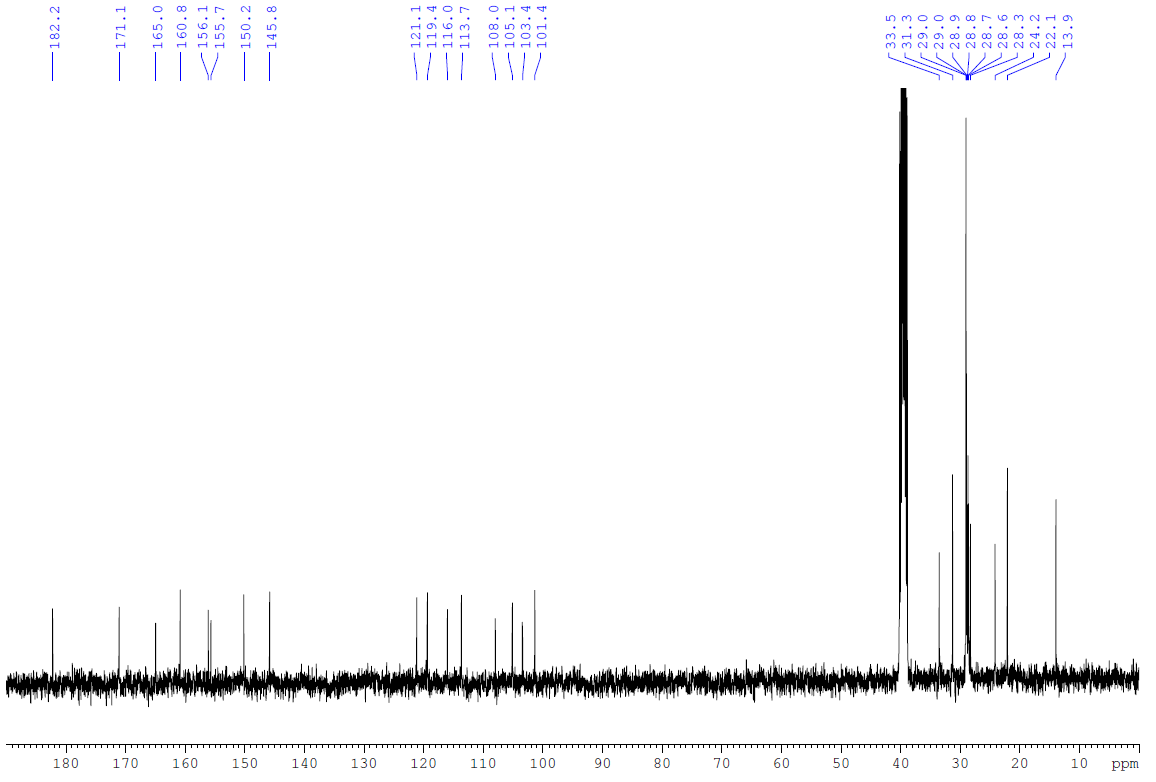


3. Radical scavenging activity data

**Supplementary Table 1:** Trolox standard curves against ABTS

|  | **Trolox (µM)** | **% ABTS  Scavenged** | **Standard Curve** |
| --- | --- | --- | --- |
| **First Assay** | 40 | 3.4% |  |
|  | 60 | 8.9% |  |
|  | 80 | 18.1% |  |
|  | 100 | 24.1% |  |
|  | 200 | 43.0% |  |
|  | 400 | 100.0% |  |
| **Second Assay** | 40 | 6.0% |  |
|  | 60 | 13.4% |  |
|  | 80 | 18.5% |  |
|  | 100 | 19.0% |  |
|  | 200 | 52.5% |  |
|  | 400 | 100.0% |  |
| **Third Assay** | 40 | 1.4% |  |
|  | 60 | 5.8% |  |
|  | 80 | 10.6% |  |
|  | 100 | 17.6% |  |
|  | 200 | 51.9% |  |
|  | 400 | 100.0% |  |

**Supplementary Table 2:** Trolox standard curves against DPPH

|  | **Trolox (µM)** | **% DPPH Scavenged** | **Standard Curve** |
| --- | --- | --- | --- |
| **First Assay** | 100 | 1.0% |  |
|  | 200 | 17.5% |  |
|  | 400 | 44.4% |  |
|  | 600 | 73.5% |  |
|  | 800 | 90.7% |  |
| **Second Assay** | 100 | 7.7% |  |
|  | 200 | 21.3% |  |
|  | 400 | 38.7% |  |
|  | 600 | 73.6% |  |
|  | 800 | 85.1% |  |
| **Third Assay** | 100 | 9.8% |  |
|  | 200 | 21.4% |  |
|  | 400 | 43.1% |  |
|  | 600 | 68.8% |  |
|  | 800 | 86.5% |  |

**Supplementary Table 3:** Radical scavenging activity data of luteolin and derivatives against ABTS

|  | **µM** | **First Assay** | **Second Assay** | **Third Assay** | **Average %** | **SE** | **Two sample *p*-value  (Two tail, equal or unequal variance)** |
| --- | --- | --- | --- | --- | --- | --- | --- |
|  |  | **% ABTS  Scavenged** | **% ABTS  Scavenged** | **% ABTS  Scavenged** |  |  |  |
| **Luteolin** | **20** | 24.8% | 19.3% | 15.1% | 19.76% | 2.81% | - |
|  | **40** | 33.3% | 40.9% | 38.7% | 37.64% | 2.26% | - |
|  | **60** | 54.5% | 62.8% | 61.3% | 59.55% | 2.55% | - |
|  | **80** | 69.5% | 77.4% | 80.1% | 75.66% | 3.19% | - |
|  | **100** | 89.7% | 92.9% | 95.7% | 92.76% | 1.73% | - |
|  | **150** | 100.0% | 100.0% | 100.0% | 100.00% | 0.00% | - |
| **4a** | **20** | 22.7% | 15.5% | 15.1% | 17.75% | 2.47% | 0.62 |
|  | **40** | 46.1% | 34.0% | 34.8% | 38.32% | 3.90% | 0.89 |
|  | **60** | 53.9% | 56.7% | 57.6% | 56.07% | 1.12% | 0.28 |
|  | **80** | 77.5% | 71.1% | 74.5% | 74.37% | 1.86% | 0.74 |
|  | **100** | 100.0% | 86.3% | 92.2% | 92.83% | 3.97% | 0.99 |
|  | **150** | 100.0% | 99.4% | 98.5% | 99.30% | 0.43% | 0.18 |
| **4b** | **20** | 36.7% | 11.1% | 16.5% | 21.44% | 7.80% | 0.85 |
|  | **40** | 50.5% | 35.3% | 38.7% | 41.48% | 4.60% | 0.50 |
|  | **60** | 59.6% | 55.5% | 57.6% | 57.57% | 1.19% | 0.52 |
|  | **80** | 76.3% | 68.0% | 68.1% | 70.80% | 2.72% | 0.31 |
|  | **100** | 92.0% | 85.6% | 92.7% | 90.10% | 2.26% | 0.40 |
|  | **150** | 96.9% | 100.0% | 98.1% | 98.34% | 0.90% | 0.14 |
| **4c** | **20** | 34.4% | 19.7% | 16.5% | 23.52% | 5.53% | 0.58 |
|  | **40** | 48.0% | 38.5% | 38.7% | 41.76% | 3.13% | 0.35 |
|  | **60** | 57.2% | 55.0% | 58.2% | 56.79% | 0.94% | 0.37 |
|  | **80** | 72.4% | 71.4% | 68.3% | 70.69% | 1.23% | 0.22 |
|  | **100** | 92.3% | 99.6% | 91.8% | 94.53% | 2.52% | 0.59 |
|  | **150** | 100.0% | 99.7% | 96.9% | 98.87% | 0.99% | 0.32 |
| **4d** | **20** | 33.2% | 16.4% | 17.7% | 22.43% | 5.42% | 0.68 |
|  | **40** | 38.9% | 38.9% | 34.6% | 37.46% | 1.41% | 0.95 |
|  | **60** | 54.1% | 49.2% | 46.9% | 50.08% | 2.12% | 0.05 |
|  | **80** | 71.1% | 57.9% | 67.8% | 65.59% | 3.98% | 0.12 |
|  | **100** | 91.8% | 94.0% | 73.7% | 86.52% | 6.43% | 0.40 |
|  | **150** | 96.1% | 99.7% | 97.7% | 97.85% | 1.04% | 0.11 |
| **4e** | **20** | 35.2% | 16.0% | 19.0% | 23.38% | 5.95% | 0.61 |
|  | **40** | 48.1% | 32.5% | 37.8% | 39.44% | 4.58% | 0.74 |
|  | **60** | 61.7% | 52.9% | 58.7% | 57.80% | 2.59% | 0.65 |
|  | **80** | 75.3% | 65.3% | 76.3% | 72.30% | 3.52% | 0.52 |
|  | **100** | 93.7% | 82.9% | 90.5% | 89.03% | 3.22% | 0.37 |
|  | **150** | 99.9% | 99.6% | 99.2% | 99.55% | 0.19% | 0.07 |

| **4f** | **20** | 21.6% | 4.5% | 17.7% | 14.59% | 5.20% | 0.43 |
| --- | --- | --- | --- | --- | --- | --- | --- |
|  | **40** | 24.2% | 18.1% | 33.8% | 25.38% | 4.56% | 0.07 |
|  | **60** | 44.6% | 38.4% | 50.7% | 44.54% | 3.55% | 0.03 |
|  | **80** | 46.6% | 43.6% | 62.5% | 50.90% | 5.89% | 0.02 |
|  | **100** | 57.6% | 66.5% | 83.1% | 69.05% | 7.49% | 0.04 |
|  | **150** | 84.4% | 77.9% | 99.1% | 87.13% | 6.27% | 0.11 |
| **4g** | **20** | **12.4%** | 19.2% | 18.0% | 16.53% | 2.10% | 0.41 |
|  | **40** | **27.9%** | 24.7% | 37.3% | 29.96% | 3.77% | 0.16 |
|  | **60** | **39.0%** | 33.5% | 52.7% | 41.71% | 5.71% | 0.05 |
|  | **80** | **45.2%** | 50.6% | 69.1% | 54.96% | 7.22% | 0.06 |
|  | **100** | **52.0%** | 55.7% | 81.0% | 62.89% | 9.12% | 0.08 |
|  | **150** | **65.2%** | 73.9% | 97.4% | 78.81% | 9.62% | 0.09 |
| **4h** | **20** | **14.1%** | 15.9% | - | 15.02% | 0.74% | 0.29 |
|  | **40** | **26.8%** | 35.4% | - | 31.11% | 3.54% | 0.23 |
|  | **60** | **42.5%** | 51.1% | - | 46.78% | 3.51% | 0.07 |
|  | **80** | **60.8%** | 58.3% | - | 59.51% | 1.02% | 0.03 |
|  | **100** | **73.4%** | 70.3% | - | 71.87% | 1.26% | 0.00 |
|  | **150** | **80.7%** | 90.0% | - | 85.36% | 3.83% | 0.02 |
| **4i** | **20** | **10.5%** | 4.8% | 9.2% | 8.17% | 1.70% | 0.02 |
|  | **40** | **20.4%** | 18.6% | 30.6% | 23.19% | 3.72% | 0.03 |
|  | **60** | **32.6%** | 34.2% | 96.4% | 54.41% | 21.02% | 0.83 |
|  | **80** | **45.6%** | 49.5% | 68.8% | 54.62% | 7.18% | 0.06 |
|  | **100** | **58.0%** | 61.2% | 80.8% | 66.67% | 7.13% | 0.02 |
|  | **150** | **69.5%** | 79.3% | 99.9% | 82.92% | 8.96% | 0.13 |

**Supplementary Table 4:** Radical scavenging activity data of luteolin and derivatives against DPPH

|  | **µM** | **First Assay** | **Second Assay** | **Third Assay** | **Average**  **%** | **SE** | **Two sample *p*-value  (Two tail, equal or unequal variance)** |
| --- | --- | --- | --- | --- | --- | --- | --- |
|  |  | **% DPPH  Scavenged** | **% DPPH  Scavenged** | **% DPPH  Scavenged** |  |  |  |
| **Luteolin** | **50** | 13.0% | 10.6% | 12.8% | 12.13% | 0.78% | - |
|  | **100** | 31.6% | 39.6% | 32.9% | 34.70% | 2.48% | - |
|  | **200** | 60.6% | 58.9% | 54.7% | 58.05% | 1.75% | - |
|  | **300** | 87.8% | 76.9% | 84.2% | 82.96% | 3.21% | - |
|  | **400** | 89.7% | 84.5% | 88.7% | 87.61% | 1.61% | - |
|  | **500** | 94.1% | 94.6% | 90.5% | 93.09% | 1.30% | - |
| **4a** | **50** | 12.1% | 10.6% | 8.8% | 10.49% | 0.95% | 0.25 |
|  | **100** | 28.4% | 39.6% | 30.0% | 32.67% | 3.49% | 0.66 |
|  | **200** | 52.7% | 58.9% | 57.9% | 56.51% | 1.91% | 0.58 |
|  | **300** | 70.1% | 76.9% | 71.0% | 72.66% | 2.13% | 0.06 |
|  | **400** | 83.1% | 84.5% | 79.6% | 82.38% | 1.45% | 0.07 |
|  | **500** | 91.1% | 88.9% | 86.1% | 88.71% | 1.46% | 0.09 |
| **4b** | **50** | 13.7% | 8.3% | 21.1% | 14.37% | 3.72% | 0.59 |
|  | **100** | 32.0% | 34.4% | 34.2% | 33.54% | 0.80% | 0.68 |
|  | **200** | 59.7% | 81.4% | 60.7% | 67.30% | 7.08% | 0.27 |
|  | **300** | 76.3% | 90.4% | 73.9% | 80.22% | 5.14% | 0.67 |
|  | **400** | 91.5% | 91.0% | 85.9% | 89.46% | 1.80% | 0.48 |
|  | **500** | 92.9% | 91.7% | 87.0% | 90.54% | 1.80% | 0.31 |
| **4c** | **50** | 12.5% | 8.3% | 13.7% | 11.48% | 1.62% | 0.74 |
|  | **100** | 23.0% | 34.4% | 32.0% | 29.78% | 3.49% | 0.31 |
|  | **200** | 59.1% | 81.4% | 63.7% | 68.08% | 6.81% | 0.23 |
|  | **300** | 82.3% | 90.4% | 78.3% | 83.67% | 3.55% | 0.89 |
|  | **400** | 91.7% | 91.0% | 91.7% | 91.48% | 0.22% | 0.13 |
|  | **500** | 92.2% | 91.7% | 92.9% | 92.30% | 0.34% | 0.59 |
| **4d** | **50** | 17.9% | 10.2% | 14.4% | 14.18% | 2.22% | 0.43 |
|  | **100** | 37.8% | 37.2% | 31.6% | 35.52% | 1.98% | 0.81 |
|  | **200** | 59.7% | 62.4% | 49.7% | 57.27% | 3.86% | 0.86 |
|  | **300** | 81.7% | 89.5% | 69.3% | 80.13% | 5.88% | 0.69 |
|  | **400** | 90.8% | 90.7% | 72.1% | 84.55% | 6.21% | 0.66 |
|  | **500** | 92.7% | 90.8% | 88.3% | 90.62% | 1.25% | 0.24 |
| **4e** | **50** | 18.6% | 13.2% | 14.5% | 15.43% | 1.63% | 0.14 |
|  | **100** | 29.5% | 38.2% | 31.5% | 33.07% | 2.64% | 0.68 |
|  | **200** | 57.9% | 69.4% | 55.3% | 60.87% | 4.34% | 0.58 |
|  | **300** | 80.0% | 82.5% | 73.5% | 78.66% | 2.70% | 0.36 |
|  | **400** | 91.6% | 88.5% | 83.1% | 87.76% | 2.48% | 0.96 |
|  | **500** | 90.6% | 89.6% | 88.5% | 89.60% | 0.62% | 0.07 |

| **4f** | **50** | 10.1% | 8.9% | 13.2% | 10.69% | 1.28% | 0.40 |
| --- | --- | --- | --- | --- | --- | --- | --- |
|  | **100** | 39.3% | 34.9% | 31.5% | 35.23% | 2.25% | 0.88 |
|  | **200** | 64.4% | 48.8% | 57.1% | 56.75% | 4.51% | 0.80 |
|  | **300** | 79.9% | 70.6% | 76.3% | 75.60% | 2.70% | 0.15 |
|  | **400** | 87.2% | 86.0% | 80.3% | 84.52% | 2.12% | 0.31 |
|  | **500** | 88.9% | 91.8% | 89.0% | 89.89% | 0.97% | 0.12 |
| **4g** | **50** | 5.0% | 20.9% | 16.3% | 14.05% | 4.72% | 0.73 |
|  | **100** | 14.4% | 34.1% | 30.1% | 26.23% | 6.00% | 0.26 |
|  | **200** | 50.4% | 64.6% | 55.4% | 56.80% | 4.16% | 0.80 |
|  | **300** | 38.4% | 48.5% | 74.9% | 53.89% | 10.88% | 0.06 |
|  | **400** | 59.9% | 72.5% | 83.3% | 71.88% | 6.75% | 0.09 |
|  | **500** | 67.0% | 78.1% | 91.0% | 78.71% | 6.94% | 0.11 |
| **4h** | **50** | 13.0% | 13.2% | 14.6% | 13.58% | 0.51% | 0.19 |
|  | **100** | 25.0% | 27.4% | 31.4% | 27.94% | 1.88% | 0.10 |
|  | **200** | 47.7% | 49.9% | 47.3% | 48.31% | 0.81% | 0.01 |
|  | **300** | 63.4% | 69.9% | 63.7% | 65.67% | 2.11% | 0.01 |
|  | **400** | 68.3% | 80.8% | 75.2% | 74.77% | 3.62% | 0.03 |
|  | **500** | 77.1% | 87.9% | 78.1% | 81.01% | 3.44% | 0.03 |
| **4i** | **50** | 11.7% | 13.0% | - | 12.31% | 0.53% | 0.88 |
|  | **100** | 31.4% | 32.3% | - | 31.83% | 0.35% | 0.44 |
|  | **200** | 42.0% | 49.6% | - | 45.83% | 3.10% | 0.04 |
|  | **300** | 54.6% | 62.0% | - | 58.32% | 3.02% | 0.02 |
|  | **400** | 61.8% | 66.7% | - | 64.25% | 2.02% | 0.00 |
|  | **500** | 72.1% | 76.1% | - | 74.10% | 1.65% | 0.00 |

4. Supplementary references:

(1) Zhang, J.; Liu, X.; Lei, X.; Wang, L.; Guo, L.; Zhao, G.; Lin, G. Discovery and Synthesis of Novel Luteolin Derivatives as DAT Agonists. *Bioorg. Med. Chem.* **2010**, *18* (22), 7842–7848. https://doi.org/10.1016/j.bmc.2010.09.049.

(2) Nilsson, L.; Larsson, A.; Begum, A.; Iakovleva, I.; Carlsson, M.; Brännström, K.; Sauer-Eriksson, A. E.; Olofsson, A. Modifications of the 7-Hydroxyl Group of the Transthyretin Ligand Luteolin Provide Mechanistic Insights into Its Binding Properties and High Plasma Specificity. *PLOS ONE* **2016**, *11* (4), e0153112. https://doi.org/10.1371/journal.pone.0153112.
